# Experiences of self-management behaviors during adaptation to permanent enterostomy among older patients: A qualitative study

**DOI:** 10.1371/journal.pone.0360643

**Published:** 2026-10-29

**Authors:** Zhichao Duan, Fang Wu

**Affiliations:** 1 School of Nursing, Inner Mongolia Medical University, Hohhot, Inner Mongolia, China; 2 Department of Gastrointestinal Surgery, Affiliated Hospital of Inner Mongolia Medical University, Hohhot, Inner Mongolia, China; Jinnah Sindh Medical University, National Institute of Child Health, PAKISTAN

## Abstract

**Object:**

This study aimed to explore the experiences and perceived changes in self-management behaviors among older patients with a permanent stoma during adaptation, providing insights for the development of targeted supportive interventions.

**Methods:**

Semi-structured, in-depth interviews were conducted with 13 older colorectal cancer patients with a permanent stoma. All interviews were audio-recorded and transcribed verbatim. The data were analyzed and thematically refined using Colaizzi’s seven-step method.

**Results:**

Three overarching themes and several sub-themes were identified. Theme one, decision-making dilemmas and uncertainty, included insufficient and poorly tailored decision support resources and challenges in processing complex self-management information. Theme two, reconstructing daily life and identity after stoma formation, encompassed disruptions in body image and daily routines, emotional struggles between rejection and acceptance, and the development of a new normal in living with a stoma. Theme three, developing resilience through accumulated experience and self-management practice, involved learning through repeated self-adjustment, drawing on previous life experiences as coping resources, and redefining life priorities after illness.

**Conclusion:**

The findings suggest that older patients living with a permanent stoma experience self-management as an ongoing process involving decisional challenges, psychological adjustment, and the gradual reconstruction of resilience. Healthcare professionals should provide continuous decision support, psychological care, and family-inclusive interventions while adopting a strength-based approach that recognizes patients’ existing abilities and lived experiences. Supporting patients in reflecting on their adaptation experiences and accumulated coping strategies may enhance self-efficacy and facilitate sustained self-management.

## 1. Introduction

Colorectal cancer (CRC) is one of the most common malignancies and poses a significant threat to human health because of its high incidence and mortality rates. According to Global Cancer Statistics 2022 released by the International Agency for Research on Cancer (IARC) [1], newly diagnosed cases of CRC accounted for 9.6% of all cancer cases, and CRC-related deaths constituted 9.3% of all cancer-related deaths. In China, an estimated 517,106 new cases of CRC and 240,010 CRC-related deaths occurred in 2022, accounting for approximately 10.7% and 9.3% of all new cancer cases and cancer deaths, respectively [2]. For selected patients with CRC, ostomy surgery may be required depending on disease characteristics and the surgical approach. It involves creating an opening in the abdominal wall through which a segment of the intestine is brought to the skin surface to divert fecal elimination [3]. This procedure effectively alleviates symptoms, prevents disease progression, and extends survival [4].However, owing to factors such as disease advancement or the specific surgical approach employed, some patients require a stoma for an extended period—or even for life—resulting in a permanent stoma.

Self-management is a multidimensional concept that encompasses the behaviors, cognitive processes, and emotional strategies individuals use to manage chronic health conditions and their consequences [5]. In the present study, self-management among older patients with a permanent stoma refers to the ongoing efforts to manage stoma care, prevent complications, seek information, make health-related decisions, adjust daily activities, and cope with the psychological and social changes of living with a stoma. Self-care refers to specific care activities, psychological adaptation to adjustment to illness-related changes, independence to the extent of performing activities without assistance, and self-efficacy to confidence in performing self-management behaviors [6–8]. These concepts are related to, but distinct from, self-management [5,9].

After ostomy surgery, the demands of self-management extend to every dimension of daily life, including dietary adjustments, stoma care, and social participation, and rely not only on relevant knowledge and skills but, more importantly, on an individual’s ongoing behavioral adaptation and decision-making across varying contexts [10,11]. However, with the widespread adoption of Enhanced Recovery After Surgery (ERAS) protocols, hospital stays have become progressively shorter; consequently, some patients have not yet fully developed self-management capabilities by the time of discharge. Furthermore, during the early postoperative period, most patients struggle to adapt quickly to the altered body image associated with the stoma, and their awareness of the need to assume self-management responsibilities proactively remains relatively limited [12]. These challenges may be particularly relevant for older adults, who may experience greater difficulty acquiring new stoma-care knowledge and skills, while also drawing on accumulated life experience and established coping strategies as potential personal resources for managing long-term health changes [13,14]. Furthermore, stoma care procedures are inherently complex and technically demanding; consequently, older patients may find it challenging to transition from dependent care to independent self-management within a short period [15].

Previous studies have examined enterostomy self-management in relation to self-care, quality of life, psychological adaptation, support needs, and associated factors [10,16].. However, less is known about how older adults with permanent stomas themselves perceive and make sense of changes in self-management during adaptation, particularly in relation to decision-making, problem-solving, repeated adjustment, and accumulated life experience [17]. While previous research has largely focused on self-management levels, difficulties, and support needs, the present study further explores the lived meanings and perceived changes underlying these experiences from the participants’ own perspectives. Therefore, this study adopted a descriptive phenomenological approach to explore older adults’ lived experiences and perceived changes in self-management during adaptation to a permanent stoma.

## 2. Methods

### 2.1. Study design

This study employed a descriptive phenomenological approach to explore the lived experiences and meanings of self-management among older adults with permanent stomas during adaptation. This approach was selected to understand participants’ subjective experiences and the meanings they attributed to self-management in everyday life. Because each participant was interviewed once, the study focused on participants’ retrospective accounts and perceptions of changes in self-management from stoma formation to the time of interview, rather than directly observing changes across different stages of adaptation. Through semi-structured in-depth interviews, the study investigates the self-management behaviors of older patients with permanent intestinal stomas during their long-term adaptation process. The research was conducted from March 25 to May 5, 2026, within the Department of Gastrointestinal Surgery and the Stoma Clinic of a tertiary hospital in the Inner Mongolia Autonomous Region, China. The hospital provides specialized care for patients with colorectal diseases, and the Stoma Clinic offers postoperative follow-up, stoma care education, and complication management services for patients with enterostomies.

### 2.2. Inclusion and exclusion criteria

Inclusion Criteria: ①Age ≥ 60 years;②Willingness to reflect deeply on interview questions and engage in self-disclosure;③Clinically diagnosed and having undergone permanent intestinal stoma surgery;④Postoperative period≥3 months, and currently in the post-discharge home adaptation phase. Exclusion Criteria:①Subjects experiencing severe emotional fluctuations or withdrawing from the study midway;②Acute deterioration of the medical condition during the course of the study.

Participants varied in the duration since stoma formation. This variation was intentionally retained to capture a range of retrospective adaptation experiences and perspectives on self-management rather than to compare predefined postoperative stages. Participants were therefore not grouped or analyzed according to stoma duration; instead, variation in duration was treated as a source of experiential diversity within the phenomenological sample.

### 2.3. Ethical considerations

This study was approved by the Ethics Committee of the Affiliated Hospital of Inner Mongolia Medical University (KY2026017). Prior to data collection, all study participants provided written informed consent. They had the right to withdraw from the study at any time during the interviews.

### 2.4. Data collection

Participants were recruited through posters displayed at the stoma outpatient clinic. During the recruitment period, 18 individuals expressed interest in the study and were screened for eligibility. Of these, 15 met the inclusion criteria, 1 declined participation, and 1 was excluded because of a physical condition that prevented participation in the interview. A total of 13 participants ultimately provided written informed consent and completed the interview. Purposive sampling was used to obtain information-rich accounts of self-management among older adults with permanent stomas, with attention to variation in age, sex, time since stoma formation, stoma type, stoma-related complications, and living arrangement. This approach was intended to capture diverse perspectives on self-management and adaptation rather than to achieve statistical representativeness or compare predefined postoperative stages. All interviews were conducted by the first author, a male nursing graduate student with clinical experience in gastrointestinal surgery and training in qualitative research methods, including qualitative interviewing and data analysis. The interviewer had no prior therapeutic relationship with the participants, and participation or refusal did not affect their medical care. Before each interview, the researcher explained the study purpose, procedures, voluntary participation, and confidentiality principles, and written informed consent was obtained before audio recording. Face-to-face semi-structured interviews were conducted in a private demonstration room at the stoma outpatient clinic using an interview guide. Open-ended questions and probing techniques were used to encourage participants to describe their experiences in depth, and field notes were recorded to capture contextual information and non-verbal expressions. Each interview lasted approximately 20–40 minutes, depending on participants’ willingness and the richness of their experiences. Although each participant was interviewed once, the semi-structured interview allowed participants to retrospectively describe their experiences from stoma formation to the time of interview, including their perceptions of changes in self-management, challenges encountered, and adaptation experiences. Audio recordings were transcribed verbatim and checked for accuracy after each interview. Data collection continued until data saturation was reached. The first author reviewed the interview transcripts and emerging codes throughout the data collection process to monitor whether new information or codes continued to emerge. After the 10th interview, no substantially new codes were identified. Three additional interviews were therefore conducted to confirm the stability and comprehensiveness of the emerging findings. After the 13th interview, no new codes or themes emerged. The first author presented the saturation assessment to the research team, and the team discussed and confirmed that sufficient information had been obtained to address the research question. Data collection was then terminated.

### 2.5. Develop an interview outline

The interview guide was developed based on an in-depth literature review and informed by the self-management framework [9], which emphasizes the acquisition of self-management skills, problem-solving, support utilization, and adaptation among individuals living with chronic conditions. The questions were designed to explore older patients’ experiences, challenges, behavioral changes, and support needs related to stoma self-management during adaptation to permanent stoma. The preliminary interview guide was reviewed by experts in stoma care and psychology, and appropriate modifications were made based on their feedback. Two pilot interviews were then conducted with older patients with permanent enterostomies who met the inclusion criteria but were not included in the final sample. Based on feedback from the pilot interviews, three modifications were made: (1) temporal anchoring was added to Question 1 to facilitate participants’ recall of the learning process; (2) probing prompts (e.g., “Can you describe a specific situation?”) were incorporated to encourage detailed narratives; and (3) technical terms were replaced with plain language to improve comprehensibility among older participants with varying health literacy levels. Following these refinements, the final version of the interview guide was established as follows:

(1) From discharge to the present, could you describe how you learned to manage your stoma and how your self-management behaviors changed over time?
(2) What difficulties or challenges did you encounter while learning and managing your stoma, and how did you cope with them?
(3) How do you typically perform your stoma care? Do you usually complete it yourself, or do you require assistance from others?
(4) How has undergoing stoma surgery impacted your daily life?
(5) Reflecting on the entire adaptation process, what forms of support do you consider to have been most important?
(6) What expectations or concerns do you have regarding your future life?

### 2.6. Data analysis

Following data collection, the personal information of the interviewees was anonymized and assigned numerical identifiers. Within 24 hours of the conclusion of each interview, the audio recordings were transcribed into text; simultaneously, non-verbal behaviors—such as interjections and pauses—were also transcribed alongside the verbal content. Upon completion of the initial transcription, the researcher repeatedly cross-referenced the audio recordings against the written transcripts to verify the accuracy of the transcribed data. The textual content was then imported into NVivo 11 PLUS, a qualitative data analysis software, for organization, coding, synthesis, and the extraction of key themes. This study employed Colaizzi’s phenomenological method of data analysis to organize and analyze the collected data [18].First, the researchers repeatedly read the transcripts to gain an overall understanding of participants’ accounts. Second, significant statements relevant to self-management experiences during adaptation were identified and extracted from the transcripts. Third, formulated meanings were developed from these significant statements while remaining grounded in participants’ original accounts. Fourth, related formulated meanings were organized into clusters and categories, from which preliminary themes were developed. Fifth, an exhaustive description of the phenomenon was constructed by integrating the thematic findings. Sixth, the fundamental structure of the phenomenon was identified by synthesizing the essential meanings across the themes. Finally, the resulting themes and fundamental structure were compared with the original transcripts to ensure that the findings remained consistent with participants’ accounts.

To enhance the depth and richness of the thematic findings, the researchers repeatedly reviewed the complete transcripts and examined significant statements in their original context rather than coding isolated excerpts. Open-ended questions and probing prompts were used during interviews to encourage participants to provide detailed descriptions of specific experiences, challenges, and coping responses. During analysis, emerging themes were repeatedly compared with the original transcripts to ensure that they captured the breadth and depth of participants’ accounts. Two researchers independently coded the transcripts and compared their interpretations, with discrepancies discussed within the research team until consensus was reached. Representative participant quotations were retained to illustrate and support the interpretation of each theme.

All interviews were conducted in Mandarin Chinese and transcribed verbatim in Chinese. For inclusion in the English-language manuscript, selected participant quotations were translated into English by the first author and reviewed by the second author against the original Chinese transcripts for accuracy and consistency of meaning. Any discrepancies were discussed and resolved by consensus. The analysis focused on participants’ lived experiences and perceived changes in self-management rather than on constructing a formal chronological or causal model of behavioral development. https://doi.org/10.5064/F60AEDRR.

### 2.7. Rigour and reflexivity

To enhance the methodological rigor of this qualitative study, the four criteria proposed by Lincoln and Guba, including credibility, dependability, confirmability, and transferability, were considered throughout the research process. Credibility was enhanced through purposive sampling to recruit participants with diverse experiences of living with a permanent stoma, together with independent coding and discussion within the research team. To ensure dependability, detailed records of participant recruitment, data collection, and data analysis procedures were maintained to establish an audit trail. Transcription accuracy was ensured by repeatedly reviewing transcripts against the original audio recordings.

To enhance confirmability and reflexivity, the first author maintained reflexive notes documenting personal assumptions, reflections, and methodological decisions during data collection and interpretation. Particular attention was given to the potential influence of the first author’s clinical experience in gastrointestinal surgery, male gender, and pre-existing assumptions regarding older adults, dependence, and stoma self-management. His clinical experience facilitated understanding of stoma-related terminology and clinical contexts but could also have directed attention toward clinically familiar aspects of self-management. The researcher also considered the possibility that gender might influence participants’ willingness to disclose sensitive experiences and that assumptions linking older age or greater dependence with poorer self-management could shape the interpretation of participants’ accounts. Open-ended questions and probing prompts were used to encourage participants to describe their experiences in their own terms rather than directing them toward predetermined interpretations. Regular team discussions were conducted to identify potential researcher bias, challenge alternative interpretations, and ensure that themes remained grounded in participants’ narratives rather than researchers’ preconceptions. To enhance transferability, detailed descriptions of the research setting, participant characteristics, and sampling criteria were provided to allow readers to assess the applicability of the findings to other contexts.

This qualitative study was reported according to the Consolidated Criteria for Reporting Qualitative Research (COREQ) checklist, which includes 32 items covering the research team and reflexivity, study design, and data analysis and reporting.

## 3. Results

Ultimately, 13 patients were selected for interviews and were labeled sequentially from “N1” to “N13.” Among them, 8 were male and 5 were female, ranging in age from 60 to 77 years. The general characteristics of the interviewees are presented in Table 1. Three major themes and eight sub-themes emerged from the interviews: (1) Decision-making dilemmas and uncertainty (Insufficient and poorly tailored decision-support resources; Decision-making challenges when processing complex self-management information); (2) Reconstructing daily life and identity after stoma formation (Disruption of body image and daily routines; Emotional struggle between rejection and acceptance; and Developing a new normal in daily living); and (3) Developing resilience through accumulated experience and self-management practice (Learning through trial and error and gaining confidence; Drawing on previous life experiences to cope with irreversible changes; and the Redefining life priorities after illness).

**Table 1:** Characteristics of participants (n = 13).

variable	Category	N(%)
Gender	Female	5(38.5)
	Male	8(61.5)
Age(years)	60-69	8(61.5)
	70-79	5(38.5)
Educational Background	University	1(7.7)
	High school	6(46.15)
	Junior high	6(46.15)
Marital Status	Married	10(76.9)
	single	0(0)
	widowed	1(7.7)
	divorced	2(15.4)
Duration of Ostomy	1-5years	11(84.6)
	≥6years	2(15.4)
Type of surgery	Colostomy	8(61.5)
	Ileostomy	5(38.5)
Primary diagnosis	Rectal cancer	4(30.8)
	Colon cancer	9(69.2)
Stoma-related complications	Yes	7(53.8)
	No	6(46.2)
Living arrangement	Living alone	3(23.1)
	Living with family	10(76.9)

Three themes reflecting different aspects of the long-term adaptation process were identified: (1) decision-making dilemmas and uncertainty, reflecting the difficulties encountered by patients after stoma formation; (2) reconstructing daily life and identity, describing how patients adjusted to bodily and lifestyle changes; and (3) developing resilience through accumulated experience and self-management practice, illustrating how patients gradually transformed challenges into personal resources.

### 3.1. Theme 1: Decision-making dilemmas and uncertainty

Older patients with permanent colostomies commonly face complex decision-making dilemmas during self-management. These dilemmas stem from both insufficient and poorly tailored decision support resources, and the increased cognitive load associated with aging patients, further exacerbating their decision-making stress amidst multiple pieces of information and changing situations.

#### 3.1.1. Insufficient and poorly tailored decision support resources.

Post-colostomy decisions involve complex and diverse issues, while the information support provided by medical institutions often focuses on key points, lacking systematic and comprehensive coverage. This makes it difficult for patients to make appropriate decisions when faced with uncertain situations.

*“Some advice I found online even contradicted what the doctor told me.” (N11)*

*“I feel like there is information everywhere, but nothing really fits my own situation.” (N12)*

*“I searched online for answers, but different sources said different things, which made me more confused.” (N03)*

#### 3.1.2. Decision-making challenges when processing complex self-management information.

Some older participants reported difficulties in understanding, retaining, and applying complex stoma care information. When faced with multiple or inconsistent sources of advice, these challenges increased their uncertainty and made decision-making more difficult.

*“The doctor gave me too much information at once. After I got home, I could hardly remember the details, and it became difficult for me to make a decision.” (N06)*

*“Sometimes I don’t fully understand the medical information, so I depend on my family to help me make decisions.” (N04)*

*“I forget things easily now, so I often hesitate when I need to make decisions.” (N05)*

### 3.2. Theme 2: Reconstructing daily life and identity after stoma formation

The adaptation process for older patients with permanent intestinal stomas is primarily characterized by an individual’s gradual transition—moving from initial feelings of loss and grief toward an acceptance of their new physical state, and ultimately achieving a sense of integration wherein they coexist with the stoma.

#### 3.2.1. Disruption of body image and daily routines.

The creation of a permanent stoma disrupts a patient’s perception of bodily integrity as well as the continuity of their daily life. Patients often feel a loss of control over their bodies, accompanied by alterations in daily routines, a diminished sense of autonomy, and the distinct sensation that their former way of life has fundamentally changed.

*“When I look at my body now, it feels different from before. It’s not just a physical change—I feel like I’m not the same person anymore.” (N03)*

*“I used to have control over my body, but now it feels unpredictable. Sometimes it leaks without warning, especially at night. I have to wake up and check, which affects my sleep.” (N12)*

*“Before the surgery, I could go out freely without thinking too much. Now, I have to plan everything in advance—what to eat, when to go out, and whether I need to bring extra supplies. My whole day revolves around the stoma.” (N08)*

#### 3.2.2. Emotional struggle between rejection and acceptance.

Throughout the entire adaptation process, patients remain in a state of psychological ambivalence: on one hand, they reject their altered body; on the other, they are compelled to pragmatically accept the reality that they must live with a stoma. Even after a period of long-term adaptation, many patients continue to exhibit a coexistence of emotional resistance and functional adaptation—a dynamic that reflects an enduring internal conflict.

*“It’s been a few years, and I’ve gotten used to managing it in daily life, but deep down, I still don’t like it.” (N06)*

*“Rationally, I know this saved my life, so I should accept it. But emotionally, I still find it hard. In certain situations—such as social interactions or moments of body exposure—this ambivalence becomes even more pronounced.” (N02)*

#### 3.2.3. Developing a new normal in daily living.

As the adaptation process progresses, some patients gradually shift from a stance of passive coping toward one of active acceptance, thereby integrating the stoma into the fabric of their daily lives. On this basis, patients gradually reconstruct a new life order by adjusting their lifestyles and cognitive patterns, thereby achieving an integrated state of coexistence with their stoma.

*“After a few years, it has become part of my daily life. I know when to change it, what to eat, and how to manage it.” (N07)*

*“I’ve adjusted my routine. Everything is arranged in a way that works with the stoma, so life feels more stable now.” (N03)*

*“I’ve learned to take things as they come. At my age, being able to live like this is already a good thing.” (N09)*

### 3.3. Theme 3:Developing resilience through accumulated experience and self-management practice

Older patients living with a permanent stoma demonstrate a gradual development of resilience alongside a reorientation of life meaning.

#### 3.3.1. Learning through trial and error and gaining confidence.

Managing a stoma was described as a gradual learning process shaped by repeated trial and error. Despite initial uncertainty and failure, older patients accumulated practical skills and confidence over time, leading to more stable and self-directed self-management.

*“In the beginning, I didn’t realize my body shape would change after surgery. The pouch didn’t fit well, and it leaked. Later, I had to keep adjusting how I applied it based on the changes around the stoma.” (N06)*

*“Before, I only dealt with it after something went wrong. Now I try to prevent it by preparing ahead.” (N02)*

*“When I started moving around more or doing some physical work, I noticed it was easier to leak. So now I prepare differently before going out or being active.” (N03)*

#### 3.3.2. Drawing on previous life experiences to cope with irreversible changes.

Older patients tended to reframe their condition through accumulated life experience, adopting a more accepting and pragmatic attitude.

*“I’ve been through a lot in life. Compared to that, having a stoma is something I can accept. At least I’m still here.” (N11)*

*“When you get older, you learn to take things as they come. There’s no point in fighting something you cannot change.” (N03)*

*“At my age, I don’t expect everything to be perfect anymore. Being able to live like this is already enough.” (N06)*

#### 3.3.3. Redefining life priorities after illness.

Living with a permanent stoma prompted patients to re-evaluate what matters most in life. Patients reported shifting their focus from physical limitations to maintaining daily functioning, family relationships, and emotional well-being.

*“I know my body is different now, but as long as I feel okay and not too troubled, that’s enough for me.” (N04)*

*“Before, I was busy with many things. Now I feel that being with my family is the most important.” (N11)*

## 4. Discussion

Our study employed in-depth interviews to explore the experiences of older patients living with a permanent stoma during adaptation, with a particular focus on participants’ perceived changes in self-management. The findings suggest that participants perceived self-management as an evolving experience shaped by ongoing challenges, adjustments, and accumulated experiences [9]. Three overarching themes were identified: “decision-making dilemmas and uncertainty,” “reconstructing daily life and identity after stoma formation,” and “developing resilience through accumulated experience and self-management practice.” Overall, participants described self-management as an evolving lived experience involving practical care, decision-making, adjustment to changes in daily life, and use of available support. Psychological adaptation, resilience, and self-efficacy appeared in participants’ accounts as related experiences or resources, but they represent conceptually distinct aspects of living with and managing a permanent stoma. Given the single retrospective interview, these concepts should therefore be understood as related but distinct aspects of participants’ experiences rather than as sequential stages or causal mechanisms.

Because self-management of a permanent stoma permeates daily life and entails continuous behavioral adjustments, older patients are frequently required to make decisions across multiple domains, including dietary choices, stoma care, and physical activity [19].Previous research has indicated that [20], Chinese patients with colorectal cancer commonly experience significant decision-making dilemmas when facing colostomy surgery; the findings of the present study align with these observations. Participants described encountering conflicting health information online, particularly when online advice differed from professional recommendations, which increased uncertainty about appropriate self-management strategies [21], such as selecting appropriate foods, managing stoma-related complications, and deciding when to seek professional support.However, existing decision-aid tools for colorectal cancer tend to focus primarily on disease screening and the selection of treatment regimens, with insufficient attention to supporting long-term self-management decisions [22,23]. In addition to the complexities of the external information environment, difficulties in processing and applying health information may further complicate decision-making among some older patients, particularly when information is complex or inconsistent [24]. Constrained by their health literacy, some older patients may struggle to understand and retain care-related information, which can undermine their capacity for independent decision-making and their confidence in doing so [25]. Participants also described relying on family members for some decisions and aspects of daily stoma management. This finding should be interpreted in the context of participants’ family circumstances, as most were married and lived with family members. In Chinese families, family members may provide practical and emotional support to older adults living with chronic conditions [26]. Rather than indicating inadequate self-management, such reliance may represent one way in which self-management is supported within the family context. These findings highlight the potential value of individualized and accessible decision support for older adults with permanent stomas. Structured information, clear guidance on evaluating conflicting health information, and opportunities for ongoing clarification may help address patients’ reported uncertainty [27]. When consistent with patients’ preferences, involving family members in health education and shared decision-making may also provide practical and emotional support [28]. Digital decision-aid tools may be a potential approach to providing such support, but their effectiveness for long-term stoma self-management requires further evaluation [23].

Previous studies have confirmed that living with a stoma can affect body image, daily routines, and emotional well-being [29]. Consistent with these findings, participants described psychological adaptation as an ongoing process involving changes in body image, identity, daily life, and emotional responses. Some participants described periods of rejection, distress, or uncertainty, whereas others described gradually incorporating the stoma into their daily lives and developing a sense of a “new normal” [30,31]; These accounts suggest that psychological adaptation and self-management are closely related but distinct dimensions of the adaptation experience [32]. Participants’ descriptions indicated that changes in emotional and identity-related experiences often occurred alongside changes in their approach to stoma care and daily activities; however, the present study cannot determine whether psychological adaptation led to changes in self-management or vice versa. From a theoretical perspective, this pattern is consistent with meaning-reconstruction perspectives, which emphasize how individuals make sense of illness-related changes and incorporate them into their ongoing lives [33,34]. These findings highlight the potential value of follow-up care that addresses both practical self-management needs and psychological concerns. Assessment of body image concerns, emotional distress, and difficulties incorporating the stoma into daily life may help clinicians identify patients who perceive greater challenges during adaptation [35,36].Psychological and supportive care can be tailored to patients’ expressed concerns and preferences rather than assuming a uniform adaptation trajectory.

Previous studies have often conceptualized resilience as an individual trait or a relatively stable personal resource that enables one to withstand adversity and maintain functioning [37]. However, our findings extend this perspective by suggesting that resilience among older patients with permanent stomas is not merely a pre-existing attribute but is gradually reconstructed through lived experience, continuous self-adjustment, and engagement in self-management practices. This interpretation aligns with contemporary resilience theories that emphasize resilience as a dynamic, developmental process shaped by person-environment interactions [38,39]. Participants frequently described accumulating practical experience, learning from unsuccessful attempts, and developing greater familiarity with stoma care. These accounts were often accompanied by descriptions of increased confidence and broader attention to maintaining daily activities, family relationships, and emotional well-being [40]. Rather than indicating a causal pathway from resilience to self-management, these findings suggest that resilience-related coping experiences and self-management practices may become intertwined in participants’ accounts over the course of adaptation. This interpretation is consistent with a strength-based perspective that recognizes older adults’ accumulated life experiences as potential resources for coping with bodily changes, reconstructing meaning, and managing everyday challenges [41,42]. In clinical practice, a strength-based approach may help patients recognize existing abilities and successful experiences in stoma management. Encouraging patients to reflect on situations they have successfully managed, identify personally meaningful coping strategies, and exchange experiences with peers may help reinforce confidence in their ability to manage everyday challenges [43,44]. Such approaches may be relevant to self-efficacy, but self-efficacy should be considered a related construct rather than a direct component or established causal mechanism of self-management in the present study.

## 5. Conclusion

This qualitative study explored the lived experiences and meanings of self-management among older adults with permanent stomas during adaptation. Participants’ retrospective accounts suggested perceived changes in self-management in relation to decisional uncertainty, psychological adjustment, accumulated experience, and resilience-related coping. These findings highlight the importance of individualized decision support, attention to psychological concerns, family involvement consistent with patients’ preferences, and strength-based care. Given the single retrospective interview, the findings reflect participants’ perceptions of change rather than direct observation of self-management development over time.

## 6. Limitations

This study has several limitations. First, participants were recruited from a single tertiary hospital, which may limit the transferability of the findings to other healthcare settings and cultural contexts. In addition, recruitment through a specialist stoma clinic may have introduced self-selection, as individuals who maintained contact with specialized stoma services or were more willing to engage in stoma-related care may have been more likely to participate. Second, participants varied in the duration since stoma formation. Although this variation was intentionally retained to capture diverse retrospective experiences, it may have contributed to differences in participants’ perceptions of adaptation and self-management. Third, because participants were asked to retrospectively describe experiences from stoma formation to the time of interview, recall bias cannot be excluded. Interviews were conducted in Mandarin Chinese and selected quotations were translated into English; although the translations were checked against the original transcripts, some linguistic or contextual nuances may not have been fully preserved. Finally, data were collected through a single retrospective interview at one time point. Therefore, the study captured participants’ perceived changes in self-management rather than directly observing changes across different stages of adaptation. Longitudinal qualitative studies using repeated interviews at multiple time points could provide richer insights into how self-management experiences develop and change over time.

## Supporting information

S1 Table Characteristics of participants (n = 13). This table presents the demographic and clinical characteristics of the participants included in this qualitative study. Participant characteristics are summarized according to gender, age, educational background, marital status, duration of ostomy, type of surgery, primary diagnosis, stoma-related complications, and living arrangement. Continuous variables are presented using descriptive statistics, while categorical variables are presented as frequencies and percentages. The table provides an overview of participant diversity and contextual information to support the interpretation and transferability of the study findings.
(DOCX)

S2 Table Completed COREQ (Consolidated criteria for Reporting Qualitative research) checklist. This table presents the completed COREQ checklist for this qualitative study. The checklist comprises 32 items organized across three domains: research team and reflexivity, study design, and data analysis and reporting. Each item is addressed to provide transparent and comprehensive documentation of the methodological and analytical processes employed in this study. The completed checklist supports the assessment of study rigor and credibility and facilitates readers’ evaluation of the quality and transparency of the research findings.
(DOCX)

S3 Table Example of the coding process. This table presents illustrative examples of the thematic analysis coding process employed in this qualitative study. The table demonstrates the progression from raw data extracts to the development of initial codes, sub-themes, and overarching themes. Each row includes a representative participant quotation, the corresponding initial code derived through open coding, the sub-theme identified through axial coding, and the final theme established through selective coding. The examples provided in this table offer transparency regarding the analytical trajectory and support the trustworthiness and rigor of the qualitative findings by enabling readers to trace the interpretive pathway from raw data to conceptual interpretation.
(DOCX)
