# Supplementary material for: Experiences of self-management behaviors during adaptation to permanent enterostomy among older patients: A qualitative study
Source: PLoS One. 2026 Oct 29;21(10):e0360643. doi: 10.1371/journal.pone.0360643 (PMC13718964; doi:10.1371/journal.pone.0360643)
Supplement: Supplementary file 1 [file pone.0360643.s005.pdf]

Experiences of Self-Management Behaviors During Adaptation to Permanent Enterostomy Among Elderly Patients: A Qualitative Study

Please use a different color to highlight the revisions.

Introduction

1. Too long → make it so focus and concise
2. The term "elderly patients" → replaced consistently with "older adults" or "older patients."
3. The terms "ostomy," "stoma," "enterostomy," and "intestinal ostomy" → should consistent
4. "ostomy surgery is a common intervention for CRC" → Not all patients with colorectal cancer require a stoma → clarify that stoma formation may be required in selected patients.
5. "a substitute for the anus" → anatomically simplistic and potentially inaccurate. Better → a segment of the intestine is brought through the abdominal wall to divert or facilitate fecal elimination.
6. National or regional data → to establish the relevance of the study in the country where the research was conducted.
7. The distinction between self-management, self-care, psychological adaptation, independence, and self-efficacy should be presented more concisely.
8. Add the strengths of older adults
9. Synthesize evidence from multiple studies rather than rely heavily on isolated findings.
10. The research gap needs to be narrowed down from general statements to a precise, well-justified problem.
11. The potentially novel contribution appears to concern how self-management develops through accumulated experience, repeated adjustment, decision-making, and problem-solving → stated more explicitly.
12. Objective: to understand a process of behavioural development → justify why descriptive phenomenology and Colaizzi's method were selected instead of grounded theory or a longitudinal qualitative design.
13. If the study is phenomenological, the objective should focus on the lived experience and meaning of developing self-management rather than attempting to construct a formal process model.
14. Provide a more critical synthesis of previous studies.

Methods

1. Descriptive phenomenology, interpretive phenomenology, or another phenomenological approach?
2. A single retrospective interview cannot directly demonstrate how self-management behaviours changed over time.
3. The wide variation in time since stoma formation → needs justification.
4. How purposive sampling was implemented, how participant diversity was achieved, and how many individuals were screened, declined, or were excluded.
5. The implementation of Colaizzi's method → insufficiently described.
6. Interviews were conducted in Mandarin, Mongolian, or another language →how English translations of participant quotations were produced and verified.
7. Who assessed saturation?
8. How thematic depth?
9. How the first author's clinical background, male gender, and assumptions regarding older adults, dependence, and stoma self-management may have influenced interviewing and interpretation.
10. Descriptions of data saturation, independent coding, and rigour are repeated across several subsections.

Discussion

1. Too long and repetitive.
2. Psychological adaptation, resilience, self-efficacy, and self-management are sometimes presented as overlapping concepts or causal mechanisms.
3. Some clinical recommendations are not sufficiently supported by the findings.

Conclusion

1. It should not imply that a dynamic process was directly observed.

Limitation

1. A small sample size is not inherently a limitation in phenomenological research.
2. Important limitations: single-centre recruitment, self-selection through a specialist stoma clinic, the wide variation in time since stoma formation, recall bias, and the use of a single retrospective interview.
