# Supplementary material for: Experiences of self-management behaviors during adaptation to permanent enterostomy among older patients: A qualitative study
Source: PLoS One. 2026 Oct 29;21(10):e0360643. doi: 10.1371/journal.pone.0360643 (PMC13718964; doi:10.1371/journal.pone.0360643)
Supplement: Supplementary file 2 [file pone.0360643.s001.docx]

**S1 Table 1. Characteristics of participants (n = 13)**

| variable | Category | N（%） |
| --- | --- | --- |
| Gender | Female | 5（38.5） |
|  | Male | 8（61.5） |
| Age（years） | 60-69 | 8(61.5) |
|  | 70-79 | 5(38.5) |
| Educational Background | University | 1（7.7） |
|  | High school | 6（46.15） |
|  | Junior high | 6（46.15） |
| Marital Status | Married | 10（76.9） |
|  | single | 0（0） |
|  | widowed | 1（7.7） |
|  | divorced | 2（15.4） |
| Duration of Ostomy | 1-5years | 11（84.6） |
|  | ≥6years | 2（15.4） |
| Type of surgery | Colostomy | 8（61.5） |
|  | Ileostomy | 5（38.5） |
| Primary diagnosis | Rectal cancer | 4（30.8） |
|  | Colon cancer | 9（69.2） |
| Stoma-related complications | Yes | 7（53.8） |
|  | No | 6（46.2） |
| Living arrangement | Living alone | 3（23.1） |
|  | Living with family | 10（76.9） |

**S1 Table 1. Characteristics of participants (Version with Corresponding Numbers)**

| Participant ID | Gender | Age | Educational Background | Marital Status | Duration of Ostomy | Ostomy Type | Primary diagnosis | Stoma-related complications | Living arrangement |
| --- | --- | --- | --- | --- | --- | --- | --- | --- | --- |
| N1 | Female | 62 | Junior high | Married | 4 years | Colostomy | Colon cancer | yes | Living with family |
| N2 | Male | 60 | University | Married | 2 years | Colostomy | Colon cancer | Yes | Living with family |
| N3 | Female | 70 | Junior high | Married | 2 years | Colostomy | Rectal cancer | No | Living with family |
| N4 | Male | 74 | High school | Married | 3 years | Colostomy | Colon cancer | Yes | Living with family |
| N5 | Male | 75 | Junior high | Married | 1 year | Ileostomy | Rectal cancer | No | Living with family |
| N6 | Female | 69 | High school | Married | 2 years | Colostomy | Colon cancer | No | Living with family |
| N7 | Male | 66 | High school | divorced | 5 years | Ileostomy | Colon cancer | Yes | Living alone |
| N8 | Male | 68 | Junior high | Married | 3 years | Colostomy | Colon cancer | Yes | Living with family |
| N9 | Male | 72 | High school | Married | 7 years | Ileostomy | Rectal cancer | No | Living with family |
| N10 | Female | 63 | Junior high | Married | 2 years | Colostomy | Rectal cancer | Yes | Living with family |
| N11 | Male | 77 | High school | widowers | 4 years | Ileostomy | Colon cancer | No | Living alone |
| N12 | Female | 63 | Junior high | Married | 6 years | Colostomy | Colon cancer | Yes | Living with family |
| N13 | Male | 65 | High school | divorced | 3 years | Ileostomy | Colon cancer | No | Living alone |
