# Supplementary material for: Experiences of self-management behaviors during adaptation to permanent enterostomy among older patients: A qualitative study
Source: PLoS One. 2026 Oct 29;21(10):e0360643. doi: 10.1371/journal.pone.0360643 (PMC13718964; doi:10.1371/journal.pone.0360643)
Supplement: Supplementary file 4 [file pone.0360643.s003.docx]

**S3 Table3. Example of the coding process**

| Data extract | Initial codes | Sub-theme | Theme |
| --- | --- | --- | --- |
| “Some advice I found online even contradicted what the doctor told me.” (P11) | Conflict between online information and professional medical advice | Insufficient and Poorly Tailored Decision Support Resources | Decision-Making Dilemmas and Uncertainty |
| “I searched online for answers, but different sources said different things, which made me more confused.” (P03) | Confusion caused by inconsistent information from multiple sources | Insufficient and Poorly Tailored Decision Support Resources | Decision-Making Dilemmas and Uncertainty |
| “The doctor gave me too much information at once. After I got home, I could hardly remember the details, and it became difficult for me to make a decision.” (P06) | Difficulty retaining and processing medical information during decision-making | Decision-Making Pressure Caused by Cognitive Load in Elderly Ostomy Patients | Decision-Making Dilemmas and Uncertainty |
| “Sometimes I don’t fully understand the medical information, so I depend on my family to help me make decisions.” (P04) | Dependence on family members due to low confidence in personal understanding | Decision-Making Pressure Caused by Cognitive Load in Elderly Ostomy Patients | Decision-Making Dilemmas and Uncertainty |
