# Supplementary material for: Experiences of self-management behaviors during adaptation to permanent enterostomy among older patients: A qualitative study
Source: PLoS One. 2026 Oct 29;21(10):e0360643. doi: 10.1371/journal.pone.0360643 (PMC13718964; doi:10.1371/journal.pone.0360643)
Supplement: Supplementary file 5 [file pone.0360643.s006.docx]

**Response to Reviewers**

**Manuscript ID:PONE-D-26-23135R1**

**Title:Experiences of Self-Management Behaviors During Adaptation to Permanent Enterostomy Among Elderly Patients: A Qualitative Study**

Dear Dr. Jamshed Akhtar and Reviewers,

We sincerely appreciate you and the reviewers for the careful evaluation of our manuscript entitled“Experiences of Self-Management Behaviors During Adaptation to Permanent Enterostomy Among Elderly Patients: A Qualitative Study” We are grateful for the valuable and constructive comments provided during the review process, which have greatly helped us improve the methodological rigor, transparency, and overall quality of our manuscript.

We have carefully considered all comments and suggestions and have revised the manuscript accordingly. A detailed point-by-point response to each comment is provided below. All revisions have been highlighted in the revised manuscript using Track Changes, and a clean version of the revised manuscript has also been submitted for your convenience.

We sincerely thank the editors and reviewers for their time, expertise, and thoughtful guidance. Their suggestions have substantially strengthened the clarity and rigor of this study, and we hope that the revised manuscript adequately addresses all concerns and meets the standards for publication in PLOS ONE.

Sincerely,

Fang Wu (Corresponding Author)

On behalf of all co-authors

**Section1. Response to Editor ( Page1-6 )**

**Section2. Response to Reviewer #1 ( Page 6-30)**

**Section 3. Response to Reviewer #2 ( Page 30-49)**

**Section 4. Response to Reviewer #3 ( Page 49)**

**Section 5. Response to Reviewer #4 ( Page 49-67)**

- **Section1. Response to Editor:**

We sincerely appreciate your careful evaluation of our manuscript and your valuable guidance throughout the review process. Your insightful comments and constructive recommendations have provided important direction for improving the clarity, methodological rigor, and overall quality of our study. We are grateful for the opportunity to revise our manuscript and for the rigorous standards maintained by the journal during the peer-review process. We have carefully considered all of your comments and have revised the manuscript accordingly. Below, we provide detailed responses to each of your comments, with corresponding revisions highlighted in the revised manuscript.

**★ 1. Manuscript Style Requirements / File Naming Format**

**Editor’s Comment:**

**Please ensure that your manuscript meets PLOS ONE's style requirements, including those for file naming.**

**➦Response**

Thank you for your reminder regarding PLOS ONE’s formatting and file-naming requirements. In the revised submission, we have carefully reviewed and adjusted all manuscript files to ensure compliance with the journal’s style guidelines. Specifically, we have checked and standardized the manuscript structure, formatting, reference style, and file organization according to the PLOS ONE requirements.

All submitted files have been appropriately renamed and organized, including the clean manuscript version, the revised manuscript with Track Changes, the point-by-point response letter, and the Supporting Information files. The Supporting Information files have also been labeled according to the journal’s requirements (e.g., S1 Table, S2 Table) and appropriately cited within the revised manuscript.

We appreciate your guidance in ensuring that our submission meets the journal’s formatting standards and have carefully implemented the required adjustments.

**★ 2. Data Availability Statement / Data Sharing Plan**

**Editor’s Comment:**

**When completing the data availability statement of the submission form, you indicated that you will make your data available on acceptance. We strongly recommend all authors decide on a data sharing plan before acceptance...**

**➦Response**

Thank you for your careful review and for highlighting the importance of establishing a clear data sharing plan prior to acceptance. We have carefully reviewed the PLOS ONE data availability requirements and have revised our Data Availability Statement accordingly to provide clearer information regarding data access and participant privacy protection.

In the revised submission, we have prepared and uploaded the minimal dataset underlying the findings of this study as Supporting Information. Before sharing, the dataset was carefully reviewed and fully anonymized by removing potentially identifying information to ensure the protection of participants’ privacy and confidentiality.

Because this study involved qualitative interviews with a relatively small group of elderly patients with permanent enterostomy, the full interview transcripts contain detailed personal experiences and contextual information that may pose a risk of participant identification. Furthermore, participants provided informed consent under the condition that their personal information and interview data would be protected. Therefore, the complete interview transcripts cannot be made publicly available. However, requests for additional information may be considered by the corresponding author upon reasonable request and appropriate ethical review.

These clarifications have been incorporated into the Data Availability Statement of the revised manuscript, and the anonymized minimal dataset has been provided as Supporting Information as requested.

**★ 3.Inclusion of Referenced Table**

**Editor’s Comment:**

**Please include a copy of Table xxxx which you refer to in your text on page 9.**

**➦Response**

Thank you for your careful review. We have checked the referenced table and ensured that the required table is included in the revised manuscript. The table has been placed in accordance with the journal’s formatting requirements.

**Revised text in the manuscript:**

page10:

**S1 Table 1. Characteristics of participants (n = 13)**

| variable | Category | N（%） |
| --- | --- | --- |
| Gender | Female | 5（38.5） |
|  | Male | 8（61.5） |
| Age（years） | Mean | 68 |
|  | Range | 60-77 |
| Educational Background | University | 1（7.7） |
|  | High school | 6（46.15） |
|  | Junior high | 6（46.15） |
| Marital Status | Married | 10（76.9） |
|  | single | 0（0） |
|  | widowed | 1（7.7） |
|  | divorced | 2（15.4） |
| Duration of Ostomy | 1-5years | 11（84.6） |
|  | ≥6years | 2（15.4） |
| Type of surgery | Colostomy | 8（61.5） |
|  | Ileostomy | 5（38.5） |
| Primary diagnosis | Rectal cancer | 4（30.8） |
|  | Colon cancer | 9（69.2） |
| Stoma-related complications | Yes | 7（53.8） |
|  | No | 6（46.2） |
| Living arrangement | Living alone | 3（23.1） |
|  | Living with family | 10（76.9） |

**★ 4.Supporting Information Files**

**Editor’s Comment:**

**We notice that your supplementary tables are included in the manuscript file. Please remove them and upload them with the file type "Supporting Information". Please ensure that each Supporting Information file has a legend listed in the manuscript after the references list.**

**➦Response**

Thank you for your careful review and for highlighting the need for clearer organization of the Supporting Information files. We have revised the manuscript and submission files accordingly to ensure compliance with PLOS ONE’s requirements for Supporting Information.

In the revised submission, all supplementary tables have been removed from the main manuscript file and uploaded separately as Supporting Information files. The corresponding legends for each Supporting Information file have also been added after the References section in the manuscript as requested. Specifically, S1 Table has been provided as “Characteristics of Participants (n = 13)” to describe the demographic and clinical characteristics of participants included in this qualitative study.

**Revised text in the manuscript:**

**Page31:**

**S1 Table 1. Characteristics of participants (n = 13)**

This table presents the demographic and clinical characteristics of the participants included in this qualitative study. Participant characteristics are summarized according to gender, age, educational background, marital status, duration of ostomy, type of surgery, primary diagnosis, stoma-related complications, and living arrangement. Continuous variables are presented using descriptive statistics, while categorical variables are presented as frequencies and percentages. The table provides an overview of participant diversity and contextual information to support the interpretation and transferability of the study findings.

**★ 5.Anonymization of Supporting Information Data**

**Editor’s Comment:**

**We note that there is identifying data in the Supporting Information file (S1 Table 1)...**

**Response:**

Thank you for your careful review and for highlighting the importance of protecting participant privacy in shared research data. We have carefully reviewed the Supporting Information file (S1 Table) and revised the dataset to ensure compliance with PLOS ONE’s data sharing policy and ethical requirements.

In the revised Supporting Information file, all directly identifying information, including investigator-related information and contact details, has been removed. We have also reviewed the dataset for potential indirect identifiers and ensured that only the minimum necessary information required to support the interpretation of the study findings is retained. A fully anonymized version of the dataset has been re-uploaded as Supporting Information.

These revisions ensure that the shared data protect participant confidentiality while maintaining the transparency and reproducibility of the study.

**★ 6.Reviewer-Suggested References**

**Editor’s Comment:**

**If the reviewer comments include a recommendation to cite specific previously published works, please review and evaluate these publications...**

**➦Response**

Thank you for your guidance regarding reviewer-suggested references. We have carefully evaluated all recommended citations and assessed their relevance to the conceptual framework, methodological approach, and research objectives of our study. References that were directly relevant and contributed meaningfully to the theoretical foundation or interpretation of our findings have been incorporated into the revised manuscript, while suggestions that were not sufficiently aligned with the scope of our study were not included, in accordance with the journal’s policy that such citations are optional unless specifically required by the editor. All newly added references have been appropriately cited and highlighted in the revised manuscript.

**★ 7.Additional Editor Comment: Title, Adaptation Process, and Data Saturation**

**Editor’s Comment:**

**The subject is important and representing a particular geographic region is also appreciated. However, the depth of the subject as mentioned in the title, like adaptation and long term are not adequately addressed in this qualitative study with sample size based upon different sub set of patients, not appropriately reflect that. How data saturation is arrived with diverse experiences is not clear.**

**➦Response**

We sincerely appreciate the editor’s insightful comment. We agree that the original wording of the title may have implied a stronger longitudinal perspective than could be fully supported by a cross-sectional qualitative design. Therefore, we revised the title to more accurately reflect the scope of our study, changing it from “Experiences of Self-Management Behaviors in the Long-Term Adaptation Process of Elderly Patients with Permanent Enterostomy: A Qualitative Study” to “**Experiences of Self-Management Behaviors During Adaptation to Permanent Enterostomy Among Elderly Patients: A Qualitative Study.**”

In addition, we have clarified in the revised manuscript that participants’ descriptions represented retrospective accounts of their adaptation experiences rather than direct observation of changes over time. This limitation has also been acknowledged in the revised **Limitations** section.

Regarding data saturation, we have further clarified the process used to determine saturation in the **Methods** section. During the iterative data collection and analysis process, interviews were continued until no new codes, categories, or conceptual insights emerged from subsequent interviews, supporting the adequacy of the final sample size.

In the manuscript, this revised content is presented as follows:

Change the title to:Experiences of Self-Management Behaviors During Adaptation to Permanent Enterostomy Among Elderly Patients: A Qualitative Study

**6 Limitations**

*This study has several limitations. First, the relatively small sample size and recruitment from a single tertiary hospital may limit the transferability of the findings to other settings with different cultural and healthcare contexts. Second, variations in participant characteristics, such as gender distribution and postoperative duration, may have influenced the results, and some experiences may be underrepresented. Third, as interviews were conducted in Chinese and later translated into English, subtle nuances in meaning may have been affected despite careful translation procedures; additionally, recall bias may be present as participants reflected on past experiences. Finally, although participants were encouraged to retrospectively describe changes in their self-management behaviors from stoma formation to the present, data were collected through a single interview at one time point. Therefore, the findings represent participants’ retrospective accounts of their adaptation experiences rather than direct observation of changes across different stages of adaptation. Future longitudinal qualitative studies with repeated interviews at multiple time points are needed to further explore the dynamic changes in self-management behaviors and adaptation experiences among elderly patients with permanent enterostomy.*

**2.4 Data collection**

*Participants were recruited through recruitment posters posted at an ostomy outpatient clinic using purposive sampling. This approach was chosen to obtain rich and diverse accounts of self-management experiences among elderly patients living with permanent enterostomy, with consideration of variations in stoma duration and individual adaptation experiences. All interviews were conducted by the first author, a male nursing graduate student with clinical experience in gastrointestinal surgery and training in qualitative research methods, including qualitative interviewing and data analysis. The interviewer had no prior therapeutic relationship with the participants, and participation or refusal did not affect their medical care. Before each interview, the researcher explained the study purpose, procedures, voluntary participation, and confidentiality principles, and written informed consent was obtained before audio recording. Face-to-face semi-structured interviews were conducted in a private demonstration room at the ostomy outpatient clinic using an interview guide. Open-ended questions and probing techniques were used to encourage participants to describe their experiences in depth, and field notes were recorded to capture contextual information and non-verbal expressions. Each interview lasted approximately 20–40 minutes, depending on participants’ willingness and the richness of their experiences. Although each participant was interviewed once, the semi-structured interview approach allowed participants to retrospectively describe their experiences from stoma formation to the present, including changes in self-management behaviors, challenges encountered, and adaptation processes.and all participants were interviewed once. Audio recordings were transcribed verbatim and checked for accuracy after each interview.* ***Data collection continued until saturation was achieved; after the 10th interview, no substantially new codes emerged, and three additional interviews were conducted for confirmation. After the 13th interview, no new codes or themes were identified, and data collection was terminated.***

**★ 8. Deadline and Required Files for Resubmission**

**Response to Reviewers**

**Revised Manuscript with Track Changes**

**Clean Manuscript without Track Changes**

- **Section2. Response to Reviewer #1:**

We would like to express our sincere gratitude to you for your thorough and insightful review of our manuscript. Your comments were highly professional, thoughtful, and constructive, providing us with valuable guidance for improving the methodological rigor, conceptual clarity, and overall quality of our qualitative study. Your careful evaluation helped us identify important areas requiring further clarification, including the rationale for the research methodology, qualitative reporting procedures, conceptualization of self-management, interpretation of themes, and practical implications of our findings.

Your suggestions have provided a valuable opportunity for us to reflect more deeply on the design, analysis, and presentation of this study. We greatly appreciate your expertise and commitment to advancing the quality of qualitative research. Through your guidance, we have further strengthened the transparency, coherence, and clinical relevance of our manuscript, with the aim of providing a more meaningful understanding of self-management experiences among elderly patients living with permanent enterostomy.

Below, we provide detailed, point-by-point responses to each of your comments. **All revisions have been carefully incorporated into the “Revised Manuscript with Track Changes,” and corresponding textual changes are clearly indicated for your convenience.**

Once again, we sincerely thank you for your valuable time, constructive feedback, and professional guidance. Your insightful comments have significantly contributed to strengthening this manuscript, and we believe that the revised version has been substantially improved as a result of your thoughtful suggestions.

Major Concerns：

★ **1.Reporting According to COREQ Checklist**

**Reviewer’s Comment:**

**The manuscript should explicitly state that it follows the Consolidated Criteria for Reporting Qualitative Research (COREQ) checklist. A completed COREQ checklist should be submitted as supplementary material.**

**➦Response**

Thank you for highlighting the importance of transparent reporting in qualitative research. We have revised the manuscript to explicitly state that this study was reported according to the Consolidated Criteria for Reporting Qualitative Research (COREQ) checklist. In addition, a completed COREQ checklist has been prepared and submitted as Supporting Information (S2 Table) to provide a comprehensive description of the methodological and reporting procedures.

In the manuscript, this revised content is presented as follows:

2.7 Rigour and reflexivity

*This qualitative study was reported according to the Consolidated Criteria for Reporting Qualitative Research (COREQ) checklist, which includes 32 items covering the research team and reflexivity, study design, and data analysis and reporting. The completed COREQ checklist is provided as Supporting Information (S2 Table).*

**★ 2.Rationale for Selecting a Phenomenological Approach**

**Reviewer’s Comment:**

**The authors state that a phenomenological approach was used. Please clarify why this methodology was chosen over grounded theory or thematic analysis.**

**➦Response**

Thank you for this insightful comment. We have further clarified the rationale for selecting a phenomenological approach in the revised manuscript. The purpose of this study was to explore and understand the lived experiences and meanings associated with self-management behaviors among elderly patients with permanent enterostomy during adaptation, rather than to generate a formal theoretical model or merely identify patterns within qualitative data.

Therefore, a descriptive phenomenological approach was considered appropriate for capturing participants’ subjective experiences and perceptions. Grounded theory was not selected because the aim was not to develop a new theory, and thematic analysis was not adopted because our primary focus was on the depth and meaning of individual lived experiences.

The revised text is now presented in the manuscript as follows:

**2.1 Study design**

*This study employs a phenomenological research approach to explore the experiences and needs associated with the self-management process from the patient's perspective. This approach was selected because it focuses on understanding individuals’ subjective experiences and the meanings they attribute to a particular phenomenon, which was consistent with the aim of this study to explore patients’ experiences of self-management during long-term adaptation after enterostomy. Through semi-structured in-depth interviews, the study investigates the self-management behaviors of elderly patients with permanent intestinal stomas during their long-term adaptation process. The research was conducted from March 25 to May 5, 2026, within the Department of Gastrointestinal Surgery and the Stoma Clinic of a tertiary hospital in the Inner Mongolia Autonomous Region, China. The hospital provides specialized care for patients with colorectal diseases, and the Stoma Clinic offers postoperative follow-up, stoma care education, and complication management services for patients with enterostomies. The sample size was determined based on the principle of data saturation; specifically, when interviews ceased to yield new themes or information, a small number of additional participants were recruited to verify data saturation, and data collection was terminated once the sufficiency of information was confirmed16.*

★ **3.Interviewer Background, Reflexivity, and Researcher Bias**

**Reviewer’s Comment:**

**Please report the interviewer’s previous experience in qualitative research, relationship between interviewer and participants, whether interviewer assumptions were documented through reflexive journaling, and how researcher bias was minimized throughout coding and interpretation.**

**➦Response**

Thank you for raising this important issue regarding qualitative rigor and reflexivity. We have expanded the Methods section to provide additional details regarding the interviewer’s background, relationship with participants, reflexive practice, and strategies used to minimize researcher bias.

In the revised manuscript, we clarify the interviewer’s qualitative research experience and confirm that there was no prior personal relationship between the interviewer and participants. Reflective notes were maintained during the research process to document methodological decisions, personal reflections, and potential assumptions. Furthermore, researcher bias was minimized through systematic coding procedures, repeated discussions among the research team, and consensus-based refinement of themes.

The revised content now appears in the manuscript as follows:

**2.4 Data collection**

*Participants were recruited through recruitment posters posted at an ostomy outpatient clinic using purposive sampling. This approach was chosen to obtain rich and diverse accounts of self-management experiences among elderly patients living with permanent enterostomy, with consideration of variations in stoma duration and individual adaptation experiences. All interviews were conducted by the first author, a male nursing graduate student with clinical experience in gastrointestinal surgery and training in qualitative research methods, including qualitative interviewing and data analysis. The interviewer had no prior therapeutic relationship with the participants, and participation or refusal did not affect their medical care. Before each interview, the researcher explained the study purpose, procedures, voluntary participation, and confidentiality principles, and written informed consent was obtained before audio recording. Face-to-face semi-structured interviews were conducted in a private demonstration room at the ostomy outpatient clinic using an interview guide. Open-ended questions and probing techniques were used to encourage participants to describe their experiences in depth, and field notes were recorded to capture contextual information and non-verbal expressions. Each interview lasted approximately 20–40 minutes, depending on participants’ willingness and the richness of their experiences. Although each participant was interviewed once, the semi-structured interview approach allowed participants to retrospectively describe their experiences from stoma formation to the present, including changes in self-management behaviors, challenges encountered, and adaptation processes.and all participants were interviewed once. Audio recordings were transcribed verbatim and checked for accuracy after each interview. Data collection continued until saturation was achieved; after the 10th interview, no substantially new codes emerged, and three additional interviews were conducted for confirmation. After the 13th interview, no new codes or themes were identified, and data collection was terminated.*

**2.7 Rigour and reflexivity**

*To enhance the methodological rigor of this qualitative study, the four criteria proposed by Lincoln and Guba, including credibility, dependability, confirmability, and transferability, were considered throughout the research process. Credibility was enhanced through purposive sampling to recruit participants with diverse experiences of living with a permanent enterostomy. Two researchers independently coded the interview transcripts, and coding results were compared and discussed within the research team until consensus was reached. To ensure dependability, detailed records of participant recruitment, data collection, and data analysis procedures were maintained to establish an audit trail. All transcripts were repeatedly reviewed and compared with the original audio recordings to ensure accuracy. To enhance confirmability, reflexive practices were incorporated throughout data analysis. The interviewer maintained reflexive notes documenting personal assumptions, reflections, and methodological decisions during data collection and interpretation. To minimize researcher bias during coding and interpretation, two researchers independently coded the interview transcripts, and coding results were compared and discussed within the research team until consensus was reached. Regular team discussions were conducted to identify potential biases and ensure that themes were grounded in participants’ narratives rather than researchers’ preconceptions. To enhance transferability, detailed descriptions of the research setting, participant characteristics, and sampling criteria were provided to allow readers to assess the applicability of the findings to other contexts. Data collection continued until data saturation was achieved. Data saturation was assessed throughout the data collection and analysis process. After the 10th interview, no substantially new codes were identified from the interview transcripts. However, to confirm the stability and comprehensiveness of the emerging findings, three additional interviews were conducted. After the 13th interview, no new codes or themes emerged, and the research team determined that sufficient information had been obtained to answer the research question. Therefore, participant recruitment and data collection were terminated.*

★ **4.Expanded Participant Characteristics**

**Reviewer’s Comment:**

**The demographic table is limited.**

**➦Response**

Thank you for this valuable suggestion. We agree that providing more comprehensive participant characteristics can improve the transparency and transferability of qualitative research findings. Therefore, we have expanded the participant characteristics table to include additional demographic and clinical information.

➦**Comparison before and after modification**

**Before modification:**

**S1 Table 1. Characteristics of participants (n = 13)**

| Participant ID | Gender | Age | Educational Background | Marital Status | Duration of Ostomy | Ostomy Type |
| --- | --- | --- | --- | --- | --- | --- |
| N1 | Female | 62 | Junior high | Married | 4 years | Colostomy |
| N2 | Male | 60 | University | Married | 2 years | Colostomy |
| N3 | Female | 70 | Junior high | Married | 2 years | Colostomy |
| N4 | Male | 74 | High school | Married | 3 years | Colostomy |
| N5 | Male | 75 | Junior high | Married | 1 year | Ileostomy |
| N6 | Female | 69 | High school | Married | 2 years | Colostomy |
| N7 | Male | 66 | High school | divorced | 5 years | Ileostomy |
| N8 | Male | 68 | Junior high | Married | 3 years | Colostomy |
| N9 | Male | 72 | High school | Married | 7 years | Ileostomy |
| N10 | Female | 63 | Junior high | Married | 2 years | Colostomy |
| N11 | Male | 77 | High school | widowers | 4 years | Ileostomy |
| N12 | Female | 63 | Junior high | Married | 6 years | Colostomy |
| N13 | Male | 65 | High school | divorced | 3 years | Ileostomy |

**Changed:**

***S1 Table 1****. Characteristics of participants (n = 13)*

| *variable* | *Category* | *N（%）* |
| --- | --- | --- |
| *Gender* | *Female* | *5（38.5）* |
|  | *Male* | *8（61.5）* |
| *Age（years）* | *60-69* | *8(61.5)* |
|  | *70-79* | *5(38.5)* |
| *Educational Background* | *University* | *1（7.7）* |
|  | *High school* | *6（46.15）* |
|  | *Junior high* | *6（46.15）* |
| *Marital Status* | *Married* | *10（76.9）* |
|  | *single* | *0（0）* |
|  | *widowed* | *1（7.7）* |
|  | *divorced* | *2（15.4）* |
| *Duration of Ostomy* | *1-5years* | *11（84.6）* |
|  | *≥6years* | *2（15.4）* |
| *Type of surgery* | *Colostomy* | *8（61.5）* |
|  | *Ileostomy* | *5（38.5）* |
| *Primary diagnosis* | *Rectal cancer* | *4（30.8）* |
|  | *Colon cancer* | *9（69.2）* |
| *Stoma-related complications* | *Yes* | *7（53.8）* |
|  | *No* | *6（46.2）* |
| *Living arrangement* | *Living alone* | *3（23.1）* |
|  | *Living with family* | *10（76.9）* |

★ **5.Additional Variables to Improve Transferability**

**Reviewer’s Comment:**

**Additional variables would improve transferability, including primary diagnosis, time since cancer diagnosis, type of surgery, presence of complications, educational level categorized more clearly, and living alone versus living with family.**

**➦Response**

Thank you for this valuable suggestion. We agree that providing detailed demographic and clinical characteristics can enhance the transparency and transferability of qualitative research findings. Accordingly, we have expanded the participant characteristics table to include additional variables, including primary diagnosis, type of surgery, stoma-related complications, educational background, and living arrangement.

Regarding the variable of time since cancer diagnosis, this information was not systematically collected during the original data collection process. In our clinical setting, many participants received their initial cancer diagnosis at other hospitals and were subsequently referred to our hospital for surgical treatment and follow-up care. Therefore, complete and reliable information regarding the exact time of initial cancer diagnosis was not available for all participants. Given that the focus of this study was on patients’ adaptation and self-management experiences after permanent enterostomy, we retained variables that were directly relevant to the stoma-related adaptation process, such as duration of ostomy, type of surgery, and stoma-related complications.

We have acknowledged this limitation and ensured that the available demographic and clinical characteristics are presented comprehensively in the revised Supporting Information **(S1 Table).**

★ **6.Development of the Interview Guide and Pilot Testing**

**Reviewer’s Comment:**

**The interview guide contains only broad questions. Please indicate which theoretical framework informed the interview questions and whether pilot interviews resulted in modifications.**

**➦Response**

Thank you for this important suggestion. We have further clarified the development process of the interview guide in the revised manuscript. The interview questions were developed based on previous literature regarding stoma self-management, adaptation experiences, and chronic illness transitions, while considering the objectives and phenomenological perspective of this study.

In addition, pilot interviews were conducted before formal data collection. Based on these interviews, the wording and sequence of questions were refined to improve clarity and facilitate deeper exploration of participants’ experiences.

➦**Comparison before and after modification**

**Before modification**:2.5 Develop an Interview Outline

Based on an in-depth literature review and aligned with the objectives of this study, the researchers independently drafted an initial version of the interview guide. Following consultations with experts in stoma care and psychology—during which appropriate adjustments were made to the guide—and incorporating feedback obtained from pilot interviews conducted with two elderly patients with permanent intestinal stomas, the final version of the interview guide was established. The interview guide is as follows:

**Changed:** 2.5 Develop an Interview Outline

*The interview guide was developed based on an in-depth literature review and informed by the self-management framework[8], which emphasizes the acquisition of self-management skills, problem-solving, support utilization, and adaptation among individuals living with chronic conditions. The questions were designed to explore elderly patients’ experiences, challenges, behavioral changes, and support needs related to stoma self-management during adaptation to permanent enterostomy. The preliminary interview guide was reviewed by experts in stoma care and psychology, and appropriate modifications were made based on their feedback. Two pilot interviews were then conducted with elderly patients with permanent enterostomies who met the inclusion criteria but were not included in the final sample. Based on feedback from the pilot interviews, three modifications were made: (1) temporal anchoring was added to Question 1 to facilitate participants’ recall of the learning process; (2) probing prompts (e.g., “Can you describe a specific situation?”) were incorporated to encourage detailed narratives; and (3) technical terms were replaced with plain language to improve comprehensibility among elderly participants with varying health literacy levels. Following these refinements, the final version of the interview guide was established as follows:*

★ **7.Participant Numbering Consistency**

**Reviewer’s Comment:**

**Participant numbering inconsistency.**

**➦Response**

Thank you for identifying this issue. We carefully reviewed all participant identifiers throughout the manuscript and corrected the numbering to ensure consistency across the main text, tables, and Supporting Information.

**★ 8. Strengthening the Novelty and Interpretation of Findings**

**Reviewer’s Comment:**

**The Discussion largely summarizes previous literature. The authors should better emphasize what is genuinely novel about elderly patients, how their findings differ from previous qualitative studies, and why resilience emerged as a dominant theme.**

**➦Response**

Thank you for this insightful comment. We have revised the Discussion section to better emphasize the unique contribution of our findings. Specifically, we have highlighted how older adults with permanent enterostomy experience adaptation differently due to aging-related changes, accumulated life experiences, and the irreversible nature of stoma-related changes.

We have also expanded the interpretation of resilience, emphasizing that resilience was not simply an individual psychological trait but developed through repeated self-adjustment, accumulated life experiences, and reconstruction of daily life and identity.

In the manuscript, this revised section now appears as follows:

**4 Discussion**

Our study employed in-depth interviews to explore the experiences of elderly patients living with a permanent stoma during long-term adaptation, with a particular focus on the development of self-management behaviors. The findings suggest that self-management in this population is not a static skill but a dynamic process shaped by ongoing challenges and adjustments[8]. *Three overarching themes were identified: “decision-making dilemmas and uncertainty,” “reconstructing daily life and identity after stoma formation,” and “developing resilience through accumulated experience and self-management practice.” These findings indicate that elderly patients’ self-management experiences are shaped by continuous interactions among stoma-related challenges, personal adaptation, and available support. Reflecting on their experiences after stoma formation, participants described a transition from initial uncertainty and disruption toward greater confidence in managing daily life and reconstructing personal meaning.* *Our findings provide a process-oriented understanding of how elderly patients gradually transform uncertainty and disruption into adaptive self-management through continuous learning, adjustment, and the use of available resources.*

*Our findings indicate that elderly patients living with a permanent stoma gradually developed psychological resilience through continuous self-adjustment and lived experience. The prominence of resilience in this study may be related to the cumulative life experiences of elderly adults[36]. Throughout their lives, elderly individuals may have encountered previous transitions, losses, and health-related challenges, which can serve as psychological resources for coping with the irreversible changes associated with permanent enterostomy[37]. Previous studies have often conceptualized resilience as an individual trait or a relatively stable personal resource that enables one to withstand adversity and maintain functioning[38]. However, our findings extend this perspective by suggesting that resilience among elderly patients with permanent stomas is not merely a pre-existing attribute but is gradually reconstructed through lived experience, continuous self-adjustment, and engagement in self-management practices. This interpretation aligns with contemporary resilience theories that emphasize resilience as a dynamic, developmental process shaped by person–environment interactions[39,40]. In this study, patients did not achieve effective self-management immediately after stoma formation. Instead, they gradually accumulated practical skills and confidence through repeated attempts, problem-solving, and adjustment in daily care routines. As patients became more familiar with stoma management, they shifted their attention from physical limitations toward maintaining daily activities, family relationships, and emotional well-being[41]. This process suggests that resilience functioned not only as a psychological coping resource but also as a facilitating factor supporting sustained self-management behaviors[42].While previous studies have largely focused on the psychological burden and negative experiences of stoma care[43], our findings highlight the adaptive capabilities of elderly patients and suggest that their accumulated life experiences may serve as valuable resources for reconstructing meaning, accepting bodily changes, and developing long-term self-management strategies[44]. These findings imply that interventions for elderly patients with permanent enterostomy should not focus solely on correcting deficits but should also incorporate a strength-based approach that recognizes patients’ existing abilities and life experiences. In clinical practice, healthcare professionals may encourage patients to reflect on successful self-management experiences, identify personal coping strategies, and participate in peer-support activities to enhance self-efficacy and promote sustained self-management behaviors[45,46].*

**★ 9. Language Editing and Terminology Standardization**

**Reviewer’s Comment:**

**The manuscript requires professional English editing and standardization of terminology.**

**➦Response**

Thank you for this helpful suggestion. We have carefully revised the manuscript to improve language clarity, academic expression, and consistency of terminology throughout the text. In addition, the manuscript has undergone professional English language editing, with particular attention given to the Introduction and Discussion sections, where sentence structure, logical flow, and scholarly expression were further refined.

We have also reviewed and standardized key terminology related to self-management, adaptation, resilience, and stoma-related experiences to ensure conceptual consistency throughout the manuscript.

➦**Comparison before and after modification**

**Before modification**:1 Introduction

As one of the most common malignancies, colorectal cancer (CRC) poses a severe threat to human health due to its high incidence and mortality rates.According to the Global Cancer Statistics released by the International Agency for Research on Cancer (IARC) in 20221, newly diagnosed cases of CRC accounted for 9.6% of all cancer cases, while its mortality rate constituted 9.3% of all cancer-related deaths. Ostomy surgery is a common surgical intervention for CRC; it involves surgically creating an opening in the abdominal wall through which a segment of the intestine is brought out to serve as a substitute for the anus2.This procedure effectively alleviates patient symptoms, prevents disease progression, and extends survival3.However, due to factors such as disease progression or the specific surgical approach employed, some patients require a stoma for an extended period—or even for the remainder of their lives—resulting in a permanent intestinal ostomy.

Unlike temporary stomas, a permanent intestinal stoma implies that patients must contend—over an extended period—with issues such as altered physiological functions and the restructuring of their lifestyle4.Postoperatively, patients are required not only to cope with stoma-related complications and symptoms of discomfort but also to constantly adapt their behavioral patterns in daily life; consequently, their quality of life is generally affected to varying degrees5.Therefore, enhancing the self-management capabilities of patients with permanent intestinal stomas is of significant importance.

Following ostomy surgery, self-management permeates every aspect of a patient's daily life, encompassing dietary adjustments, stoma care, and participation in social activities. This process relies not only on relevant knowledge and skills but also, more importantly, involves the individual's continuous behavioral adjustments and decision-making across various contexts6,7.However, with the widespread adoption of Enhanced Recovery After Surgery (ERAS) protocols, hospital stays have become progressively shorter; consequently, some patients have not yet fully developed self-management capabilities by the time of discharge. Furthermore, during the early postoperative period, most patients struggle to promptly adapt to the changes in body image resulting from the presence of a stoma, and their awareness regarding the proactive assumption of self-management responsibilities remains relatively insufficient8.Among elderly patients with intestinal stomas, the aforementioned issues are particularly pronounced. As they age, these patients often experience characteristics such as cognitive decline, diminished reaction times, and reduced learning capacity, which increase the difficulty of mastering the knowledge and skills required for stoma care9.Relevant studies indicate that the overall level of self-management among elderly stoma patients remains low, with only approximately 50.4% possessing an adequate level of relevant knowledge10.Furthermore, the procedures involved in stoma care are inherently complex and technically demanding; consequently, elderly patients often struggle to successfully navigate the transition from a role of "dependent care" to one of "independent self-care" within a short timeframe11.

Although existing studies have explored the self-care experiences and needs of patients with stomas, most have focused primarily on describing the associated problems, lacking an in-depth analysis of how self-management behaviors are formed and evolve during the long-term adaptation process—particularly within the demographic of elderly patients with permanent intestinal stomas. Consequently, this study employs a qualitative research approach, utilizing in-depth interviews with elderly patients with permanent intestinal stomas to investigate the formation and evolution of their self-management behaviors during the long-term adaptation process. The aim is to provide a theoretical foundation and practical reference for the development of future intervention strategies.

4 Discussion

Our study employed in-depth interviews to explore the experiences of elderly patients living with a permanent stoma during long-term adaptation, with a particular focus on the development of self-management behaviors. The findings suggest that self-management in this population is not a static skill but a dynamic process shaped by ongoing challenges and adjustments14. Three overarching themes emerged: “decision-making dilemmas and uncertainty,” “psychological reconstruction during adaptation,” and “resilience narratives and meaning-making.” Together, these findings highlight that elderly patients gradually move from uncertainty and disrupted life experiences toward more stable self-management and redefined life meaning.

Since the self-management of a permanent stoma permeates a patient's daily	life and entails continuous behavioral adjustments, elderly patients are frequently required to make	decisions across various domains, including dietary choices, stoma care, and participation in physical activities15.Previous studies have indicated that16，Chinese patients with colorectal cancer commonly experience significant decision-making dilemmas when facing colostomy surgery; the findings of the present study align with these observations. This study revealed that during the process of seeking information online, patients often encounter cognitive conflicts stemming from the diversity of information sources and the inconsistency of their content. This issue is particularly exacerbated when online information contradicts the advice provided by healthcare professionals, thereby intensifying elderly patients' confusion and sense of uncertainty regarding decision-making.However, existing decision-aid tools for colorectal cancer tend to focus primarily on disease screening and the selection of treatment regimens, while paying insufficient attention to providing decision support within the context of long-term self-management following stoma surgery17,18.In addition to the complexities of the external information environment, age-related cognitive decline constitutes another significant factor that compounds decision-making difficulties for elderly patients. Constrained by their levels of health literacy, elderly patients often struggle to comprehend and retain care-related information, which, to a certain extent, undermines their capacity for independent decision-making as well as their confidence in doing so19.Given these multifaceted factors—and against the backdrop of rapidly advancing digital health technologies—it is imperative to develop scientifically sound and standardized digital decision-aid tools. By providing structured, simplified, and personalized information, such tools can effectively enhance patients' decision-making capabilities within complex circumstances. Furthermore, this study observed that some patients exhibit a degree of reliance on family members during the decision-making process, underscoring the critical supportive role that the family plays in patients' decisions. Consequently—and contingent upon the patient's informed consent—integrating family members into the shared decision-making and health education processes can contribute to improved decision quality and enhanced health outcomes for patients20.

Previous studies have confirmed that patients with stomas frequently face alterations in body image and psychological experiences akin to a grief response21. Building upon this foundation, the present study further reveals that this process of psychological adaptation is characterized by significant long-term and non-linear features; specifically, even a considerable time after surgery, patients may continue to experience recurrent emotional fluctuations and conflicts regarding their sense of identity. From a theoretical perspective, this process resonates with theoretical frameworks such as "meaning reconstruction," suggesting that patients do not merely revert to their pre-operative state but, rather, through a continuous process of adjustment and adaptation, gradually construct a "new normal" that incorporates the presence of the stoma22. Based on these findings, clinical practice should prioritize both the continuity of psychological support and the recognition of phase-specific differences in patient needs23. During the early post-operative period, the primary focus should be on addressing patients' distress regarding body image and their sense of loss of control, providing targeted psychological counseling. In the long-term follow-up phase, it is essential to identify patients who remain in a state of psychological conflict and, through interventions such as narrative therapy and peer support groups, facilitate their integration of meaning to enhance their overall level of adaptation24,25.

Our findings indicate that elderly patients living with a permanent stoma gradually cultivate psychological resilience through continuous self-adjustment and lived experience. Rather than achieving self-management instantaneously, these patients progressively accumulate skills and build confidence through a process of repeated trial and error embedded within their daily care routines. Concurrently, the wealth of life experience accumulated during their later years appears to foster a more pragmatic and accepting attitude toward physical changes26. Patients often shift their focus away from the limitations of their physical functioning, placing greater emphasis instead on maintaining daily living activities, family relationships, and emotional well-being27. While previous studies have largely focused on the psychological burden and negative experiences associated with stoma care28, our findings specifically highlight the adaptive capabilities possessed by elderly patients. Specifically, their accumulated life experience was identified as a crucial coping resource, providing robust support for their acceptance of reality and their process of self-adjustment29. These findings suggest that interventions for this patient population should not be confined solely to addressing their problems and deficits; rather, they should also acknowledge and build upon their existing capabilities and invaluable experiences. By guiding patients to reflect on their positive experiences during the adaptation process, clinicians can help enhance their self-confidence and sense of self-efficacy, thereby fostering the continuous improvement of their long-term self-management skills30.

**Changed:**1 Introduction

*Colorectal cancer (CRC) is one of the most common malignancies and poses a s ignificant threat to human health because of its high incidence and mortality rates. According to Global Cancer Statistics 2022 released by the International Agency for Research on Cancer (IARC)[1],* newly diagnosed cases of CRC accounted for 9.6% of all cancer cases, *and CRC-related deaths* constituted 9.3% of all cancer-related deaths. *Ostomy surgery, a common intervention for CRC, involves creating an opening in the abdominal* wall through which a segment of the intestine is *exteriorized* to serve as a substitute for the anus[2]. This procedure effectively alleviates symptoms, prevents disease progression, and extends survival[3].However, *owing to* factors such as disease advancement or the specific surgical approach employed, some patients require a stoma for an extended period—or even for *life*—resulting in a permanent intestinal ostomy.

Unlike temporary stomas, a permanent intestinal stoma implies that patients must contend *over the long term* with issues such as altered physiological function and restructuring of their lifestyle[4]. Postoperatively, patients *need not only* to cope with stoma-related complications and discomfort but also to *continuously adapt their daily behaviors;* consequently, their quality of life is affected to varying degrees[5].

*Self-management is a multidimensional concept that encompasses the behaviors, cognitive processes, and emotional strategies individuals use to manage chronic health conditions and their consequences[6]. In the present study, self-management among elderly patients with a permanent enterostomy refers to the ongoing efforts to manage stoma care, prevent complications, seek information, make health-related decisions, adjust daily activities, and cope with the psychological and social changes of living with a stoma. Compared with self-care, which primarily emphasizes the performance of specific health-related tasks, self-management represents a broader construct integrating behavioral actions, cognitive regulation, and coping processes[7]. Other related concepts, such as psychological adaptation, independence, and self-efficacy, may influence or facilitate self-management but are not equivalent to it[6,8].*

*After ostomy surgery, the demands of self-management extend to every dimension of daily life, including dietary adjustments, stoma care, and social participation, and rely not only on relevant knowledge and skills but, more importantly, on an individual’s ongoing behavioral adaptation and decision-making across varying contexts[9,10].* However, with the widespread adoption of Enhanced Recovery After Surgery (ERAS) protocols, hospital stays have become progressively shorter; consequently, some patients have not yet fully developed self-management capabilities by the time of discharge. Furthermore, during the early postoperative period, *most patients struggle to adapt quickly to the altered body image associated with the stoma, and their awareness of the need to assume self-management responsibilities proactively remains relatively limited[11]. Age-related changes such as slower cognitive processing, reduced reaction time, and altered learning capacity can increase the difficulty* of mastering the knowledge and skills required for stoma care[12].Relevant studies indicate that *the overall level of self-management among elderly stoma patients remains suboptimal, with only approximately 50.4% possessing adequate disease-related knowledge[13]. Furthermore, stoma care procedures are inherently complex and technically demanding; consequently, elderly patients may find it challenging to transition from dependent care to independent self-management within a short period[14].*

*Although previous studies have explored the self-management experiences and support needs of patients with stomas, existing research has focused primarily on challenges, adaptation difficulties, and supportive needs[15]. Less attention has been paid to how elderly patients with permanent enterostomies experience the development of self-management behaviors through continuous adjustment and problem-solving during adaptation. Therefore, this study adopted a qualitative phenomenological approach to explore these experiences, aiming to inform individualized supportive interventions and improve long-term care strategies for this population*

**4 Discussion**

Our study employed in-depth interviews to explore the experiences of elderly patients living with a permanent stoma during long-term adaptation, with a particular focus on the development of self-management behaviors. The findings suggest that self-management in this population is not a static skill but a dynamic process shaped by ongoing challenges and adjustments[8]. *Three overarching themes were identified: “decision-making dilemmas and uncertainty,” “reconstructing daily life and identity after stoma formation,” and “developing resilience through accumulated experience and self-management practice.” These findings indicate that elderly patients’ self-management experiences are shaped by continuous interactions among stoma-related challenges, personal adaptation, and available support. Reflecting on their experiences after stoma formation, participants described a transition from initial uncertainty and disruption toward greater confidence in managing daily life and reconstructing personal meaning. Our findings provide a process-oriented understanding of how elderly patients gradually transform uncertainty and disruption into adaptive self-management through continuous learning, adjustment, and the use of available resources.*

*Because self-management of a permanent stoma permeates daily life* and entails continuous behavioral adjustments, elderly patients are frequently required to make decisions across *multiple* domains, including dietary choices, stoma care, and physical activity[18].Previous *research has* indicated that[19]， Chinese patients with colorectal cancer commonly experience significant decision-making dilemmas when facing colostomy surgery; the findings of the present study align with these observations. This study revealed that *when seeking health information online, patients often encounter cognitive conflicts arising from diverse and inconsistent information sources.* This *challenge* is exacerbated when online *content* contradicts *professional medical advice, thereby intensifying patients’ confusion and uncertainty in decision-making. Importantly, these decision-making difficulties were not merely emotional experiences; they directly affected patients’ capacity to initiate and maintain self-management behaviors[20], such as selecting appropriate foods, managing stoma-related complications, and deciding when to seek professional support.*However, existing decision-aid tools for colorectal cancer tend to focus primarily on disease screening and the selection of treatment regimens, *with insufficient attention to supporting long-term self-management decisions[21,22].* In addition to the complexities of the external information environment, *difficulties in processing and applying health information may further complicate decision-making among some elderly patients, particularly when information is complex or inconsistent[23]. Constrained by their health literacy, some elderly patients may struggle to understand and retain care-related information, which can undermine their capacity for independent decision-making and their confidence in doing so[24].* Given these multifaceted factors—and against the backdrop of rapidly advancing digital health technologies—it is imperative to develop scientifically sound and standardized digital decision-aid tools[22]. By providing structured, simplified, and personalized information, such tools can effectively *enhance patients’ decision-making capabilities in complex situations. Furthermore, this study observed that some patients exhibited a degree of reliance on family members during the decision-making process, highlighting the supportive role of family in self-management and healthcare decisions. This finding should be interpreted within the participants’ family context. In the present study, most participants were married and lived with family members, which may partly explain the prominent family involvement in daily stoma management and decision-making. In the context of Chinese family-oriented culture, family members often provide practical assistance and emotional support to elderly adults living with chronic conditions[25]. Therefore, with patients’ informed consent, appropriately involving family members in shared decision-making and health education processes may help improve decision quality and support long-term self-management[26]. In addition, healthcare professionals should provide individualized decision support by assessing patients’ information needs and health literacy levels, delivering structured and accessible education, and offering continuous guidance to facilitate daily self-management decisions[27].*

Previous studies have confirmed that patients with stomas frequently face alterations in body image and *psychological responses akin to a grief reaction[28]. Building on this, our findings further reveal that psychological adaptation is characterized by significant long-term, non-linear features[4,29]; even long after surgery, patients may continue to experience recurrent emotional fluctuations and identity conflicts.* From a theoretical perspective, this process resonates with frameworks such as "meaning reconstruction," *suggesting that patients do not simply return to a pre-illness state but, through continuous adjustment and adaptation, gradually construct a “new normal”* that incorporates the presence of the stoma[30]. *This psychological reconstruction may represent an important prerequisite for sustained self-management[31]. As patients gradually accepted the stoma and reconstructed their identity, they became more willing to engage in daily stoma care, seek solutions to complications, and integrate stoma management into their routines.* Based on these findings, clinical practice should prioritize both the continuity of psychological support and the recognition of *phase-specific patient needs[32]. Routine assessment of body image concerns, emotional distress, and adaptation difficulties during follow-up may help identify patients requiring additional psychological support[33]. During the early postoperative period, the focus should be on addressing patients’ body image distress and sense of loss of control, and on providing targeted psychological counseling.* In the long-term follow-up phase, it is essential to identify patients who remain in a state of psychological conflict and, through interventions such as narrative therapy and peer support groups, *facilitate meaning-making to enhance overall adaptation[34,35].*

*Our findings indicate that elderly patients living with a permanent stoma gradually developed psychological resilience through continuous self-adjustment and lived experience. The prominence of resilience in this study may be related to the cumulative life experiences of elderly adults[36]. Throughout their lives, elderly individuals may have encountered previous transitions, losses, and health-related challenges, which can serve as psychological resources for coping with the irreversible changes associated with permanent enterostomy[37]. Previous studies have often conceptualized resilience as an individual trait or a relatively stable personal resource that enables one to withstand adversity and maintain functioning[38]. However, our findings extend this perspective by suggesting that resilience among elderly patients with permanent stomas is not merely a pre-existing attribute but is gradually reconstructed through lived experience, continuous self-adjustment, and engagement in self-management practices. This interpretation aligns with contemporary resilience theories that emphasize resilience as a dynamic, developmental process shaped by person–environment interactions[39,40]. In this study, patients did not achieve effective self-management immediately after stoma formation. Instead, they gradually accumulated practical skills and confidence through repeated attempts, problem-solving, and adjustment in daily care routines. As patients became more familiar with stoma management, they shifted their attention from physical limitations toward maintaining daily activities, family relationships, and emotional well-being[41]. This process suggests that resilience functioned not only as a psychological coping resource but also as a facilitating factor supporting sustained self-management behaviors[42].While previous studies have largely focused on the psychological burden and negative experiences of stoma care[43], our findings highlight the adaptive capabilities of elderly patients and suggest that their accumulated life experiences may serve as valuable resources for reconstructing meaning, accepting bodily changes, and developing long-term self-management strategies[44]. These findings imply that interventions for elderly patients with permanent enterostomy should not focus solely on correcting deficits but should also incorporate a strength-based approach that recognizes patients’ existing abilities and life experiences. In clinical practice, healthcare professionals may encourage patients to reflect on successful self-management experiences, identify personal coping strategies, and participate in peer-support activities to enhance self-efficacy and promote sustained self-management behaviors[45,46].*

**★ 10. Removing Repetition in the Introduction**

**Reviewer’s Comment:**

**Remove unnecessary repetition in the Introduction.**

**➦Response**

Thank you for this suggestion. We have carefully reviewed the Introduction section and removed redundant descriptions. The revised Introduction has been streamlined to improve logical flow and focus more clearly on the research gap, rationale, and objectives of this study.

**★ 11. Reference Formatting According to PLOS ONE Requirements**

**Reviewer’s Comment:**

**Verify that all references follow PLOS ONE formatting.**

**➦Response**

Thank you for this reminder. We have carefully checked all references and revised their formatting according to PLOS ONE requirements. The reference list has been reviewed for consistency, completeness, and accuracy. In addition, all in-text citations were cross-checked to ensure full correspondence with the reference list. Where available, DOIs and publication details were also verified to improve citation reliability. Any discrepancies identified during this process were corrected accordingly.

**★ 12. Definition of Self-management**

**Reviewer’s Comment:**

**The paper lacks a clear, operationalized definition of "self-management" despite this being the central concept.**

**➦Response**

Thank you for highlighting the importance of conceptual clarity. We agree that a clear definition of self-management is essential for interpreting our findings. In the revised manuscript, we have added an operational definition of self-management based on chronic illness management literature and stoma care research.

In this study, self-management refers to the dynamic process through which elderly patients with permanent enterostomy acquire knowledge, develop skills, make decisions, regulate emotions, adapt daily activities, and manage stoma-related challenges to maintain health and quality of life.
 In the manuscript, this revised content is presented as follows:

*Self-management is a multidimensional concept that encompasses the behaviors, cognitive processes, and emotional strategies individuals use to manage chronic health conditions and their consequences[6]. In the present study, self-management among elderly patients with a permanent enterostomy refers to the ongoing efforts to manage stoma care, prevent complications, seek information, make health-related decisions, adjust daily activities, and cope with the psychological and social changes of living with a stoma. Compared with self-care, which primarily emphasizes the performance of specific health-related tasks, self-management represents a broader construct integrating behavioral actions, cognitive regulation, and coping processes[7]. Other related concepts, such as psychological adaptation, independence, and self-efficacy, may influence or facilitate self-management but are not equivalent to it[6,8].*

**★ 13. Conceptual Overlap Between Themes**

**Reviewer’s Comment:**

**There is conceptual overlap between themes.**

**➦Response**

Thank you for this important observation. We carefully reviewed the thematic structure and refined the descriptions and boundaries of each theme to reduce conceptual overlap. The revised themes now more clearly represent distinct aspects of participants’ experiences, including decision-making challenges, adaptation-related reconstruction, and resilience development.

**Before modification:3 Results**

Ultimately, 13 patients were selected for interviews and were labeled sequentially from "N1" to "N13." Among them, 8 were male and 5 were female, ranging in age from 60 to 77 years. The general characteristics of the interviewees are presented in Table 1. Three major themes and eight sub-themes emerged from the interviews: **(1) Decision-making dilemmas and uncertainty (insufficient and poorly tailored decision-support resources; decision-making pressure arising from the cognitive load experienced by older stoma patients); (2) The process of psychological reconstruction (changes in body image and continuity of life following stoma surgery; ambivalent attitudes—oscillating between rejection and acceptance—during the long-term adaptation process; and the gradual adaptation to life with a stoma); and (3) Resilience narratives and meaning-making in living with a permanent stoma (resilience developed through repeated self-adjustment and lived experience; acceptance and "letting go" shaped by life experiences in older age; and the reconstruction of life priorities and meaning following illness).**

**Changed:3 Results**

Ultimately, 13 patients were selected for interviews and were labeled sequentially from "N1" to "N13." Among them, 8 were male and 5 were female, ranging in age from 60 to 77 years. The general characteristics of the interviewees are presented in Table 1. Three major themes and eight sub-themes emerged from the interviews: **(1) Decision-making dilemmas and uncertainty (insufficient and poorly tailored decision-support resources; decision-making pressure arising from the cognitive load experienced by elderly stoma patients);** *(2) Reconstructing daily life and identity after stoma formation (Disruption of body image and daily routines; Emotional struggle between rejection and acceptance; and Developing a new normal in daily living); and (3) Developing resilience through accumulated experience and self-management practice (Learning through trial and error and gaining confidence; Drawing on previous life experiences to cope with irreversible changes; and the Redefining life priorities after illness).*

**★ 14. Practical Implementation of Findings**

**Reviewer’s Comment:**

**The paper identifies important challenges but does not address practical implementation.**

**➦Response**

Thank you for this valuable suggestion. We agree that qualitative findings should not only describe patients’ experiences and challenges but also provide meaningful implications for clinical practice. In response to this comment, we have expanded the **Discussion section** by adding specific clinical strategies and potential solutions based on the challenges identified in our findings, with the aim of improving the practical applicability of this study for elderly patients with permanent enterostomy.

Specifically, we now emphasize that postoperative care should move beyond standardized health education and adopt a structured, individualized, and continuous support model throughout the adaptation process. Based on participants’ experiences, we highlight the importance of tailoring education according to patients’ cognitive abilities, health literacy, and self-management needs, while providing ongoing guidance to support decision-making and problem-solving in daily stoma care. In addition, we further emphasize the important role of family caregivers in supporting patients’ self-management, particularly in daily care assistance, emotional support, and adaptation to lifestyle changes.

Furthermore, we have expanded the discussion regarding psychological and emotional support. Our findings indicate that self-management development is closely intertwined with psychological adaptation and identity reconstruction. Therefore, we highlight the need for healthcare professionals to assess patients’ emotional responses, body image concerns, and adjustment difficulties at different stages of adaptation, and to provide timely interventions such as peer support, experience sharing, and supportive counseling to facilitate acceptance and resilience development.

These revisions provide a clearer pathway for translating the findings into clinical practice and highlight the need for a holistic, patient-centered care model integrating physical management, psychological support, and long-term self-management empowerment.

To address this concern, we have added further discussion regarding the practical application of our findings and potential clinical strategies in the Discussion section (highlighted in blue in the revised manuscript):

**4 Discussion**

Because self-management of a permanent stoma permeates daily life and entails continuous behavioral adjustments, elderly patients are frequently required to make decisions across multiple domains, including dietary choices, stoma care, and physical activity[18].Previous research has indicated that[19]， Chinese patients with colorectal cancer commonly experience significant decision-making dilemmas when facing colostomy surgery; the findings of the present study align with these observations. This study revealed that when seeking health information online, patients often encounter cognitive conflicts arising from diverse and inconsistent information sources. This challenge is exacerbated when online content contradicts professional medical advice, thereby intensifying patients’ confusion and uncertainty in decision-making. Importantly, these decision-making difficulties were not merely emotional experiences; they directly affected patients’ capacity to initiate and maintain self-management behaviors[20], such as selecting appropriate foods, managing stoma-related complications, and deciding when to seek professional support.However, existing decision-aid tools for colorectal cancer tend to focus primarily on disease screening and the selection of treatment regimens, with insufficient attention to supporting long-term self-management decisions[21,22]. In addition to the complexities of the external information environment, difficulties in processing and applying health information may further complicate decision-making among some elderly patients, particularly when information is complex or inconsistent[23]. Constrained by their health literacy, some elderly patients may struggle to understand and retain care-related information, which can undermine their capacity for independent decision-making and their confidence in doing so[24]. *Given these multifaceted factors—and against the backdrop of rapidly advancing digital health technologies—it is imperative to develop scientifically sound and standardized digital decision-aid tools[22]. By providing structured, simplified, and personalized information, such tools can effectively enhance patients’ decision-making capabilities in complex situations.* Furthermore, this study observed that some patients exhibited a degree of reliance on family members during the decision-making process, highlighting the supportive role of family in self-management and healthcare decisions. This finding should be interpreted within the participants’ family context. In the present study, most participants were married and lived with family members, which may partly explain the prominent family involvement in daily stoma management and decision-making. In the context of Chinese family-oriented culture, family members often provide practical assistance and emotional support to elderly adults living with chronic conditions[25]. *Therefore, with patients’ informed consent, appropriately involving family members in shared decision-making and health education processes may help improve decision quality and support long-term self-management[26]. In addition, healthcare professionals should provide individualized decision support by assessing patients’ information needs and health literacy levels, delivering structured and accessible education, and offering continuous guidance to facilitate daily self-management decisions[27].*

Previous studies have confirmed that patients with stomas frequently face alterations in body image and psychological responses akin to a grief reaction[28]. Building on this, our findings further reveal that psychological adaptation is characterized by significant long-term, non-linear features[4,29]; even long after surgery, patients may continue to experience recurrent emotional fluctuations and identity conflicts. From a theoretical perspective, this process resonates with frameworks such as "meaning reconstruction," suggesting that patients do not simply return to a pre-illness state but, through continuous adjustment and adaptation, gradually construct a “new normal” that incorporates the presence of the stoma[30]. This psychological reconstruction may represent an important prerequisite for sustained self-management[31]. As patients gradually accepted the stoma and reconstructed their identity, they became more willing to engage in daily stoma care, seek solutions to complications, and integrate stoma management into their routines. Based on these findings, clinical practice should prioritize both the continuity of psychological support and the recognition of phase-specific patient needs[32]. Routine assessment of body image concerns, emotional distress, and adaptation difficulties during follow-up may help identify patients requiring additional psychological support[33]. *During the early postoperative period, the focus should be on addressing patients’ body image distress and sense of loss of control, and on providing targeted psychological counseling. In the long-term follow-up phase, it is essential to identify patients who remain in a state of psychological conflict and, through interventions such as narrative therapy and peer support groups, facilitate meaning-making to enhance overall adaptation[34,35].*

Our findings indicate that elderly patients living with a permanent stoma gradually developed psychological resilience through continuous self-adjustment and lived experience. The prominence of resilience in this study may be related to the cumulative life experiences of elderly adults[36]. Throughout their lives, elderly individuals may have encountered previous transitions, losses, and health-related challenges, which can serve as psychological resources for coping with the irreversible changes associated with permanent enterostomy[37]. Previous studies have often conceptualized resilience as an individual trait or a relatively stable personal resource that enables one to withstand adversity and maintain functioning[38]. However, our findings extend this perspective by suggesting that resilience among elderly patients with permanent stomas is not merely a pre-existing attribute but is gradually reconstructed through lived experience, continuous self-adjustment, and engagement in self-management practices. This interpretation aligns with contemporary resilience theories that emphasize resilience as a dynamic, developmental process shaped by person–environment interactions[39,40]. In this study, patients did not achieve effective self-management immediately after stoma formation. Instead, they gradually accumulated practical skills and confidence through repeated attempts, problem-solving, and adjustment in daily care routines. As patients became more familiar with stoma management, they shifted their attention from physical limitations toward maintaining daily activities, family relationships, and emotional well-being[41]. This process suggests that resilience functioned not only as a psychological coping resource but also as a facilitating factor supporting sustained self-management behaviors[42].While previous studies have largely focused on the psychological burden and negative experiences of stoma care[43], our findings highlight the adaptive capabilities of elderly patients and suggest that their accumulated life experiences may serve as valuable resources for reconstructing meaning, accepting bodily changes, and developing long-term self-management strategies[44]. These findings imply that interventions for elderly patients with permanent enterostomy should not focus solely on correcting deficits but should also incorporate a strength-based approach that recognizes patients’ existing abilities and life experiences. *In clinical practice, healthcare professionals may encourage patients to reflect on successful self-management experiences, identify personal coping strategies, and participate in peer-support activities to enhance self-efficacy and promote sustained self-management behaviors[45,46].*

**★ 15. Interview Duration and Single Interview Design**

**Reviewer’s Comment:**

**The wide range of 20–40 minutes with single interviews may not be sufficient for in-depth exploration. Single interviews may not capture the evolution of experiences.**

**➦Response**

Thank you for raising this important methodological consideration. We acknowledge that longitudinal interviews could provide further insight into changes in adaptation experiences over time. However, the aim of this study was to explore participants’ retrospective accounts of their adaptation experiences and self-management behaviors rather than directly observe longitudinal changes.

To ensure sufficient depth of data within a single interview, each interview lasted approximately 20–40 minutes, depending on participants’ willingness and the richness of their experiences. Although some interviews were relatively short (around 20 minutes), the semi-structured interview format with targeted follow-up probing questions enabled participants to provide detailed retrospective accounts covering the period from stoma formation to the present, including changes in self-management behaviors, challenges encountered, and adaptation processes. This approach ensured that even shorter interviews yielded meaningful and information-rich data.

We have also revised the Limitations section in the manuscript to acknowledge the single-interview design and its potential constraints regarding capturing dynamic changes over time.

**Before modification：2.4 Data collection**

This study recruited participants by posting recruitment posters at an ostomy outpatient clinic and employed a purposive sampling method to select individuals who met the inclusion criteria. Prior to the interview, the researcher established a rapport with the interviewee, provided a detailed explanation of the study's objectives, interview content, and procedures, and informed the interviewee that the entire session would be audio-recorded. Furthermore, the researcher gave assurances that all data would be used exclusively for this study, treated with strict confidentiality, and would not involve any personally identifiable information. The formal interview commenced only after the interviewee had signed an informed consent form. The interviews were conducted in a quiet, comfortable setting with adequate privacy (specifically, a demonstration room), for which the researcher had prepared an interview guide, recording equipment, and other relevant materials in advance. During the interview, the same researcher conducted face-to-face, semi-structured interviews; the session began with light conversation to ease into the discussion, and the researcher flexibly adjusted the sequence of questions based on the interview guide, utilizing probing questions to encourage the interviewee to elaborate deeply on their relevant experiences. Concurrently, the researcher strove to use plain, accessible language—avoiding technical jargon that might hinder communication—and, without interrupting the interviewee, gently guided the conversation back on topic whenever it began to drift. Throughout the interview, the researcher simultaneously documented the interviewee's non-verbal behaviors (such as facial expressions and gestures) while maintaining a consistently neutral and non-judgmental attitude. **Each interview lasted approximately 20 to 40 minutes, and each participant was interviewed once.** Upon the conclusion of each interview, the researcher promptly organized and transcribed the interview data. Data collection was terminated once informational saturation was reached—that is, when consecutive interviews yielded no new information and the interviewees had no further details to add—at which point the researcher expressed their gratitude to the participants.

**6 Limitations**

This study has several limitations. First, the relatively small sample size and recruitment from a single tertiary hospital may limit the transferability of the findings to other settings with different cultural and healthcare contexts. Second, variations in participant characteristics, such as gender distribution and postoperative duration, may have influenced the results, and some experiences may be underrepresented. Third, as interviews were conducted in Chinese and later translated into English, subtle nuances in meaning may have been affected despite careful translation procedures; additionally, recall bias may be present as participants reflected on past experiences. Finally, due to the qualitative design, this study was unable to capture the longitudinal evolution of self-management behaviors and patient needs, particularly in relation to the development of resilience and meaning-making. Future research using longitudinal designs and more diverse samples is warranted to further validate and extend these findings.

**Changed:2.4 Data collection**

*Participants were recruited through recruitment posters posted at an ostomy outpatient clinic using purposive sampling. This approach was chosen to obtain rich and diverse accounts of self-management experiences among elderly patients living with permanent enterostomy, with consideration of variations in stoma duration and individual adaptation experiences. All interviews were conducted by the first author, a male nursing graduate student with clinical experience in gastrointestinal surgery and training in qualitative research methods, including qualitative interviewing and data analysis. The interviewer had no prior therapeutic relationship with the participants, and participation or refusal did not affect their medical care. Before each interview, the researcher explained the study purpose, procedures, voluntary participation, and confidentiality principles, and written informed consent was obtained before audio recording. Face-to-face semi-structured interviews were conducted in a private demonstration room at the ostomy outpatient clinic using an interview guide. Open-ended questions and probing techniques were used to encourage participants to describe their experiences in depth, and field notes were recorded to capture contextual information and non-verbal expressions.* ***Each interview lasted approximately 20–40 minutes, depending on participants’ willingness and the richness of their experiences. Although each participant was interviewed once, the semi-structured interview approach allowed participants to retrospectively describe their experiences from stoma formation to the present, including changes in self-management behaviors, challenges encountered, and adaptation processes.and all participants were interviewed once.*** *Audio recordings were transcribed verbatim and checked for accuracy after each interview. Data collection continued until saturation was achieved; after the 10th interview, no substantially new codes emerged, and three additional interviews were conducted for confirmation. After the 13th interview, no new codes or themes were identified, and data collection was terminated.*

**6 Limitations**

This study has several limitations. First, the relatively small sample size and recruitment from a single tertiary hospital may limit the transferability of the findings to other settings with different cultural and healthcare contexts. Second, variations in participant characteristics, such as gender distribution and postoperative duration, may have influenced the results, and some experiences may be underrepresented. Third, as interviews were conducted in Chinese and later translated into English, subtle nuances in meaning may have been affected despite careful translation procedures; additionally, recall bias may be present as participants reflected on past experiences. *Finally, although participants were encouraged to retrospectively describe changes in their self-management behaviors from stoma formation to the present, data were collected through a single interview at one time point. Therefore, the findings represent participants’ retrospective accounts of their adaptation experiences rather than direct observation of changes across different stages of adaptation. Future longitudinal qualitative studies with repeated interviews at multiple time points are needed to further explore the dynamic changes in self-management behaviors and adaptation experiences*

*among elderly patients with permanent enterostomy.*

- Section 3. Response to Reviewer #2:

We sincerely appreciate your careful and insightful review of our manuscript. Your thoughtful comments have provided us with valuable guidance in improving the conceptual clarity, methodological transparency, and overall coherence of our study. We greatly appreciate your attention to the alignment between the research aim, study design, interview process, findings, and interpretation, as well as your suggestions regarding the definition of self-management, qualitative rigor, contextual description, and the cautious interpretation of age-related experiences.

In response to your valuable suggestions, we have carefully revised the manuscript to further clarify the conceptualization of self-management, strengthen the description of the research methods, improve the transparency of the qualitative procedures, and refine the interpretation and discussion of our findings. We have also revised relevant statements to avoid overgeneralization and ensure that our conclusions remain appropriately supported by the data and existing literature.

Below, we provide detailed, point-by-point responses to each of your comments. All revisions have been highlighted in blue in the revised manuscript for your convenience.

Once again, we sincerely thank you for your valuable time, constructive feedback, and professional insights. Your comments have substantially contributed to strengthening the rigor, clarity, and clinical relevance of this manuscript.

**★ 1. Clarification and Conceptualization of Self-management**

**Reviewer’s Comment:
The manuscript should clearly define self-management and distinguish it from related concepts such as self-care, psychological adaptation, independence, and self-efficacy.**

**➦Response**

Thank you for highlighting the importance of conceptual clarity. We agree that a clear definition of self-management is essential for interpreting the findings of this study. In the revised manuscript, we have added an operational definition of self-management based on chronic illness management literature and stoma care research.

We further clarified the distinction between self-management and related concepts. Specifically, self-management was conceptualized as a dynamic process involving knowledge acquisition, skill development, decision-making, symptom and complication management, daily adaptation, and resource utilization. Psychological adaptation, independence, and self-efficacy were clarified as related but distinct constructs that may influence or interact with self-management development rather than being equivalent to self-management itself.

Revised content in the manuscript:

*Self-management is a multidimensional concept that encompasses the behaviors, cognitive processes, and emotional strategies individuals use to manage chronic health conditions and their consequences[6]. In the present study, self-management among elderly patients with a permanent enterostomy refers to the ongoing efforts to manage stoma care, prevent complications, seek information, make health-related decisions, adjust daily activities, and cope with the psychological and social changes of living with a stoma. Compared with self-care, which primarily emphasizes the performance of specific health-related tasks, self-management represents a broader construct integrating behavioral actions, cognitive regulation, and coping processes[7]. Other related concepts, such as psychological adaptation, independence, and self-efficacy, may influence or facilitate self-management but are not equivalent to it[6,8].*

[6]	Miller WR, Lasiter S, Bartlett Ellis R, Buelow JM. Chronic disease self-management: a hybrid concept analysis. Nurs Outlook. 2015 Mar-Apr;63(2):154-61. doi: 10.1016/j.outlook.2014.07.005. Epub 2014 Aug 12. PMID: 25241136; PMCID: PMC4326608.

[7]	Martínez, N.; Connelly, C. D.; Pérez, A.; Calero, P. Self-Care: A Concept Analysis. Int. J. Nurs. Sci. 2021, 8 (4), 418–425.

[8]	Schulman-Green D, Jaser S, Martin F, Alonzo A, Grey M, McCorkle R, Redeker NS, Reynolds N, Whittemore R. Processes of self-management in chronic illness. J Nurs Scholarsh. 2012 Jun;44(2):136-44. doi: 10.1111/j.1547-5069.2012.01444.x. Epub 2012 May 2. PMID: 22551013; PMCID: PMC3366425.

**★ 2. Alignment Between Study Aim, Design, Findings, and Discussion**

**Reviewer’s Comment:
The manuscript would benefit from improved alignment between the stated aim, study design, findings, and discussion. The findings currently appear to emphasize psychological adaptation more than the formation and evolution of practical self-management behaviours.**

**➦Response**

Thank you for this insightful comment. We acknowledge that the original manuscript did not sufficiently clarify the relationship between self-management behaviors and psychological adaptation. In the revised manuscript, we have strengthened the alignment between the study aim, interview focus, findings, and discussion.

We have further emphasized that psychological adaptation represents an important contextual process that influences the development of self-management behaviors, rather than being the primary outcome itself. The Results section has been refined to highlight participants’ learning, adjustment, decision-making, problem-solving, and gradual development of self-management strategies. The Discussion and Conclusion sections have also been moderated to avoid overstating psychological adaptation and to better reflect the process of self-management development.

➦**Comparison before and after modification**

**Before modification：Abstract**

**Object**
This study aimed to explore the development of self-management behaviors and experiences among elderly patients with a permanent stoma during long-term adaptation, providing a theoretical basis for developing targeted intervention strategies.

**1 Introduction**

Although existing studies have explored the self-care experiences and needs of patients with stomas, most have focused primarily on describing the associated problems, lacking an in-depth analysis of how self-management behaviors are formed and evolve during the long-term adaptation process—particularly within the demographic of elderly patients with permanent intestinal stomas. Consequently, this study employs a qualitative research approach, utilizing in-depth interviews with elderly patients with permanent intestinal stomas to investigate the formation and evolution of their self-management behaviors during the long-term adaptation process. The aim is to provide a theoretical foundation and practical reference for the development of future intervention strategies.

**3 Results**

Ultimately, 13 patients were selected for interviews and were labeled sequentially from "N1" to "N13." Among them, 8 were male and 5 were female, ranging in age from 60 to 77 years. The general characteristics of the interviewees are presented in Table 1. Three major themes and eight sub-themes emerged from the interviews: **(1) Decision-making dilemmas and uncertainty (insufficient and poorly tailored decision-support resources; decision-making pressure arising from the cognitive load experienced by older stoma patients); (2) The process of psychological reconstruction (changes in body image and continuity of life following stoma surgery; ambivalent attitudes—oscillating between rejection and acceptance—during the long-term adaptation process; and the gradual adaptation to life with a stoma); and (3) Resilience narratives and meaning-making in living with a permanent stoma (resilience developed through repeated self-adjustment and lived experience; acceptance and "letting go" shaped by life experiences in older age; and the reconstruction of life priorities and meaning following illness).**

**4 Discussion**

Our study employed in-depth interviews to explore the experiences of elderly patients living with a permanent stoma during long-term adaptation, with a particular focus on the development of self-management behaviors. The findings suggest that self-management in this population is not a static skill but a dynamic process shaped by ongoing challenges and adjustments14. Three overarching themes emerged: “decision-making dilemmas and uncertainty,” “psychological reconstruction during adaptation,” and “resilience narratives and meaning-making.” Together, these findings highlight that elderly patients gradually move from uncertainty and disrupted life experiences toward more stable self-management and redefined life meaning.

**Changed：Abstract**

**Object**
*This study aimed to explore the experiences and perceived changes in self-management behaviors among elderly patients with a permanent stoma during adaptation, providing insights for the development of targeted supportive interventions.*

**1 Introduction**

*Although previous studies have explored the self-management experiences and support needs of patients with stomas, existing research has focused primarily on challenges, adaptation difficulties, and supportive needs[15]. Less attention has been paid to how elderly patients with permanent enterostomies experience the development of self-management behaviors through continuous adjustment and problem-solving during adaptation. Therefore, this study adopted a qualitative phenomenological approach to explore these experiences, aiming to inform individualized supportive interventions and improve long-term care strategies for this population.*

**3 Results**

Ultimately, 13 patients were selected for interviews and were labeled sequentially from "N1" to "N13." Among them, 8 were male and 5 were female, ranging in age from 60 to 77 years. The general characteristics of the interviewees are presented in Table 1. Three major themes and eight sub-themes emerged from the interviews: **(1) Decision-making dilemmas and uncertainty (insufficient and poorly tailored decision-support resources; decision-making pressure arising from the cognitive load experienced by elderly stoma patients);** *(2) Reconstructing daily life and identity after stoma formation (Disruption of body image and daily routines; Emotional struggle between rejection and acceptance; and Developing a new normal in daily living); and (3) Developing resilience through accumulated experience and self-management practice (Learning through trial and error and gaining confidence; Drawing on previous life experiences to cope with irreversible changes; and the Redefining life priorities after illness).*

*Three themes reflecting different aspects of the long-term adaptation process were identified: (1) decision-making dilemmas and uncertainty, reflecting the difficulties encountered by patients after stoma formation; (2) reconstructing daily life and identity, describing how patients adjusted to bodily and lifestyle changes; and (3) developing resilience through accumulated experience and self-management practice, illustrating how patients gradually transformed challenges into personal resources.*

**4 Discussion**

Our study employed in-depth interviews to explore the experiences of elderly patients living with a permanent stoma during long-term adaptation, with a particular focus on the development of self-management behaviors. The findings suggest that self-management in this population is not a static skill but a dynamic process shaped by ongoing challenges and adjustments[8]. *Three overarching themes were identified: “decision-making dilemmas and uncertainty,” “reconstructing daily life and identity after stoma formation,” and “developing resilience through accumulated experience and self-management practice.” These findings indicate that elderly patients’ self-management experiences are shaped by continuous interactions among stoma-related challenges, personal adaptation, and available support. Reflecting on their experiences after stoma formation, participants described a transition from initial uncertainty and disruption toward greater confidence in managing daily life and reconstructing personal meaning. Our findings provide a process-oriented understanding of how elderly patients gradually transform uncertainty and disruption into adaptive self-management through continuous learning, adjustment, and the use of available resources.*

**★ 3. Study Setting and Participant Characteristics**

**Reviewer’s Comment:
Greater detail is needed regarding the study setting and participant characteristics.**

**➦Response**

Thank you for this helpful suggestion. We have expanded the description of the study setting and participant characteristics to provide greater contextual information and improve the transferability of the findings.

Additional details regarding the clinical setting, recruitment source, and participant characteristics have been added to allow readers to better understand the context in which participants’ experiences were explored.

In the manuscript, this revised section now appears as follows:

**2 Methods**

**2.1 Study design**

This study employs a phenomenological research approach to explore the experiences and needs associated with the self-management process from the patient's perspective. This approach was selected because it focuses on understanding individuals’ subjective experiences and the meanings they attribute to a particular phenomenon, which was consistent with the aim of this study to explore patients’ experiences of self-management during long-term adaptation after enterostomy. Through semi-structured in-depth interviews, the study investigates the self-management behaviors of elderly patients with permanent intestinal stomas during their long-term adaptation process. *The research was conducted from March 25 to May 5, 2026, within the Department of Gastrointestinal Surgery and the Stoma Clinic of a tertiary hospital in the Inner Mongolia Autonomous Region, China. The hospital provides specialized care for patients with colorectal diseases, and the Stoma Clinic offers postoperative follow-up, stoma care education, and complication management services for patients with enterostomies.* The sample size was determined based on the principle of data saturation; specifically, when interviews ceased to yield new themes or information, a small number of additional participants were recruited to verify data saturation, and data collection was terminated once the sufficiency of information was confirmed[16].

**2.4 Data collection**

*Participants were recruited through recruitment posters posted at an ostomy outpatient clinic using purposive sampling. This approach was chosen to obtain rich and diverse accounts of self-management experiences among elderly patients living with permanent enterostomy, with consideration of variations in stoma duration and individual adaptation experiences. All interviews were conducted by the first author, a male nursing graduate student with clinical experience in gastrointestinal surgery and training in qualitative research methods, including qualitative interviewing and data analysis. The interviewer had no prior therapeutic relationship with the participants, and participation or refusal did not affect their medical care. Before each interview, the researcher explained the study purpose, procedures, voluntary participation, and confidentiality principles, and written informed consent was obtained before audio recording. Face-to-face semi-structured interviews were conducted in a private demonstration room at the ostomy outpatient clinic using an interview guide. Open-ended questions and probing techniques were used to encourage participants to describe their experiences in depth, and field notes were recorded to capture contextual information and non-verbal expressions. Each interview lasted approximately 20–40 minutes, depending on participants’ willingness and the richness of their experiences. Although each participant was interviewed once, the semi-structured interview approach allowed participants to retrospectively describe their experiences from stoma formation to the present, including changes in self-management behaviors, challenges encountered, and adaptation processes.and all participants were interviewed once. Audio recordings were transcribed verbatim and checked for accuracy after each interview. Data collection continued until saturation was achieved; after the 10th interview, no substantially new codes emerged, and three additional interviews were conducted for confirmation. After the 13th interview, no new codes or themes were identified, and data collection was terminated.*

➦**Comparison before and after modification**

**Before modification:**

**S1 Table 1. Characteristics of participants (n = 13)**

| Participant ID | Gender | Age | Educational Background | Marital Status | Duration of Ostomy | Ostomy Type |
| --- | --- | --- | --- | --- | --- | --- |
| N1 | Female | 62 | Junior high | Married | 4 years | Colostomy |
| N2 | Male | 60 | University | Married | 2 years | Colostomy |
| N3 | Female | 70 | Junior high | Married | 2 years | Colostomy |
| N4 | Male | 74 | High school | Married | 3 years | Colostomy |
| N5 | Male | 75 | Junior high | Married | 1 year | Ileostomy |
| N6 | Female | 69 | High school | Married | 2 years | Colostomy |
| N7 | Male | 66 | High school | divorced | 5 years | Ileostomy |
| N8 | Male | 68 | Junior high | Married | 3 years | Colostomy |
| N9 | Male | 72 | High school | Married | 7 years | Ileostomy |
| N10 | Female | 63 | Junior high | Married | 2 years | Colostomy |
| N11 | Male | 77 | High school | widowers | 4 years | Ileostomy |
| N12 | Female | 63 | Junior high | Married | 6 years | Colostomy |
| N13 | Male | 65 | High school | divorced | 3 years | Ileostomy |

**Changed:**

***S1 Table 1****. Characteristics of participants (n = 13)*

| *variable* | *Category* | *N（%）* |
| --- | --- | --- |
| *Gender* | *Female* | *5（38.5）* |
|  | *Male* | *8（61.5）* |
| *Age（years）* | *60-69* | *8(61.5)* |
|  | *70-79* | *5(38.5)* |
| *Educational Background* | *University* | *1（7.7）* |
|  | *High school* | *6（46.15）* |
|  | *Junior high* | *6（46.15）* |
| *Marital Status* | *Married* | *10（76.9）* |
|  | *single* | *0（0）* |
|  | *widowed* | *1（7.7）* |
|  | *divorced* | *2（15.4）* |
| *Duration of Ostomy* | *1-5years* | *11（84.6）* |
|  | *≥6years* | *2（15.4）* |
| *Type of surgery* | *Colostomy* | *8（61.5）* |
|  | *Ileostomy* | *5（38.5）* |
| *Primary diagnosis* | *Rectal cancer* | *4（30.8）* |
|  | *Colon cancer* | *9（69.2）* |
| *Stoma-related complications* | *Yes* | *7（53.8）* |
|  | *No* | *6（46.2）* |
| *Living arrangement* | *Living alone* | *3（23.1）* |
|  | *Living with family* | *10（76.9）* |

**★ 4. Duration Since Stoma Formation and Sampling Rationale**

**Reviewer’s Comment:
More information is needed regarding time since stoma formation and sampling rationale.**

**➦Response**

Thank you for this important suggestion. We have added more detailed information regarding participant characteristics to improve the transparency and transferability of our qualitative findings. Specifically, the duration since stoma formation has already been presented in ***S1 Table: Characteristics of Participants (See above)***, and this information has been highlighted in blue in the revised manuscript.

Participants were recruited based on predefined inclusion criteria to ensure that they had sufficient experience living with a permanent enterostomy and could provide rich descriptions of their long-term adaptation and self-management experiences. The sampling strategy was further clarified as purposive sampling aimed at obtaining information-rich cases relevant to the research question.

The revised description of the sampling strategy in the Methods section is as follows (highlighted in blue in the revised manuscript):

**2.4 Data collection**

*Participants were recruited through recruitment posters posted at an ostomy outpatient clinic using purposive sampling. This approach was chosen to obtain rich and diverse accounts of self-management experiences among elderly patients living with permanent enterostomy, with consideration of variations in stoma duration and individual adaptation experiences. All interviews were conducted by the first author, a male nursing graduate student with clinical experience in gastrointestinal surgery and training in qualitative research methods, including qualitative interviewing and data analysis. The interviewer had no prior therapeutic relationship with the participants, and participation or refusal did not affect their medical care. Before each interview, the researcher explained the study purpose, procedures, voluntary participation, and confidentiality principles, and written informed consent was obtained before audio recording. Face-to-face semi-structured interviews were conducted in a private demonstration room at the ostomy outpatient clinic using an interview guide. Open-ended questions and probing techniques were used to encourage participants to describe their experiences in depth, and field notes were recorded to capture contextual information and non-verbal expressions. Each interview lasted approximately 20–40 minutes, depending on participants’ willingness and the richness of their experiences. Although each participant was interviewed once, the semi-structured interview approach allowed participants to retrospectively describe their experiences from stoma formation to the present, including changes in self-management behaviors, challenges encountered, and adaptation processes.and all participants were interviewed once. Audio recordings were transcribed verbatim and checked for accuracy after each interview. Data collection continued until saturation was achieved; after the 10th interview, no substantially new codes emerged, and three additional interviews were conducted for confirmation. After the 13th interview, no new codes or themes were identified, and data collection was terminated.*

**★ 5. Interview Guide Development and Theoretical Basis**

**Reviewer’s Comment:
Greater detail is needed regarding interview guide development.**

**➦Response**

Thank you for this valuable suggestion. We have provided additional details regarding the development of the semi-structured interview guide. The interview questions were developed based on the study aim, previous literature regarding stoma self-management and adaptation, and clinical experience related to elderly patients living with permanent enterostomy.

Furthermore, pilot interviews were conducted before formal data collection, and the interview guide was refined based on feedback from these interviews to improve clarity and facilitate deeper exploration of participants’ experiences.

Specifically, we revised Section **2.5 “Develop an Interview Outline”** as follows:

**2.5 Develop an Interview Outline**

*The interview guide was developed based on an in-depth literature review and informed by the self-management framework[8], which emphasizes the acquisition of self-management skills, problem-solving, support utilization, and adaptation among individuals living with chronic conditions. The questions were designed to explore elderly patients’ experiences, challenges, behavioral changes, and support needs related to stoma self-management during adaptation to permanent enterostomy. The preliminary interview guide was reviewed by experts in stoma care and psychology, and appropriate modifications were made based on their feedback. Two pilot interviews were then conducted with elderly patients with permanent enterostomies who met the inclusion criteria but were not included in the final sample. Based on feedback from the pilot interviews, three modifications were made: (1) temporal anchoring was added to Question 1 to facilitate participants’ recall of the learning process; (2) probing prompts (e.g., “Can you describe a specific situation?”) were incorporated to encourage detailed narratives; and (3) technical terms were replaced with plain language to improve comprehensibility among elderly participants with varying health literacy levels. Following these refinements, the final version of the interview guide was established as follows:*

**★ 6. Reflexivity and Trustworthiness Procedures**

**Reviewer’s Comment:
Greater detail is needed regarding reflexivity and trustworthiness procedures.**

**➦Response**

Thank you for raising this important issue regarding qualitative rigor. We have expanded the Methods section to provide additional information regarding reflexivity and strategies used to enhance trustworthiness.

The revised manuscript now describes the researcher’s role, reflexive practices, coding procedures, and strategies used to minimize potential bias, including repeated discussions among research team members, comparison and refinement of codes, and consensus-based development of themes.

Specifically, we revised Section **2.7 “Rigour and reflexivity”** to provide further clarification regarding the reflexive process and strategies used to ensure methodological rigor, as follows:

**2.7 Rigour and reflexivity**

*To enhance the methodological rigor of this qualitative study, the four criteria proposed by Lincoln and Guba, including credibility, dependability, confirmability, and transferability, were considered throughout the research process.* ***Credibility was enhanced through purposive sampling to recruit participants with diverse experiences of living with a permanent enterostomy. Two researchers independently coded the interview transcripts, and coding results were compared and discussed within the research team until consensus was reached. To ensure dependability, detailed records of participant recruitment, data collection, and data analysis procedures were maintained to establish an audit trail. All transcripts were repeatedly reviewed and compared with the original audio recordings to ensure accuracy. To enhance confirmability, reflexive practices were incorporated throughout data analysis. The interviewer maintained reflexive notes documenting personal assumptions, reflections, and methodological decisions during data collection and interpretation. To minimize researcher bias during coding and interpretation, two researchers independently coded the interview transcripts, and coding results were compared and discussed within the research team until consensus was reached. Regular team discussions were conducted to identify potential biases and ensure that themes were grounded in participants’ narratives rather than researchers’ preconceptions. To enhance transferability, detailed descriptions of the research setting, participant characteristics, and sampling criteria were provided to allow readers to assess the applicability of the findings to other contexts. Data collection continued until data saturation was achieved. Data saturation was assessed throughout the data collection and analysis process.*** *After the 10th interview, no substantially new codes were identified from the interview transcripts. However, to confirm the stability and comprehensiveness of the emerging findings, three additional interviews were conducted. After the 13th interview, no new codes or themes emerged, and the research team determined that sufficient information had been obtained to answer the research question. Therefore, participant recruitment and data collection were terminated.*

**★ 7. Moderating Claims Regarding Cognitive Decline and Behavioral Change**

**Reviewer’s Comment:
Claims about cognitive decline and behavioural change over time should be presented more cautiously and supported by stronger evidence.**

**➦Response**

Thank you for this important observation. We have carefully reviewed statements related to cognitive decline and behavioral changes over time and revised them to avoid overgeneralization or age-related assumptions.

In response, we have carefully revised the relevant sections throughout the manuscript. Specifically, statements that previously presented age-related cognitive decline as a general characteristic of elderly patients have been rephrased to reflect participants’ self-reported difficulties in remembering or processing information, rather than implying objectively measured cognitive impairment. In addition, descriptions of behavioural change over time have been moderated to clarify that these accounts are based on participants’ retrospective narratives rather than longitudinal observation. Corresponding adjustments have also been made in the Discussion and Limitations sections to ensure consistency and appropriate caution. All revisions have been clearly highlighted in the revised manuscript with track changes.

The corresponding revised text is highlighted in blue in the revised manuscript and presented as follows:

➦**Comparison before and after modification**

**Before modification：1 Introduction**

Following ostomy surgery, self-management permeates every aspect of a patient's daily life, encompassing dietary adjustments, stoma care, and participation in social activities. This process relies not only on relevant knowledge and skills but also, more importantly, involves the individual's continuous behavioral adjustments and decision-making across various contexts6,7.However, with the widespread adoption of Enhanced Recovery After Surgery (ERAS) protocols, hospital stays have become progressively shorter; consequently, some patients have not yet fully developed self-management capabilities by the time of discharge. Furthermore, during the early postoperative period, most patients struggle to promptly adapt to the changes in body image resulting from the presence of a stoma, and their awareness regarding the proactive assumption of self-management responsibilities remains relatively insufficient8.Among elderly patients with intestinal stomas, the aforementioned issues are particularly pronounced. **As they age, these patients often experience characteristics such as cognitive decline, diminished reaction times, and reduced learning capacity, which increase the difficulty of mastering the knowledge and skills required for stoma care9.**Relevant studies indicate that the overall level of self-management among elderly stoma patients remains low, with only approximately 50.4% possessing an adequate level of relevant knowledge10.Furthermore, the procedures involved in stoma care are inherently complex and technically demanding; consequently, elderly patients often struggle to successfully navigate the transition from a role of "dependent care" to one of "independent self-care" within a short timeframe11.

**3.1.2 Decision-Making Pressure Caused by Cognitive Load in Elderly Ostomy Patients**

Elderly ostomy patients experience cognitive decline, leading to difficulties in understanding, integrating, and applying information. This makes them more prone to cognitive load when facing complex postoperative management and multiple choices, thus exacerbating decision-making pressure.

**4 Discussion**

Our study employed in-depth interviews to explore the experiences of elderly patients living with a permanent stoma during long-term adaptation, with a particular focus on the development of self-management behaviors. **The findings suggest that self-management in this population is not a static skill but a dynamic process shaped by ongoing challenges and adjustments14.** Three overarching themes emerged: “decision-making dilemmas and uncertainty,” “psychological reconstruction during adaptation,” and “resilience narratives and meaning-making.” Together, these findings highlight that elderly patients gradually move from uncertainty and disrupted life experiences toward more stable self-management and redefined life meaning.

Our findings indicate that elderly patients living with a permanent stoma gradually cultivate psychological resilience through continuous self-adjustment and lived experience. **Rather than achieving self-management instantaneously, these patients progressively accumulate skills and build confidence through a process of repeated trial and error embedded within their daily care routines.** Concurrently, the wealth of life experience accumulated during their later years appears to foster a more pragmatic and accepting attitude toward physical changes26. Patients often shift their focus away from the limitations of their physical functioning, placing greater emphasis instead on maintaining daily living activities, family relationships, and emotional well-being27. While previous studies have largely focused on the psychological burden and negative experiences associated with stoma care28, our findings specifically highlight the adaptive capabilities possessed by elderly patients. Specifically, their accumulated life experience was identified as a crucial coping resource, providing robust support for their acceptance of reality and their process of self-adjustment29. These findings suggest that interventions for this patient population should not be confined solely to addressing their problems and deficits; rather, they should also acknowledge and build upon their existing capabilities and invaluable experiences. By guiding patients to reflect on their positive experiences during the adaptation process, clinicians can help enhance their self-confidence and sense of self-efficacy, thereby fostering the continuous improvement of their long-term self-management skills30.

**Changed：1 Introduction**

*After ostomy surgery, the demands of self-management extend to every dimension of daily life, including dietary adjustments, stoma care, and social participation, and rely not only on relevant knowledge and skills but, more importantly, on an individual’s ongoing behavioral adaptation and decision-making across varying contexts[9,10].* However, with the widespread adoption of Enhanced Recovery After Surgery (ERAS) protocols, hospital stays have become progressively shorter; consequently, some patients have not yet fully developed self-management capabilities by the time of discharge. Furthermore, during the early postoperative period, *most patients struggle to adapt quickly to the altered body image associated with the stoma, and their awareness of the need to assume self-management responsibilities proactively remains relatively limited[11]. Age-related changes such as slower cognitive processing, reduced reaction time, and altered learning capacity can increase the difficulty of mastering the knowledge and skills required for stoma care[12].*Relevant studies indicate that *the overall level of self-management among elderly stoma patients remains suboptimal, with only approximately 50.4% possessing adequate disease-related knowledge[13]. Furthermore, stoma care procedures are inherently complex and technically demanding; consequently, elderly patients may find it challenging to transition from dependent care to independent self-management within a short period[14].*

[12]	Wang SM, Jiang JL, Li R, Wang JJ, Gu CH, Zeng J, Wei XH, Chen M. Qualitative exploration of home life experiences and care needs among elderly patients with temporary intestinal stomas. World J Gastroenterol. 2024 Jun 14;30(22):2893-2901. doi: 10.3748/wjg.v30.i22.2893. PMID: 38947295; PMCID: PMC11212711.

***3.1.2 Decision-making challenges when processing complex self-management information***

*Some older participants reported difficulties in understanding, retaining, and applying complex stoma care information. When faced with multiple or inconsistent sources of advice, these challenges increased their uncertainty and made decision-making more difficult.*

**4 Discussion**

Our study employed in-depth interviews to explore the experiences of elderly patients

living with a permanent stoma during long-term adaptation, with a particular focus on the development of self-management behaviors. *The findings suggest that participants perceived self-management as an evolving experience shaped by ongoing challenges, adjustments, and accumulated experiences[8]. Three overarching themes were identified: “decision-making dilemmas and uncertainty,” “reconstructing daily life and identity after stoma formation,” and “developing resilience through accumulated experience and self-management practice.” These findings indicate that elderly patients’ self-management experiences are shaped by continuous interactions among stoma-related challenges, personal adaptation, and available support. Reflecting on their experiences after stoma formation, participants described a transition from initial uncertainty and disruption toward greater confidence in managing daily life and reconstructing personal meaning. Our findings provide a process-oriented understanding of how elderly patients gradually transform uncertainty and disruption into adaptive self-management through continuous learning, adjustment, and the use of available resources.*

*Our findings indicate that elderly patients living with a permanent stoma gradually developed psychological resilience through continuous self-adjustment and lived experience. The prominence of resilience in this study may be related to the cumulative life experiences of elderly adults[36]. Throughout their lives, elderly individuals may have encountered previous transitions, losses, and health-related challenges, which can serve as psychological resources for coping with the irreversible changes associated with permanent enterostomy[37]. Previous studies have often conceptualized resilience as an individual trait or a relatively stable personal resource that enables one to withstand adversity and maintain functioning[38]. However, our findings extend this perspective by suggesting that resilience among elderly patients with permanent stomas is not merely a pre-existing attribute but is gradually reconstructed through lived experience, continuous self-adjustment, and engagement in self-management practices. This interpretation aligns with contemporary resilience theories that emphasize resilience as a dynamic, developmental process shaped by person–environment interactions[39,40]. In this study, patients did not achieve effective self-management immediately after stoma formation. Instead, they gradually accumulated practical skills and confidence through repeated attempts, problem-solving, and adjustment in daily care routines. As patients became more familiar with stoma management, they shifted their attention from physical limitations toward maintaining daily activities, family relationships, and emotional well-being[41]. This process suggests that resilience functioned not only as a psychological coping resource but also as a facilitating factor supporting sustained self-management behaviors[42].While previous studies have largely focused on the psychological burden and negative experiences of stoma care[43], our findings highlight the adaptive capabilities of elderly patients and suggest that their accumulated life experiences may serve as valuable resources for reconstructing meaning, accepting bodily changes, and developing long-term self-management strategies[44]. These findings imply that interventions for elderly patients with permanent enterostomy should not focus solely on correcting deficits but should also incorporate a strength-based approach that recognizes patients’ existing abilities and life experiences. In clinical practice, healthcare professionals may encourage patients to reflect on successful self-management experiences, identify personal coping strategies, and participate in peer-support activities to enhance self-efficacy and promote sustained self-management behaviors[45,46].*

**★ 8. Avoiding Potentially Ageist or Overgeneralized Statements**

**Reviewer’s Comment:
The manuscript should avoid potentially ageist or overgeneralised statements.**

**➦Response**

Thank you for highlighting this important issue. We have carefully reviewed the manuscript and revised expressions that may unintentionally imply generalized assumptions about older adults.

The revised manuscript now emphasizes individual variability among elderly patients and avoids attributing experiences solely to chronological age. Instead, we consider the combined influence of health status, personal history, social support, and adaptation experiences.

➦**Comparison before and after modification**

**Before modification：Abstract**

**Results**
Three overarching themes and several sub-themes were identified. Theme one, decision-making dilemmas and uncertainty, included insufficient and poorly tailored decision support resources and **increased cognitive burden in older age.** Theme two, psychological reconstruction during adaptation, encompassed disruption of body image and daily life, ambivalence between rejection and acceptance, and gradual adaptation to living with a stoma. Theme three, resilience narratives and meaning-making, involved resilience developed through repeated self-adjustment, acceptance shaped by life experience, and reconstruction of life priorities following illness.

**4 Discussion**

Our findings indicate that elderly patients living with a permanent stoma gradually cultivate psychological resilience through continuous self-adjustment and lived experience. Rather than achieving self-management instantaneously, these patients progressively accumulate skills and build confidence through a process of repeated trial and error embedded within their daily care routines. Concurrently, the wealth of life experience accumulated during their later years appears to foster a more pragmatic and accepting attitude toward physical changes26. Patients often shift their focus away from the limitations of their physical functioning, placing greater emphasis instead on maintaining daily living activities, family relationships, and emotional well-being27. While previous studies have largely focused on the psychological burden and negative experiences associated with stoma care28, our findings specifically highlight the adaptive capabilities possessed by elderly patients. Specifically, their accumulated life experience was identified as a crucial coping resource, providing robust support for their acceptance of reality and their process of self-adjustment29. These findings suggest that interventions for this patient population should not be confined solely to addressing their problems and deficits; rather, they should also acknowledge and build upon their existing capabilities and invaluable experiences. By guiding patients to reflect on their positive experiences during the adaptation process, clinicians can help enhance their self-confidence and sense of self-efficacy, thereby fostering the continuous improvement of their long-term self-management skills30.

**Changed：Abstract**

**Results**

Three overarching themes and several sub-themes were identified. Theme one, decision-making dilemmas and uncertainty, included insufficient and poorly tailored decision support resources ***and challenges in processing complex self-management information.*** *Theme two, reconstructing daily life and identity after stoma formation, encompassed disruptions in body image and daily routines, emotional struggles between rejection and acceptance, and the development of a new normal in living with a stoma. Theme three, developing resilience through accumulated experience and self-management practice, involved learning through repeated self-adjustment, drawing on previous life experiences as coping resources, and redefining life priorities after illness.*

**4 Discussion**

*Our findings indicate that elderly patients living with a permanent stoma gradually developed psychological resilience through continuous self-adjustment and lived experience. The prominence of resilience in this study may be related to the cumulative life experiences of elderly adults[36]. Throughout their lives, elderly individuals may have encountered previous transitions, losses, and health-related challenges, which can serve as psychological resources for coping with the irreversible changes associated with permanent enterostomy[37]. Previous studies have often conceptualized resilience as an individual trait or a relatively stable personal resource that enables one to withstand adversity and maintain functioning[38]. However, our findings extend this perspective by suggesting that resilience among elderly patients with permanent stomas is not merely a pre-existing attribute but is gradually reconstructed through lived experience, continuous self-adjustment, and engagement in self-management practices. This interpretation aligns with contemporary resilience theories that emphasize resilience as a dynamic, developmental process shaped by person–environment interactions[39,40]. In this study, patients did not achieve effective self-management immediately after stoma formation. Instead, they gradually accumulated practical skills and confidence through repeated attempts, problem-solving, and adjustment in daily care routines. As patients became more familiar with stoma management, they shifted their attention from physical limitations toward maintaining daily activities, family relationships, and emotional well-being[41]. This process suggests that resilience functioned not only as a psychological coping resource but also as a facilitating factor supporting sustained self-management behaviors[42].While previous studies have largely focused on the psychological burden and negative experiences of stoma care[43], our findings highlight the adaptive capabilities of elderly patients and suggest that their accumulated life experiences may serve as valuable resources for reconstructing meaning, accepting bodily changes, and developing long-term self-management strategies[44]. These findings imply that interventions for elderly patients with permanent enterostomy should not focus solely on correcting deficits but should also incorporate a strength-based approach that recognizes patients’ existing abilities and life experiences. In clinical practice, healthcare professionals may encourage patients to reflect on successful self-management experiences, identify personal coping strategies, and participate in peer-support activities to enhance self-efficacy and promote sustained self-management behaviors[45,46].*

**★ 9. Terminology Standardization**

**Reviewer’s Comment:
The manuscript should standardize terminology.**

**➦ Response:**
Thank you for this suggestion. We have carefully reviewed the manuscript and standardized terminology related to self-management, adaptation, psychological adjustment, and stoma-related experiences to ensure conceptual consistency throughout the text.

**★ 10. Proposed Interventions and Evidence Support**

**Reviewer’s Comment:
Proposed interventions should be presented more cautiously and with stronger support from the data or literature.**

**➦ Response:**

Thank you for this valuable comment. We have revised the Discussion section to ensure that proposed clinical implications are appropriately grounded in our findings and existing literature.

Rather than presenting interventions as definitive solutions, we now describe them as potential strategies that may support elderly patients’ adaptation and self-management development. These suggestions are discussed in relation to the challenges identified by participants and are supported by relevant literature where appropriate.

The specific revisions are as follows:

**4 Discussion**

Our study employed in-depth interviews to explore the experiences of elderly patients living with a permanent stoma during long-term adaptation, with a particular focus on the development of self-management behaviors. *The findings suggest that participants perceived self-management as an evolving experience shaped by ongoing challenges, adjustments, and accumulated experiences[8]. Three overarching themes were identified: “decision-making dilemmas and uncertainty,” “reconstructing daily life and identity after stoma formation,” and “developing resilience through accumulated experience and self-management practice.” These findings indicate that elderly patients’ self-management experiences are shaped by continuous interactions among stoma-related challenges, personal adaptation, and available support. Reflecting on their experiences after stoma formation, participants described a transition from initial uncertainty and disruption toward greater confidence in managing daily life and reconstructing personal meaning. Our findings provide a process-oriented understanding of how elderly patients gradually transform uncertainty and disruption into adaptive self-management through continuous learning, adjustment, and the use of available resources.*

*Because self-management of a permanent stoma permeates daily life* and entails continuous behavioral adjustments, elderly patients are frequently required to make decisions across *multiple* domains, including dietary choices, stoma care, and physical activity[18].Previous *research has* indicated that[19]， Chinese patients with colorectal cancer commonly experience significant decision-making dilemmas when facing colostomy surgery; the findings of the present study align with these observations. This study revealed that *when seeking health information online, patients often encounter cognitive conflicts arising from diverse and inconsistent information sources.* This *challenge* is exacerbated when online *content* contradicts *professional medical advice, thereby intensifying patients’ confusion and uncertainty in decision-making. Importantly, these decision-making difficulties were not merely emotional experiences; they directly affected patients’ capacity to initiate and maintain self-management behaviors[20], such as selecting appropriate foods, managing stoma-related complications, and deciding when to seek professional support.*However, existing decision-aid tools for colorectal cancer tend to focus primarily on disease screening and the selection of treatment regimens, *with insufficient attention to supporting long-term self-management decisions[21,22].* In addition to the complexities of the external information environment, *difficulties in processing and applying health information may further complicate decision-making among some elderly patients, particularly when information is complex or inconsistent[23]. Constrained by their health literacy, some elderly patients may struggle to understand and retain care-related information, which can undermine their capacity for independent decision-making and their confidence in doing so[24].* **Given these multifaceted factors—and against the backdrop of rapidly advancing digital health technologies—it is imperative to develop scientifically sound and standardized digital decision-aid tools[22].** By providing structured, simplified, and personalized information, such tools can effectively *enhance patients’ decision-making capabilities in complex situations. Furthermore, this study observed that some patients exhibited a degree of reliance on family members during the decision-making process, highlighting the supportive role of family in self-management and healthcare decisions. This finding should be interpreted within the participants’ family context. In the present study, most participants were married and lived with family members, which may partly explain the prominent family involvement in daily stoma management and decision-making. In the context of Chinese family-oriented culture, family members often provide practical assistance and emotional support to elderly adults living with chronic conditions[25].* ***Therefore, with patients’ informed consent, appropriately involving family members in shared decision-making and health education processes may help improve decision quality and support long-term self-management[26]. In addition, healthcare professionals should provide individualized decision support by assessing patients’ information needs and health literacy levels, delivering structured and accessible education, and offering continuous guidance to facilitate daily self-management decisions[27].***

Previous studies have confirmed that patients with stomas frequently face alterations in body image and *psychological responses akin to a grief reaction[28]. Building on this, our findings further reveal that psychological adaptation is characterized by significant long-term, non-linear features[4,29]; even long after surgery, patients may continue to experience recurrent emotional fluctuations and identity conflicts.* From a theoretical perspective, this process resonates with frameworks such as "meaning reconstruction," *suggesting that patients do not simply return to a pre-illness state but, through continuous adjustment and adaptation, gradually construct a “new normal”* that incorporates the presence of the stoma[30]. *This psychological reconstruction may represent an important prerequisite for sustained self-management[31]. As patients gradually accepted the stoma and reconstructed their identity, they became more willing to engage in daily stoma care, seek solutions to complications, and integrate stoma management into their routines.* Based on these findings, clinical practice should prioritize both the continuity of psychological support and the recognition of *phase-specific patient needs[32].* ***Routine assessment of body image concerns, emotional distress, and adaptation difficulties during follow-up may help identify patients requiring additional psychological support[33]. During the early postoperative period, the focus should be on addressing patients’ body image distress and sense of loss of control, and on providing targeted psychological counseling.* In the long-term follow-up phase, it is essential to identify patients who remain in a state of psychological conflict and, through interventions such as narrative therapy and peer support groups, *facilitate meaning-making to enhance overall adaptation[34,35].***

*Our findings indicate that elderly patients living with a permanent stoma gradually developed psychological resilience through continuous self-adjustment and lived experience. The prominence of resilience in this study may be related to the cumulative life experiences of elderly adults[36]. Throughout their lives, elderly individuals may have encountered previous transitions, losses, and health-related challenges, which can serve as psychological resources for coping with the irreversible changes associated with permanent enterostomy[37]. Previous studies have often conceptualized resilience as an individual trait or a relatively stable personal resource that enables one to withstand adversity and maintain functioning[38]. However, our findings extend this perspective by suggesting that resilience among elderly patients with permanent stomas is not merely a pre-existing attribute but is gradually reconstructed through lived experience, continuous self-adjustment, and engagement in self-management practices. This interpretation aligns with contemporary resilience theories that emphasize resilience as a dynamic, developmental process shaped by person–environment interactions[39,40]. In this study, patients did not achieve effective self-management immediately after stoma formation. Instead, they gradually accumulated practical skills and confidence through repeated attempts, problem-solving, and adjustment in daily care routines. As patients became more familiar with stoma management, they shifted their attention from physical limitations toward maintaining daily activities, family relationships, and emotional well-being[41]. This process suggests that resilience functioned not only as a psychological coping resource but also as a facilitating factor supporting sustained self-management behaviors[42].While previous studies have largely focused on the psychological burden and negative experiences of stoma care[43], our findings highlight the adaptive capabilities of elderly patients and suggest that their accumulated life experiences may serve as valuable resources for reconstructing meaning, accepting bodily changes, and developing long-term self-management strategies[44].* ***These findings imply that interventions for elderly patients with permanent enterostomy should not focus solely on correcting deficits but should also incorporate a strength-based approach that recognizes patients’ existing abilities and life experiences. In clinical practice, healthcare professionals may encourage patients to reflect on successful self-management experiences, identify personal coping strategies, and participate in peer-support activities to enhance self-efficacy and promote sustained self-management behaviors[45,46].***

**★ 11. Refinement of Discussion and Conclusion**

**Reviewer’s Comment:
The discussion and conclusion should be moderated accordingly.**

**➦ Response:**

**Thank you for this constructive suggestion. We have carefully revised the Discussion and Conclusion sections to ensure that interpretations remain consistent with the qualitative findings.The revised manuscript places greater emphasis on the dynamic process of developing self-management behaviors and avoids making claims beyond the scope of the data. We have also clarified that psychological adaptation, resilience, and self-management are interconnected but conceptually distinct aspects of the adaptation experience.**

**Revised content in the manuscript:**

****5 Conclusion****

*This qualitative study explored elderly patients’ experiences of self-management behaviors during adaptation to permanent enterostomy. The findings suggest that patients experienced self-management as a dynamic process shaped by decisional uncertainty, non-linear psychological adjustment, and the development of resilience. Accordingly, healthcare professionals should pay greater attention to long-term self-management support in this population by strengthening individualized decision support, providing continuous psychological care, appropriately involving family members according to patients’ preferences, and adopting a strength-based care approach.*

- Section 4. Response to Reviewer #3:

**Thank you for your time and for reviewing our manuscript. We have carefully considered all comments and suggestions provided during the review process and revised the manuscript accordingly. All revisions have been highlighted in blue in the revised manuscript for ease of identification.**

- Section 5. Response to Reviewer #4:

We sincerely appreciate your thorough and thoughtful review of our manuscript. We are grateful for your recognition of the significance of our study and the value of exploring the developmental process of self-management among elderly patients living with permanent enterostomy. Your insightful comments have provided us with important guidance for improving the alignment between the research aim, interview design, methodological transparency, and interpretation of findings.

We have carefully considered each of your comments and revised the manuscript accordingly. Specifically, we have further clarified the development of the interview guide, sampling rationale, data saturation process, contextual background of participants, interpretation of study contributions, and researcher positionality. These revisions have strengthened the methodological rigor, transparency, and clinical relevance of our qualitative study.

Below, we provide detailed, point-by-point responses to each of your comments. All revisions have been highlighted in blue in the revised manuscript for your convenience.

Once again, we sincerely thank you for your valuable time, constructive feedback, and professional insights, which have substantially contributed to improving the quality and clarity of our manuscript.

**★ 1. Alignment Between Interview Questions and Study Aim**

**Reviewer’s Comment:
There is a noticeable disconnect between the interview questions and the study’s stated aim. While the research seeks to explore the developmental process of self-management, the questions do not sufficiently probe the evolution of behaviors across different phases of adaptation. Because the interviews were conducted as a single, one-time event, they captured a relatively fluid narrative rather than distinct phases. A longitudinal design with multiple time-point interviews would have better addressed this aim.**

**➦ Response:**

Thank you very much for this insightful and constructive comment. We agree that the original wording of our study aim and title may have implied a stronger focus on the longitudinal evolution of self-management behaviors than could be fully captured through a single interview design. We appreciate your careful observation regarding the alignment between the study aim, interview guide, and research design.

In response to this comment, we have revised the title and study aim to more accurately reflect the scope of this qualitative inquiry. The revised version focuses on exploring elderly patients’ experiences of self-management behaviors during adaptation to permanent enterostomy rather than implying a direct observation of behavioral changes across different time points.

Furthermore, we have revised the interview guide to include more process-oriented questions that encouraged participants to retrospectively describe how they learned stoma-related management skills, encountered challenges, adjusted their behaviors, and developed strategies during their adaptation journey. These modifications have strengthened the alignment between the interview questions and the revised study aim.

We also acknowledge that a longitudinal qualitative design with repeated interviews at multiple time points would provide deeper insights into the dynamic evolution of self-management behaviors. Therefore, we have added this limitation to the revised manuscript and highlighted that future longitudinal studies are needed to further explore changes in self-management development across different adaptation stages.

Revised content in the manuscript:

**Abstract**

**Object**

*This study aimed to explore the experiences and perceived changes in self-management behaviors among elderly patients with a permanent stoma during adaptation, providing insights for the development of targeted supportive interventions.*

**2.5 Develop an Interview Outline**

*The interview guide was developed based on an in-depth literature review and informed by the self-management framework[8], which emphasizes the acquisition of self-management skills, problem-solving, support utilization, and adaptation among individuals living with chronic conditions. The questions were designed to explore elderly patients’ experiences, challenges, behavioral changes, and support needs related to stoma self-management during adaptation to permanent enterostomy. The preliminary interview guide was reviewed by experts in stoma care and psychology, and appropriate modifications were made based on their feedback. Two pilot interviews were then conducted with elderly patients with permanent enterostomies who met the inclusion criteria but were not included in the final sample. Based on feedback from the pilot interviews, three modifications were made: (1) temporal anchoring was added to Question 1 to facilitate participants’ recall of the learning process; (2) probing prompts (e.g., “Can you describe a specific situation?”) were incorporated to encourage detailed narratives; and (3) technical terms were replaced with plain language to improve comprehensibility among elderly participants with varying health literacy levels. Following these refinements, the final version of the interview guide was established as follows:*

*(1) From discharge to the present, could you describe how you learned to manage your stoma and how your self-management behaviors changed over time?*

*(2) What difficulties or challenges did you encounter while learning and managing your stoma, and how did you cope with them?*

(3) How do you typically perform your stoma care? Do you usually complete it yourself, or do you require assistance from others?

(4) How has undergoing stoma surgery impacted your daily life?

(5) Reflecting on the entire adaptation process, what forms of support do you consider to have been most important?

(6) What expectations or concerns do you have regarding your future life?

**6 Limitations**

This study has several limitations. First, the relatively small sample size and recruitment from a single tertiary hospital may limit the transferability of the findings to other settings with different cultural and healthcare contexts. Second, variations in participant characteristics, such as gender distribution and postoperative duration, may have influenced the results, and some experiences may be underrepresented. Third, as interviews were conducted in Chinese and later translated into English, subtle nuances in meaning may have been affected despite careful translation procedures; additionally, recall bias may be present as participants reflected on past experiences. *Finally, although participants were encouraged to retrospectively describe changes in their self-management behaviors from stoma formation to the present, data were collected through a single interview at one time point. Therefore, the findings represent participants’ retrospective accounts of their adaptation experiences rather than direct observation of changes across different stages of adaptation. Future longitudinal qualitative studies with repeated interviews at multiple time points are needed to further explore the dynamic changes in self-management behaviors and adaptation experiences among elderly patients with permanent enterostomy.*

**2.2 Inclusion and Exclusion Criteria**

Inclusion Criteria: ①Age≥60 years;②Willingness to reflect deeply on interview questions and engage in self-disclosure;③Clinically diagnosed and having undergone permanent intestinal stoma surgery;④Postoperative period≥3 months, and currently in the post-discharge home adaptation phase. Exclusion Criteria:①Subjects experiencing severe emotional fluctuations or withdrawing from the study midway;②Acute deterioration of the medical condition during the course of the study.

*Although participants differed in time since stoma formation, this variation was considered valuable because the study aimed to explore diverse adaptation experiences rather than compare specific postoperative stages.*

# **★ 2. Comparability of Participants at Different Adaptation Stages**

**Reviewer’s Comment:
The study includes patients at markedly different stages of long-term adaptation (e.g., 4 months versus several years post-surgery), yet it does not adequately justify the comparability of these experiences.**

**➦ Response:**

Thank you for this important comment. We agree that providing sufficient information regarding participants’ duration of living with a stoma is essential for understanding the context and transferability of qualitative findings.

In response to this suggestion, we have added the variable time since stoma formation to the participant characteristics table (S1 Table: Characteristics of Participants, n = 13) to provide a more comprehensive description of the sample. In addition, we have further clarified in the Methods section that although participants differed in their duration of living with a stoma, this variation was considered valuable because the aim of this study was to explore diverse adaptation experiences and the development of self-management behaviors among elderly patients with permanent enterostomy, rather than to compare experiences across specific postoperative stages.

These revisions provide greater transparency regarding participant characteristics and clarify the rationale for including individuals with different durations of stoma experience.

The specific revisions are as follows:

**2.2 Inclusion and Exclusion Criteria**

Inclusion Criteria: ①Age≥60 years;②Willingness to reflect deeply on interview questions and engage in self-disclosure;③Clinically diagnosed and having undergone permanent intestinal stoma surgery;④Postoperative period≥3 months, and currently in the post-discharge home adaptation phase. Exclusion Criteria:①Subjects experiencing severe emotional fluctuations or withdrawing from the study midway;②Acute deterioration of the medical condition during the course of the study.

*Although participants differed in time since stoma formation, this variation was considered valuable because the study aimed to explore diverse adaptation experiences rather than compare specific postoperative stages.*

***S1 Table 1****. Characteristics of participants (n = 13)*

| *variable* | *Category* | *N（%）* |
| --- | --- | --- |
| *Gender* | *Female* | *5（38.5）* |
|  | *Male* | *8（61.5）* |
| *Age（years）* | *60-69* | *8(61.5)* |
|  | *70-79* | *5(38.5)* |
| *Educational Background* | *University* | *1（7.7）* |
|  | *High school* | *6（46.15）* |
|  | *Junior high* | *6（46.15）* |
| *Marital Status* | *Married* | *10（76.9）* |
|  | *single* | *0（0）* |
|  | *widowed* | *1（7.7）* |
|  | *divorced* | *2（15.4）* |
| ***Duration of Ostomy*** | *1-5years* | *11（84.6）* |
|  | *≥6years* | *2（15.4）* |
| *Type of surgery* | *Colostomy* | *8（61.5）* |
|  | *Ileostomy* | *5（38.5）* |
| *Primary diagnosis* | *Rectal cancer* | *4（30.8）* |
|  | *Colon cancer* | *9（69.2）* |
| *Stoma-related complications* | *Yes* | *7（53.8）* |
|  | *No* | *6（46.2）* |
| *Living arrangement* | *Living alone* | *3（23.1）* |
|  | *Living with family* | *10（76.9）* |

# **★ 3. Justification of Data Saturation With 13 Participants**

**Reviewer’s Comment:
With only 13 participants, the claim of data saturation is difficult to justify. The researchers should provide a more detailed account of how saturation was determined.**

**➦ Response:**

Thank you for this valuable comment. We agree that a clear description of the saturation process is essential for qualitative rigor.

In the revised manuscript, we have provided additional details regarding how data saturation was assessed. During the interview and analysis process, the research team continuously compared newly emerging data with existing codes and themes. After the 10th interview, no substantially new codes or concepts emerged, and the final three interviews were conducted to confirm the stability and completeness of the identified themes. Therefore, data collection was concluded when thematic sufficiency was achieved.

➦**Comparison before and after modification**

**Before modification：2.4 Data collection**

This study recruited participants by posting recruitment posters at an ostomy outpatient clinic and employed a purposive sampling method to select individuals who met the inclusion criteria. Prior to the interview, the researcher established a rapport with the interviewee, provided a detailed explanation of the study's objectives, interview content, and procedures, and informed the interviewee that the entire session would be audio-recorded. Furthermore, the researcher gave assurances that all data would be used exclusively for this study, treated with strict confidentiality, and would not involve any personally identifiable information. The formal interview commenced only after the interviewee had signed an informed consent form. The interviews were conducted in a quiet, comfortable setting with adequate privacy (specifically, a demonstration room), for which the researcher had prepared an interview guide, recording equipment, and other relevant materials in advance. During the interview, the same researcher conducted face-to-face, semi-structured interviews; the session began with light conversation to ease into the discussion, and the researcher flexibly adjusted the sequence of questions based on the interview guide, utilizing probing questions to encourage the interviewee to elaborate deeply on their relevant experiences. Concurrently, the researcher strove to use plain, accessible language—avoiding technical jargon that might hinder communication—and, without interrupting the interviewee, gently guided the conversation back on topic whenever it began to drift. Throughout the interview, the researcher simultaneously documented the interviewee's non-verbal behaviors (such as facial expressions and gestures) while maintaining a consistently neutral and non-judgmental attitude. Each interview lasted approximately 20 to 40 minutes, and each participant was interviewed once. Upon the conclusion of each interview, the researcher promptly organized and transcribed the interview data. Data collection was terminated once informational saturation was reached—that is, when consecutive interviews yielded no new information and the interviewees had no further details to add—at which point the researcher expressed their gratitude to the participants.

**Changed:2.4 Data collection**

Participants were recruited through recruitment posters posted at an ostomy outpatient clinic using purposive sampling. This approach was chosen to obtain rich and diverse accounts of self-management experiences among elderly patients living with permanent enterostomy, with consideration of variations in stoma duration and individual adaptation experiences. All interviews were conducted by the first author, a male nursing graduate student with clinical experience in gastrointestinal surgery and training in qualitative research methods, including qualitative interviewing and data analysis. The interviewer had no prior therapeutic relationship with the participants, and participation or refusal did not affect their medical care. Before each interview, the researcher explained the study purpose, procedures, voluntary participation, and confidentiality principles, and written informed consent was obtained before audio recording. Face-to-face semi-structured interviews were conducted in a private demonstration room at the ostomy outpatient clinic using an interview guide. Open-ended questions and probing techniques were used to encourage participants to describe their experiences in depth, and field notes were recorded to capture contextual information and non-verbal expressions. Each interview lasted approximately 20–40 minutes, depending on participants’ willingness and the richness of their experiences. Although each participant was interviewed once, the semi-structured interview approach allowed participants to retrospectively describe their experiences from stoma formation to the present, including changes in self-management behaviors, challenges encountered, and adaptation processes.and all participants were interviewed once. Audio recordings were transcribed verbatim and checked for accuracy after each interview. Data collection continued until saturation was achieved; *after the 10th interview, no substantially new codes emerged, and three additional interviews were conducted for confirmation. After the 13th interview, no new codes or themes were identified, and data collection was terminated.*

**★ 4. Socioeconomic and Cultural Context of Participants**

**Reviewer’s Comment:
The socioeconomic and cultural backgrounds of participants are not described. Given the variability in family structures and their influence on attitudes toward illness and aging, particularly within the Inner Mongolian healthcare and cultural context, this information is important.**

**➦ Response:**

Thank you for highlighting the importance of contextual information in qualitative research. We agree that understanding the social and cultural background of participants can facilitate interpretation and transferability of findings.

In the revised manuscript, we have added additional contextual information regarding participants’ educational background, marital status, living arrangements, and family support situations. We have also expanded the Discussion section to acknowledge the potential influence of regional cultural characteristics and family-centered support patterns within the Inner Mongolian context on patients’ adaptation experiences.

**Revised content in the manuscript:**
Additional participant characteristics have been added to Supporting Information (S1 Table), and contextual interpretation has been strengthened in the Discussion section.

**➦Comparison before and after modification**

**Before modification：4 Discussion**

Since the self-management of a permanent stoma permeates a patient's daily life and entails continuous behavioral adjustments, elderly patients are frequently required to make decisions across various domains, including dietary choices, stoma care, and participation in physical activities15.Previous studies have indicated that16，Chinese patients with colorectal cancer commonly experience significant decision-making dilemmas when facing colostomy surgery; the findings of the present study align with these observations. This study revealed that during the process of seeking information online, patients often encounter cognitive conflicts stemming from the diversity of information sources and the inconsistency of their content. This issue is particularly exacerbated when online information contradicts the advice provided by healthcare professionals, thereby intensifying elderly patients' confusion and sense of uncertainty regarding decision-making.However, existing decision-aid tools for colorectal cancer tend to focus primarily on disease screening and the selection of treatment regimens, while paying insufficient attention to providing decision support within the context of long-term self-management following stoma surgery17,18.In addition to the complexities of the external information environment, age-related cognitive decline constitutes another significant factor that compounds decision-making difficulties for elderly patients. Constrained by their levels of health literacy, elderly patients often struggle to comprehend and retain care-related information, which, to a certain extent, undermines their capacity for independent decision-making as well as their confidence in doing so19.Given these multifaceted factors—and against the backdrop of rapidly advancing digital health technologies—it is imperative to develop scientifically sound and standardized digital decision-aid tools. By providing structured, simplified, and personalized information, such tools can effectively enhance patients' decision-making capabilities within complex circumstances. Furthermore, this study observed that some patients exhibit a degree of reliance on family members during the decision-making process, underscoring the critical supportive role that the family plays in patients' decisions. Consequently—and contingent upon the patient's informed consent—integrating family members into the shared decision-making and health education processes can contribute to improved decision quality and enhanced health outcomes for patients20.

**Changed：4 Discussion**

*Because self-management of a permanent stoma permeates daily life* and entails continuous behavioral adjustments, elderly patients are frequently required to make decisions across *multiple* domains, including dietary choices, stoma care, and physical activity[18].Previous *research has* indicated that[19]， Chinese patients with colorectal cancer commonly experience significant decision-making dilemmas when facing colostomy surgery; the findings of the present study align with these observations. This study revealed that *when seeking health information online, patients often encounter cognitive conflicts arising from diverse and inconsistent information sources.* This *challenge* is exacerbated when online *content* contradicts *professional medical advice, thereby intensifying patients’ confusion and uncertainty in decision-making. Importantly, these decision-making difficulties were not merely emotional experiences; they directly affected patients’ capacity to initiate and maintain self-management behaviors[20], such as selecting appropriate foods, managing stoma-related complications, and deciding when to seek professional support.* However, existing decision-aid tools for colorectal cancer tend to focus primarily on disease screening and the selection of treatment regimens, *with insufficient attention to supporting long-term self-management decisions[21,22].* In addition to the complexities of the external information environment, *difficulties in processing and applying health information may further complicate decision-making among some elderly patients, particularly when information is complex or inconsistent[23]. Constrained by their health literacy, some elderly patients may struggle to understand and retain care-related information, which can undermine their capacity for independent decision-making and their confidence in doing so[24].* Given these multifaceted factors—and against the backdrop of rapidly advancing digital health technologies—it is imperative to develop scientifically sound and standardized digital decision-aid tools[22]. By providing structured, simplified, and personalized information, such tools can effectively *enhance patients’ decision-making capabilities in complex situations.* ***Furthermore, this study observed that some patients exhibited a degree of reliance on family members during the decision-making process, highlighting the supportive role of family in self-management and healthcare decisions. This finding should be interpreted within the participants’ family context. In the present study, most participants were married and lived with family members, which may partly explain the prominent family involvement in daily stoma management and decision-making. In the context of Chinese family-oriented culture, family members often provide practical assistance and emotional support to elderly adults living with chronic conditions[25]. Therefore, with patients’ informed consent, appropriately involving family members in shared decision-making and health education processes may help improve decision quality and support long-term self-management[26].*** *In addition, healthcare professionals should provide individualized decision support by assessing patients’ information needs and health literacy levels, delivering structured and accessible education, and offering continuous guidance to facilitate daily self-management decisions[27].*

# **★ 5. Overstatement of Novelty**

**Reviewer’s Comment:
Labeling the identified themes as “novel” appears overstated, as similar themes are extensively discussed in existing literature.**

**➦ Response:**

Thank you for this thoughtful observation. We agree that describing the themes as completely “novel” may overstate the contribution of our findings. Therefore, we have revised the wording throughout the manuscript to avoid implying that these experiences have not been previously reported.

Instead, we now emphasize that the contribution of this study lies in providing a deeper understanding of how elderly patients with permanent enterostomy experience the development of self-management behaviors over time, particularly the interaction between practical management, psychological adjustment, and accumulated life experiences.

**Revised content in the manuscript:**
Statements regarding the novelty and contribution of the study have been revised throughout the Introduction and Discussion sections.

➦**Comparison before and after modification**

**Before modification**:1 Introduction

As one of the most common malignancies, colorectal cancer (CRC) poses a severe threat to human health due to its high incidence and mortality rates.According to the Global Cancer Statistics released by the International Agency for Research on Cancer (IARC) in 20221, newly diagnosed cases of CRC accounted for 9.6% of all cancer cases, while its mortality rate constituted 9.3% of all cancer-related deaths. Ostomy surgery is a common surgical intervention for CRC; it involves surgically creating an opening in the abdominal wall through which a segment of the intestine is brought out to serve as a substitute for the anus2.This procedure effectively alleviates patient symptoms, prevents disease progression, and extends survival3.However, due to factors such as disease progression or the specific surgical approach employed, some patients require a stoma for an extended period—or even for the remainder of their lives—resulting in a permanent intestinal ostomy.

Unlike temporary stomas, a permanent intestinal stoma implies that patients must contend—over an extended period—with issues such as altered physiological functions and the restructuring of their lifestyle4.Postoperatively, patients are required not only to cope with stoma-related complications and symptoms of discomfort but also to constantly adapt their behavioral patterns in daily life; consequently, their quality of life is generally affected to varying degrees5.Therefore, enhancing the self-management capabilities of patients with permanent intestinal stomas is of significant importance.

Following ostomy surgery, self-management permeates every aspect of a patient's daily life, encompassing dietary adjustments, stoma care, and participation in social activities. This process relies not only on relevant knowledge and skills but also, more importantly, involves the individual's continuous behavioral adjustments and decision-making across various contexts6,7.However, with the widespread adoption of Enhanced Recovery After Surgery (ERAS) protocols, hospital stays have become progressively shorter; consequently, some patients have not yet fully developed self-management capabilities by the time of discharge. Furthermore, during the early postoperative period, most patients struggle to promptly adapt to the changes in body image resulting from the presence of a stoma, and their awareness regarding the proactive assumption of self-management responsibilities remains relatively insufficient8.Among elderly patients with intestinal stomas, the aforementioned issues are particularly pronounced. As they age, these patients often experience characteristics such as cognitive decline, diminished reaction times, and reduced learning capacity, which increase the difficulty of mastering the knowledge and skills required for stoma care9.Relevant studies indicate that the overall level of self-management among elderly stoma patients remains low, with only approximately 50.4% possessing an adequate level of relevant knowledge10.Furthermore, the procedures involved in stoma care are inherently complex and technically demanding; consequently, elderly patients often struggle to successfully navigate the transition from a role of "dependent care" to one of "independent self-care" within a short timeframe11.

Although existing studies have explored the self-care experiences and needs of patients with stomas, most have focused primarily on describing the associated problems, lacking an in-depth analysis of how self-management behaviors are formed and evolve during the long-term adaptation process—particularly within the demographic of elderly patients with permanent intestinal stomas. Consequently, this study employs a qualitative research approach, utilizing in-depth interviews with elderly patients with permanent intestinal stomas to investigate the formation and evolution of their self-management behaviors during the long-term adaptation process. The aim is to provide a theoretical foundation and practical reference for the development of future intervention strategies.

4 Discussion

Our study employed in-depth interviews to explore the experiences of elderly patients living with a permanent stoma during long-term adaptation, with a particular focus on the development of self-management behaviors. The findings suggest that self-management in this population is not a static skill but a dynamic process shaped by ongoing challenges and adjustments14. Three overarching themes emerged: “decision-making dilemmas and uncertainty,” “psychological reconstruction during adaptation,” and “resilience narratives and meaning-making.” Together, these findings highlight that elderly patients gradually move from uncertainty and disrupted life experiences toward more stable self-management and redefined life meaning.

Since the self-management of a permanent stoma permeates a patient's daily	life and entails continuous behavioral adjustments, elderly patients are frequently required to make	decisions across various domains, including dietary choices, stoma care, and participation in physical activities15.Previous studies have indicated that16，Chinese patients with colorectal cancer commonly experience significant decision-making dilemmas when facing colostomy surgery; the findings of the present study align with these observations. This study revealed that during the process of seeking information online, patients often encounter cognitive conflicts stemming from the diversity of information sources and the inconsistency of their content. This issue is particularly exacerbated when online information contradicts the advice provided by healthcare professionals, thereby intensifying elderly patients' confusion and sense of uncertainty regarding decision-making.However, existing decision-aid tools for colorectal cancer tend to focus primarily on disease screening and the selection of treatment regimens, while paying insufficient attention to providing decision support within the context of long-term self-management following stoma surgery17,18.In addition to the complexities of the external information environment, age-related cognitive decline constitutes another significant factor that compounds decision-making difficulties for elderly patients. Constrained by their levels of health literacy, elderly patients often struggle to comprehend and retain care-related information, which, to a certain extent, undermines their capacity for independent decision-making as well as their confidence in doing so19.Given these multifaceted factors—and against the backdrop of rapidly advancing digital health technologies—it is imperative to develop scientifically sound and standardized digital decision-aid tools. By providing structured, simplified, and personalized information, such tools can effectively enhance patients' decision-making capabilities within complex circumstances. Furthermore, this study observed that some patients exhibit a degree of reliance on family members during the decision-making process, underscoring the critical supportive role that the family plays in patients' decisions. Consequently—and contingent upon the patient's informed consent—integrating family members into the shared decision-making and health education processes can contribute to improved decision quality and enhanced health outcomes for patients20.

Previous studies have confirmed that patients with stomas frequently face alterations in body image and psychological experiences akin to a grief response21. Building upon this foundation, the present study further reveals that this process of psychological adaptation is characterized by significant long-term and non-linear features; specifically, even a considerable time after surgery, patients may continue to experience recurrent emotional fluctuations and conflicts regarding their sense of identity. From a theoretical perspective, this process resonates with theoretical frameworks such as "meaning reconstruction," suggesting that patients do not merely revert to their pre-operative state but, rather, through a continuous process of adjustment and adaptation, gradually construct a "new normal" that incorporates the presence of the stoma22. Based on these findings, clinical practice should prioritize both the continuity of psychological support and the recognition of phase-specific differences in patient needs23. During the early post-operative period, the primary focus should be on addressing patients' distress regarding body image and their sense of loss of control, providing targeted psychological counseling. In the long-term follow-up phase, it is essential to identify patients who remain in a state of psychological conflict and, through interventions such as narrative therapy and peer support groups, facilitate their integration of meaning to enhance their overall level of adaptation24,25.

Our findings indicate that elderly patients living with a permanent stoma gradually cultivate psychological resilience through continuous self-adjustment and lived experience. Rather than achieving self-management instantaneously, these patients progressively accumulate skills and build confidence through a process of repeated trial and error embedded within their daily care routines. Concurrently, the wealth of life experience accumulated during their later years appears to foster a more pragmatic and accepting attitude toward physical changes26. Patients often shift their focus away from the limitations of their physical functioning, placing greater emphasis instead on maintaining daily living activities, family relationships, and emotional well-being27. While previous studies have largely focused on the psychological burden and negative experiences associated with stoma care28, our findings specifically highlight the adaptive capabilities possessed by elderly patients. Specifically, their accumulated life experience was identified as a crucial coping resource, providing robust support for their acceptance of reality and their process of self-adjustment29. These findings suggest that interventions for this patient population should not be confined solely to addressing their problems and deficits; rather, they should also acknowledge and build upon their existing capabilities and invaluable experiences. By guiding patients to reflect on their positive experiences during the adaptation process, clinicians can help enhance their self-confidence and sense of self-efficacy, thereby fostering the continuous improvement of their long-term self-management skills30.

**Changed:**1 Introduction

*Colorectal cancer (CRC) is one of the most common malignancies and poses a s ignificant threat to human health because of its high incidence and mortality rates. According to Global Cancer Statistics 2022 released by the International Agency for Research on Cancer (IARC)[1],* newly diagnosed cases of CRC accounted for 9.6% of all cancer cases, *and CRC-related deaths* constituted 9.3% of all cancer-related deaths. *Ostomy surgery, a common intervention for CRC, involves creating an opening in the abdominal* wall through which a segment of the intestine is *exteriorized* to serve as a substitute for the anus[2]. This procedure effectively alleviates symptoms, prevents disease progression, and extends survival[3].However, *owing to* factors such as disease advancement or the specific surgical approach employed, some patients require a stoma for an extended period—or even for *life*—resulting in a permanent intestinal ostomy.

Unlike temporary stomas, a permanent intestinal stoma implies that patients must contend *over the long term* with issues such as altered physiological function and restructuring of their lifestyle[4]. Postoperatively, patients *need not only* to cope with stoma-related complications and discomfort but also to *continuously adapt their daily behaviors;* consequently, their quality of life is affected to varying degrees[5].

*Self-management is a multidimensional concept that encompasses the behaviors, cognitive processes, and emotional strategies individuals use to manage chronic health conditions and their consequences[6]. In the present study, self-management among elderly patients with a permanent enterostomy refers to the ongoing efforts to manage stoma care, prevent complications, seek information, make health-related decisions, adjust daily activities, and cope with the psychological and social changes of living with a stoma. Compared with self-care, which primarily emphasizes the performance of specific health-related tasks, self-management represents a broader construct integrating behavioral actions, cognitive regulation, and coping processes[7]. Other related concepts, such as psychological adaptation, independence, and self-efficacy, may influence or facilitate self-management but are not equivalent to it[6,8].*

*After ostomy surgery, the demands of self-management extend to every dimension of daily life, including dietary adjustments, stoma care, and social participation, and rely not only on relevant knowledge and skills but, more importantly, on an individual’s ongoing behavioral adaptation and decision-making across varying contexts[9,10].* However, with the widespread adoption of Enhanced Recovery After Surgery (ERAS) protocols, hospital stays have become progressively shorter; consequently, some patients have not yet fully developed self-management capabilities by the time of discharge. Furthermore, during the early postoperative period, *most patients struggle to adapt quickly to the altered body image associated with the stoma, and their awareness of the need to assume self-management responsibilities proactively remains relatively limited[11]. Age-related changes such as slower cognitive processing, reduced reaction time, and altered learning capacity can increase the difficulty* of mastering the knowledge and skills required for stoma care[12].Relevant studies indicate that *the overall level of self-management among elderly stoma patients remains suboptimal, with only approximately 50.4% possessing adequate disease-related knowledge[13]. Furthermore, stoma care procedures are inherently complex and technically demanding; consequently, elderly patients may find it challenging to transition from dependent care to independent self-management within a short period[14].*

*Although previous studies have explored the self-management experiences and support needs of patients with stomas, existing research has focused primarily on challenges, adaptation difficulties, and supportive needs[15]. Less attention has been paid to how elderly patients with permanent enterostomies experience the development of self-management behaviors through continuous adjustment and problem-solving during adaptation. Therefore, this study adopted a qualitative phenomenological approach to explore these experiences, aiming to inform individualized supportive interventions and improve long-term care strategies for this population*

**4 Discussion**

Our study employed in-depth interviews to explore the experiences of elderly patients living with a permanent stoma during long-term adaptation, with a particular focus on the development of self-management behaviors. The findings suggest that self-management in this population is not a static skill but a dynamic process shaped by ongoing challenges and adjustments[8]. *Three overarching themes were identified: “decision-making dilemmas and uncertainty,” “reconstructing daily life and identity after stoma formation,” and “developing resilience through accumulated experience and self-management practice.” These findings indicate that elderly patients’ self-management experiences are shaped by continuous interactions among stoma-related challenges, personal adaptation, and available support. Reflecting on their experiences after stoma formation, participants described a transition from initial uncertainty and disruption toward greater confidence in managing daily life and reconstructing personal meaning. Our findings provide a process-oriented understanding of how elderly patients gradually transform uncertainty and disruption into adaptive self-management through continuous learning, adjustment, and the use of available resources.*

*Because self-management of a permanent stoma permeates daily life* and entails continuous behavioral adjustments, elderly patients are frequently required to make decisions across *multiple* domains, including dietary choices, stoma care, and physical activity[18].Previous *research has* indicated that[19]， Chinese patients with colorectal cancer commonly experience significant decision-making dilemmas when facing colostomy surgery; the findings of the present study align with these observations. This study revealed that *when seeking health information online, patients often encounter cognitive conflicts arising from diverse and inconsistent information sources.* This *challenge* is exacerbated when online *content* contradicts *professional medical advice, thereby intensifying patients’ confusion and uncertainty in decision-making. Importantly, these decision-making difficulties were not merely emotional experiences; they directly affected patients’ capacity to initiate and maintain self-management behaviors[20], such as selecting appropriate foods, managing stoma-related complications, and deciding when to seek professional support.*However, existing decision-aid tools for colorectal cancer tend to focus primarily on disease screening and the selection of treatment regimens, *with insufficient attention to supporting long-term self-management decisions[21,22].* In addition to the complexities of the external information environment, *difficulties in processing and applying health information may further complicate decision-making among some elderly patients, particularly when information is complex or inconsistent[23]. Constrained by their health literacy, some elderly patients may struggle to understand and retain care-related information, which can undermine their capacity for independent decision-making and their confidence in doing so[24].* Given these multifaceted factors—and against the backdrop of rapidly advancing digital health technologies—it is imperative to develop scientifically sound and standardized digital decision-aid tools[22]. By providing structured, simplified, and personalized information, such tools can effectively *enhance patients’ decision-making capabilities in complex situations. Furthermore, this study observed that some patients exhibited a degree of reliance on family members during the decision-making process, highlighting the supportive role of family in self-management and healthcare decisions. This finding should be interpreted within the participants’ family context. In the present study, most participants were married and lived with family members, which may partly explain the prominent family involvement in daily stoma management and decision-making. In the context of Chinese family-oriented culture, family members often provide practical assistance and emotional support to elderly adults living with chronic conditions[25]. Therefore, with patients’ informed consent, appropriately involving family members in shared decision-making and health education processes may help improve decision quality and support long-term self-management[26]. In addition, healthcare professionals should provide individualized decision support by assessing patients’ information needs and health literacy levels, delivering structured and accessible education, and offering continuous guidance to facilitate daily self-management decisions[27].*

Previous studies have confirmed that patients with stomas frequently face alterations in body image and *psychological responses akin to a grief reaction[28]. Building on this, our findings further reveal that psychological adaptation is characterized by significant long-term, non-linear features[4,29]; even long after surgery, patients may continue to experience recurrent emotional fluctuations and identity conflicts.* From a theoretical perspective, this process resonates with frameworks such as "meaning reconstruction," *suggesting that patients do not simply return to a pre-illness state but, through continuous adjustment and adaptation, gradually construct a “new normal”* that incorporates the presence of the stoma[30]. *This psychological reconstruction may represent an important prerequisite for sustained self-management[31]. As patients gradually accepted the stoma and reconstructed their identity, they became more willing to engage in daily stoma care, seek solutions to complications, and integrate stoma management into their routines.* Based on these findings, clinical practice should prioritize both the continuity of psychological support and the recognition of *phase-specific patient needs[32]. Routine assessment of body image concerns, emotional distress, and adaptation difficulties during follow-up may help identify patients requiring additional psychological support[33]. During the early postoperative period, the focus should be on addressing patients’ body image distress and sense of loss of control, and on providing targeted psychological counseling.* In the long-term follow-up phase, it is essential to identify patients who remain in a state of psychological conflict and, through interventions such as narrative therapy and peer support groups, *facilitate meaning-making to enhance overall adaptation[34,35].*

*Our findings indicate that elderly patients living with a permanent stoma gradually developed psychological resilience through continuous self-adjustment and lived experience. The prominence of resilience in this study may be related to the cumulative life experiences of elderly adults[36]. Throughout their lives, elderly individuals may have encountered previous transitions, losses, and health-related challenges, which can serve as psychological resources for coping with the irreversible changes associated with permanent enterostomy[37]. Previous studies have often conceptualized resilience as an individual trait or a relatively stable personal resource that enables one to withstand adversity and maintain functioning[38]. However, our findings extend this perspective by suggesting that resilience among elderly patients with permanent stomas is not merely a pre-existing attribute but is gradually reconstructed through lived experience, continuous self-adjustment, and engagement in self-management practices. This interpretation aligns with contemporary resilience theories that emphasize resilience as a dynamic, developmental process shaped by person–environment interactions[39,40]. In this study, patients did not achieve effective self-management immediately after stoma formation. Instead, they gradually accumulated practical skills and confidence through repeated attempts, problem-solving, and adjustment in daily care routines. As patients became more familiar with stoma management, they shifted their attention from physical limitations toward maintaining daily activities, family relationships, and emotional well-being[41]. This process suggests that resilience functioned not only as a psychological coping resource but also as a facilitating factor supporting sustained self-management behaviors[42].While previous studies have largely focused on the psychological burden and negative experiences of stoma care[43], our findings highlight the adaptive capabilities of elderly patients and suggest that their accumulated life experiences may serve as valuable resources for reconstructing meaning, accepting bodily changes, and developing long-term self-management strategies[44]. These findings imply that interventions for elderly patients with permanent enterostomy should not focus solely on correcting deficits but should also incorporate a strength-based approach that recognizes patients’ existing abilities and life experiences. In clinical practice, healthcare professionals may encourage patients to reflect on successful self-management experiences, identify personal coping strategies, and participate in peer-support activities to enhance self-efficacy and promote sustained self-management behaviors[45,46].*

# **★ 6. Researcher/Interviewer Positionality**

**Reviewer’s Comment:
The researcher’s/interviewer’s positionality (including professional background) is not addressed. The dynamics of a professor or nurse interviewing elderly participants merit critical reflection.**

**➦ Response:**

Thank you for raising this important issue regarding reflexivity and researcher positionality. We agree that the interviewer’s professional background and relationship with participants may influence the interview process and interpretation of data.

In the revised manuscript, we have added information regarding the interviewer’s professional background and qualitative research experience. We clarified that the interviewer was a healthcare professional with clinical experience related to gastrointestinal surgery and stoma care. Although this background facilitated communication and understanding of participants’ experiences, we also recognized the potential influence of professional assumptions.

To minimize potential bias, the interviewer maintained a reflective attitude throughout the research process, used open-ended questions, encouraged participants to describe their own experiences, and discussed coding and interpretation with other members of the research team.

**Revised content in the manuscript:**
Additional information regarding researcher positionality and reflexivity has been added to the Methods section.

**2 Methods**

**2.4 Data collection**

*Participants were recruited through recruitment posters posted at an ostomy outpatient clinic using purposive sampling. This approach was chosen to obtain rich and diverse accounts of self-management experiences among elderly patients living with permanent enterostomy, with consideration of variations in stoma duration and individual adaptation experiences. All interviews were conducted by the first author, a male nursing graduate student with clinical experience in gastrointestinal surgery and training in qualitative research methods, including qualitative interviewing and data analysis. The interviewer had no prior therapeutic relationship with the participants, and participation or refusal did not affect their medical care. Before each interview, the researcher explained the study purpose, procedures, voluntary participation, and confidentiality principles, and written informed consent was obtained before audio recording. Face-to-face semi-structured interviews were conducted in a private demonstration room at the ostomy outpatient clinic using an interview guide. Open-ended questions and probing techniques were used to encourage participants to describe their experiences in depth, and field notes were recorded to capture contextual information and non-verbal expressions. Each interview lasted approximately 20–40 minutes, depending on participants’ willingness and the richness of their experiences. Although each participant was interviewed once, the semi-structured interview approach allowed participants to retrospectively describe their experiences from stoma formation to the present, including changes in self-management behaviors, challenges encountered, and adaptation processes.and all participants were interviewed once. Audio recordings were transcribed verbatim and checked for accuracy after each interview. Data collection continued until saturation was achieved; after the 10th interview, no substantially new codes emerged, and three additional interviews were conducted for confirmation. After the 13th interview, no new codes or themes were identified, and data collection was terminated.*

**2.7 Rigour and reflexivity**

*To enhance the methodological rigor of this qualitative study, the four criteria proposed by Lincoln and Guba, including credibility, dependability, confirmability, and transferability, were considered throughout the research process. Credibility was enhanced through purposive sampling to recruit participants with diverse experiences of living with a permanent enterostomy. Two researchers independently coded the interview transcripts, and coding results were compared and discussed within the research team until consensus was reached. To ensure dependability, detailed records of participant recruitment, data collection, and data analysis procedures were maintained to establish an audit trail. All transcripts were repeatedly reviewed and compared with the original audio recordings to ensure accuracy. To enhance confirmability, reflexive practices were incorporated throughout data analysis. The interviewer maintained reflexive notes documenting personal assumptions, reflections, and methodological decisions during data collection and interpretation. To minimize researcher bias during coding and interpretation, two researchers independently coded the interview transcripts, and coding results were compared and discussed within the research team until consensus was reached. Regular team discussions were conducted to identify potential biases and ensure that themes were grounded in participants’ narratives rather than researchers’ preconceptions. To enhance transferability, detailed descriptions of the research setting, participant characteristics, and sampling criteria were provided to allow readers to assess the applicability of the findings to other contexts. Data collection continued until data saturation was achieved. Data saturation was assessed throughout the data collection and analysis process. After the 10th interview, no substantially new codes were identified from the interview transcripts. However, to confirm the stability and comprehensiveness of the emerging findings, three additional interviews were conducted. After the 13th interview, no new codes or themes emerged, and the research team determined that sufficient information had been obtained to answer the research question. Therefore, participant recruitment and data collection were terminated.*

*This qualitative study was reported according to the Consolidated Criteria for Reporting Qualitative Research (COREQ) checklist, which includes 32 items covering the research team and reflexivity, study design, and data analysis and reporting. The completed COREQ checklist is provided as Supporting Information (S2 Table).*
