# Supplementary material for: Experiences of self-management behaviors during adaptation to permanent enterostomy among older patients: A qualitative study
Source: PLoS One. 2026 Oct 29;21(10):e0360643. doi: 10.1371/journal.pone.0360643 (PMC13718964; doi:10.1371/journal.pone.0360643)
Supplement: Supplementary file 6 [file pone.0360643.s007.docx]

**Response to Reviewers**

**Manuscript ID:PONE-D-26-23135R1**

**Title:Experiences of Self-Management Behaviors During Adaptation to Permanent Enterostomy Among Older Patients: A Qualitative Study**

Dear Dr. Jamshed Akhtar and Reviewers,

We sincerely appreciate you and the reviewers for the careful evaluation of our manuscript entitled “Experiences of Self-Management Behaviors During Adaptation to Permanent Enterostomy Among Elderly Patients: A Qualitative Study.” We are very grateful for the valuable and constructive comments provided throughout the review process, which have greatly helped us improve the methodological rigor, transparency, and overall quality of our manuscript.

We have carefully considered all comments and suggestions and have revised the manuscript accordingly. A detailed point-by-point response to each comment is provided below. All revisions have been highlighted in the revised manuscript using Track Changes, and a clean version of the revised manuscript has also been submitted for your convenience.

We sincerely thank the Academic Editor and all reviewers for their time, expertise, and thoughtful guidance. Their comments and suggestions have substantially strengthened the clarity, methodological rigor, and presentation of our study. We hope that the revised manuscript adequately addresses all concerns and meets the standards for publication in PLOS ONE.

Sincerely,

Fang Wu (Corresponding Author)

On behalf of all co-authors

**Section1. Response to Editor ( Page1-2 )**

**Section2. Response to Reviewer #1 ( Page 2)**

**Section 3. Response to Reviewer #3 ( Page 2-33)**

**Section 4. Response to Reviewer #4 ( Page 34)**

- **Section1. Response to Editor:**

We sincerely thank the Academic Editor and the PLOS ONE editorial team for the careful consideration of our manuscript and for providing us with the opportunity to revise our work.

We carefully considered all comments and recommendations provided during the review process and have revised the manuscript accordingly. As no additional editor-specific comments were provided in this revision round, we have addressed the concerns raised by the reviewers in detail and made corresponding revisions throughout the manuscript.

In particular, we have revised the manuscript to improve the clarity and focus of the Introduction and Discussion, clarify the methodological procedures regarding data saturation, thematic depth, coding, and reflexivity, distinguish self-management from related concepts such as psychological adaptation, resilience, and self-efficacy, strengthen the description of study limitations, and ensure that the interpretation and conclusions are appropriately aligned with the single retrospective interview design.

All changes have been highlighted in the revised manuscript using Track Changes, and a clean version of the revised manuscript has also been submitted. A detailed point-by-point response to each reviewer comment is provided in the following sections.

We sincerely appreciate the Academic Editor and reviewers for their thoughtful and constructive feedback, which has helped us substantially improve the methodological rigor, clarity, and overall quality of the manuscript.

- **Section2. Response to Reviewer #1:**

We sincerely appreciate your careful evaluation of our revised manuscript and your positive assessment of the changes made in response to the previous round of review. We are grateful for your recognition that our comments have been adequately addressed and that the revised manuscript is now improved and suitable for further consideration. Your thoughtful feedback and constructive guidance throughout the review process have been invaluable in strengthening the clarity, methodological rigor, and overall quality of our study. We sincerely thank you for your time, expertise, and careful review of our work.

- **Section3. Response to Reviewer #3:**

★ **1. Introduction: Too Long and Insufficiently Focused**

**Reviewer’s Comment:**

**The Introduction is too long and should be made more focused and concise.**

**➦Response:**

Thank you for this valuable comment. We agree that the Introduction in the previous version contained some redundant background information and could be presented more concisely. We have therefore substantially revised and streamlined the Introduction to improve its focus and logical flow.

Specifically, we removed a separate paragraph describing the general long-term consequences of living with a permanent stoma and eliminated peripheral details that were not essential to the study rationale, such as specific findings on disease-related knowledge. We also condensed the discussion of the challenges faced by older adults by integrating the relevant practical and informational difficulties into a more focused paragraph, while retaining their accumulated life experience and established coping strategies as potential personal resources.

In addition, the definitions of self-management and related concepts were streamlined while retaining the necessary distinctions among self-care, psychological adaptation, independence, and self-efficacy. The literature review and research gap were also reorganized to provide a clearer progression from existing evidence to the specific gap addressed by this study. In particular, we now emphasize that previous research has primarily examined self-management levels, difficulties, support needs, and related factors, whereas less attention has been given to how older adults with permanent stomas perceive and make sense of changes in self-management through their everyday experiences, particularly in relation to decision-making, problem-solving, repeated adjustment, and accumulated life experience. We further clarified the contribution of the present study by focusing on participants’ lived meanings and perceived changes in self-management using a descriptive phenomenological approach.

These revisions have substantially reduced redundancy and strengthened the focus of the Introduction while preserving the essential background, conceptual clarification, research gap, and rationale for the study. The corresponding changes are highlighted in the revised manuscript using Track Changes.

➦**Comparison before and after modification**

**Before modification**:Colorectal cancer (CRC) is one of the most common malignancies and poses a significant threat to human health because of its high incidence and mortality rates. According to Global Cancer Statistics 2022 released by the International Agency for Research on Cancer (IARC)[1], newly diagnosed cases of CRC accounted for 9.6% of all cancer cases, and CRC-related deaths constituted 9.3% of all cancer-related deaths. Ostomy surgery, a common intervention for CRC, involves creating an opening in the abdominal wall through which a segment of the intestine is exteriorized to serve as a substitute for the anus[2]. This procedure effectively alleviates symptoms, prevents disease progression, and extends survival[3].However, owing to factors such as disease advancement or the specific surgical approach employed, some patients require a stoma for an extended period—or even for life—resulting in a permanent intestinal ostomy.

Unlike temporary stomas, a permanent intestinal stoma implies that patients must contend over the long term with issues such as altered physiological function and restructuring of their lifestyle[4]. Postoperatively, patients need not only to cope with stoma-related complications and discomfort but also to continuously adapt their daily behaviors; consequently, their quality of life is affected to varying degrees[5].

Self-management is a multidimensional concept that encompasses the behaviors, cognitive processes, and emotional strategies individuals use to manage chronic health conditions and their consequences[6]. In the present study, self-management among elderly patients with a permanent enterostomy refers to the ongoing efforts to manage stoma care, prevent complications, seek information, make health-related decisions, adjust daily activities, and cope with the psychological and social changes of living with a stoma. Compared with self-care, which primarily emphasizes the performance of specific health-related tasks, self-management represents a broader construct integrating behavioral actions, cognitive regulation, and coping processes[7]. Other related concepts, such as psychological adaptation, independence, and self-efficacy, may influence or facilitate self-management but are not equivalent to it[6,8].

After ostomy surgery, the demands of self-management extend to every dimension of daily life, including dietary adjustments, stoma care, and social participation, and rely not only on relevant knowledge and skills but, more importantly, on an individual’s ongoing behavioral adaptation and decision-making across varying contexts[9,10]. However, with the widespread adoption of Enhanced Recovery After Surgery (ERAS) protocols, hospital stays have become progressively shorter; consequently, some patients have not yet fully developed self-management capabilities by the time of discharge. Furthermore, during the early postoperative period, most patients struggle to adapt quickly to the altered body image associated with the stoma, and their awareness of the need to assume self-management responsibilities proactively remains relatively limited[11]. Age-related changes such as slower cognitive processing, reduced reaction time, and altered learning capacity can increase the difficulty of mastering the knowledge and skills required for stoma care[12].Relevant studies indicate that the overall level of self-management among elderly stoma patients remains suboptimal, with only approximately 50.4% possessing adequate disease-related knowledge[13]. Furthermore, stoma care procedures are inherently complex and technically demanding; consequently, elderly patients may find it challenging to transition from dependent care to independent self-management within a short period[14].

Although previous studies have explored the self-management experiences and support needs of patients with stomas, existing research has focused primarily on challenges, adaptation difficulties, and supportive needs[15].Less attention has been paid to how elderly patients with permanent enterostomies experience the development of self-management behaviors through continuous adjustment and problem-solving during adaptation. Therefore, this study adopted a qualitative phenomenological approach to explore these experiences, aiming to inform individualized supportive interventions and improve long-term care strategies for this population.

**Changed:** Colorectal cancer (CRC) is one of the most common malignancies and poses a significant threat to human health because of its high incidence and mortality rates. According to Global Cancer Statistics 2022 released by the International Agency for Research on Cancer (IARC)[1], newly diagnosed cases of CRC accounted for 9.6% of all cancer cases, and CRC-related deaths constituted 9.3% of all cancer-related deaths. *In China, an estimated 517,106 new cases of CRC and 240,010 CRC-related deaths occurred in 2022, accounting for approximately 10.7% and 9.3% of all new cancer cases and cancer deaths, respectively[2].* *For selected patients with CRC, ostomy surgery may be required depending on disease characteristics and the surgical approach. It involves creating an opening in the abdominal wall through which a segment of the intestine is brought to the skin surface to divert fecal elimination[3]*. This procedure effectively alleviates symptoms, prevents disease progression, and extends survival[4].However, owing to factors such as disease advancement or the specific surgical approach employed, some patients require a stoma for an extended period—or even for life—resulting in a permanent stoma.

Self-management is a multidimensional concept that encompasses the behaviors, cognitive processes, and emotional strategies individuals use to manage chronic health conditions and their consequences[5]. *In the present study, self-management among older patients with a permanent stoma refers to the ongoing efforts to manage stoma care, prevent complications, seek information, make health-related decisions, adjust daily activities, and cope with the psychological and social changes of living with a stoma. Self-care primarily refers to specific health-related care activities, whereas self-management encompasses broader behavioral, cognitive, emotional, and social processes involved in managing a long-term condition[6,7]. Psychological adaptation refers to adjustment to the physical and psychosocial changes associated with illness, independence reflects the extent to which these activities can be performed without assistance, and self-efficacy refers to individuals’ confidence in their ability to perform them[8–10]. These concepts are related to, but distinct from, self-management[5,11].*

After ostomy surgery, the demands of self-management extend to every dimension of daily life, including dietary adjustments, stoma care, and social participation, and rely not only on relevant knowledge and skills but, more importantly, on an individual’s ongoing behavioral adaptation and decision-making across varying contexts[12,13]. However, with the widespread adoption of Enhanced Recovery After Surgery (ERAS) protocols, hospital stays have become progressively shorter; consequently, some patients have not yet fully developed self-management capabilities by the time of discharge. Furthermore, during the early postoperative period, most patients struggle to adapt quickly to the altered body image associated with the stoma, and their awareness of the need to assume self-management responsibilities proactively remains relatively limited[14]. *These challenges may be particularly relevant for older adults, who may experience greater difficulty acquiring new stoma-care knowledge and skills, while also drawing on accumulated life experience and established coping strategies as potential personal resources for managing long-term health changes[15,16].* Furthermore, stoma care procedures are inherently complex and technically demanding; consequently, older patients may find it challenging to transition from dependent care to independent self-management within a short period[17].

Previous studies have examined enterostomy self-management in relation to self-care, quality of life, psychological adaptation, support needs, and associated factors[12,18].. *However, less is known about how older adults with permanent stomas themselves perceive and make sense of changes in self-management during adaptation, particularly in relation to decision-making, problem-solving, repeated adjustment, and accumulated life experience[19].* *While previous research has largely focused on self-management levels, difficulties, and support needs, the present study further explores the lived meanings and perceived changes underlying these experiences from the participants’ own perspectives.* Therefore, this study adopted a descriptive phenomenological approach to explore older adults’ lived experiences and perceived changes in self-management during adaptation to a permanent stoma.

★ **2. Terminology: “Elderly Patients”**

**Reviewer’s Comment:**

**The term “elderly patients” should be replaced consistently with “older adults” or “older patients.”**

**➦Response**

Thank you for this helpful comment. We have revised the manuscript to replace the term “elderly patients” with “older patients” as appropriate and have ensured consistent terminology throughout the manuscript, including the title, Introduction, Methods, Results, Discussion, and Conclusion.

★ **3. Terminology Consistency: “Ostomy,” “Stoma,” and Related Terms**

**Reviewer’s Comment:**

**The terms “ostomy,” “stoma,” “enterostomy,” and “intestinal ostomy” should be used consistently throughout the manuscript.**

**➦Response**

Thank you for this helpful comment. We have carefully reviewed the terminology used throughout the manuscript and revised it for consistency. We use “stoma” as the primary term when referring to the surgically created opening and the patient’s ongoing stoma-related condition, while “ostomy surgery” is used when referring specifically to the surgical procedure. The terms “enterostomy” and “intestinal ostomy” have been standardized or replaced where appropriate to avoid unnecessary variation. These terminology revisions have been applied consistently throughout the title, Introduction, Methods, Results, Discussion, and Conclusion.

**★ 4. Clarification of the Indication for Stoma Formation**

**Reviewer’s Comment:**

**The statement “ostomy surgery is a common intervention for CRC” may be misleading because not all patients with colorectal cancer require a stoma. Please clarify that stoma formation may be required in selected patients.**

**➦Response**

Thank you for this important clarification. We agree that the previous wording could incorrectly imply that stoma formation is required for all patients with colorectal cancer. We have revised this statement to clarify that stoma formation may be required in selected patients with CRC, depending on disease characteristics and the surgical approach. The revised text reads: “For selected patients with CRC, ostomy surgery may be required depending on disease characteristics and the surgical approach.” This revision improves the clinical accuracy and avoids overgeneralization.

➦**Comparison before and after modification**

**Before modification**:Ostomy surgery, a common intervention for CRC, involves creating an opening in the abdominal wall through which a segment of the intestine is exteriorized to serve as a substitute for the anus[2]. This procedure effectively alleviates symptoms, prevents disease progression, and extends survival[3].

**Changed**:*For selected patients with CRC, ostomy surgery may be required depending on disease characteristics and the surgical approach. It involves creating an opening in the abdominal wall through which a segment of the intestine is brought to the skin surface to divert fecal elimination[3].* This procedure effectively alleviates symptoms, prevents disease progression, and extends survival[4].

**★ 5. Anatomical Description of Stoma**

**Reviewer’s Comment:**

**The description “a substitute for the anus” is anatomically simplistic and potentially inaccurate. A more appropriate description is that a segment of the intestine is brought through the abdominal wall to divert or facilitate fecal elimination.**

**➦Response**

Thank you for this important clarification. We agree that the previous description, “a substitute for the anus,” was anatomically simplistic and potentially misleading. We have therefore revised the description to provide a more accurate explanation of stoma formation. The revised text now states: *“It involves creating an opening in the abdominal wall through which a segment of the intestine is brought to the skin surface to divert fecal elimination.”* This revision improves the anatomical accuracy and clarity of the manuscript.

★ **6. National or Regional Data**

**Reviewer’s Comment:**

**National or regional data should be included to establish the relevance of the study in the country where the research was conducted.**

**➦Response**

Thank you for this valuable suggestion. We agree that including national epidemiological data would better establish the relevance and context of the present study. We have therefore added recent colorectal cancer incidence and mortality data for China to the Introduction. *Specifically, we now report that an estimated 517,106 new CRC cases and 240,010 CRC-related deaths occurred in China in 2022, accounting for approximately 10.7% and 9.3% of all new cancer cases and cancer deaths, respectively[2].* This addition provides a clearer epidemiological context for the study and highlights the relevance of CRC and permanent stoma-related self-management in the Chinese setting.

➦**Comparison before and after modification**

**Before modification**:According to Global Cancer Statistics 2022 released by the International Agency for Research on Cancer (IARC)[1], newly diagnosed cases of CRC accounted for 9.6% of all cancer cases, and CRC-related deaths constituted 9.3% of all cancer-related deaths.

**Changed**:*In China, an estimated 517,106 new cases of CRC and 240,010 CRC-related deaths occurred in 2022, accounting for approximately 10.7% and 9.3% of all new cancer cases and cancer deaths, respectively[2].*

**★ 7. Conceptual Distinctions**

**Reviewer’s Comment:**

**The distinction between self-management, self-care, psychological adaptation, independence, and self-efficacy should be presented more concisely.**

➦**Comparison before and after modification**

**➦Response**

Thank you for this helpful comment. We agree that the previous version provided overly detailed explanations of these related concepts. We have therefore condensed this section by retaining a focused definition of self-management and briefly distinguishing the related concepts. Specifically, self-care is described as specific care activities, psychological adaptation as adjustment to illness-related changes, independence as performing activities without assistance, and self-efficacy as confidence in performing self-management behaviors. We also clarified that these concepts are related to, but distinct from, self-management. This revision reduces redundancy and improves the conciseness and readability of the Introduction.

➦**Comparison before and after modification**

**Before modification**:Self-management is a multidimensional concept that encompasses the behaviors, cognitive processes, and emotional strategies individuals use to manage chronic health conditions and their consequences[6]. In the present study, self-management among elderly patients with a permanent enterostomy refers to the ongoing efforts to manage stoma care, prevent complications, seek information, make health-related decisions, adjust daily activities, and cope with the psychological and social changes of living with a stoma. Compared with self-care, which primarily emphasizes the performance of specific health-related tasks, self-management represents a broader construct integrating behavioral actions, cognitive regulation, and coping processes[7]. Other related concepts, such as psychological adaptation, independence, and self-efficacy, may influence or facilitate self-management but are not equivalent to it[6,8].

**Changed**:Self-management is a multidimensional concept that encompasses the behaviors, cognitive processes, and emotional strategies individuals use to manage chronic health conditions and their consequences[5]. In the present study, self-management among older patients with a permanent stoma refers to the ongoing efforts to manage stoma care, prevent complications, seek information, make health-related decisions, adjust daily activities, and cope with the psychological and social changes of living with a stoma. *Self-care refers to specific care activities, psychological adaptation to adjustment to illness-related changes, independence to the extent of performing activities without assistance, and self-efficacy to confidence in performing self-management behaviors[6–8]. These concepts are related to, but distinct from, self-management[5,9].*

**★ 8. Strengths of Older Adults**

**Reviewer’s Comment:**

**The Introduction should acknowledge the strengths and potential resources of older adults.**

**➦Response**

Thank you for this valuable suggestion. We agree that the previous version focused predominantly on the challenges faced by older adults and did not sufficiently acknowledge their potential strengths. We have therefore added a statement highlighting that, despite potential difficulties in acquiring new stoma-care knowledge and skills, older adults may draw on accumulated life experience and established coping strategies as personal resources for managing long-term health changes. This addition provides a more balanced perspective on the challenges and strengths of older adults and better aligns the Introduction with the findings of the present study.

➦**Comparison before and after modification**

**Before modification**:After ostomy surgery, the demands of self-management extend to every dimension of daily life, including dietary adjustments, stoma care, and social participation, and rely not only on relevant knowledge and skills but, more importantly, on an individual’s ongoing behavioral adaptation and decision-making across varying contexts[9,10]. However, with the widespread adoption of Enhanced Recovery After Surgery (ERAS) protocols, hospital stays have become progressively shorter; consequently, some patients have not yet fully developed self-management capabilities by the time of discharge. Furthermore, during the early postoperative period, most patients struggle to adapt quickly to the altered body image associated with the stoma, and their awareness of the need to assume self-management responsibilities proactively remains relatively limited[11]. Age-related changes such as slower cognitive processing, reduced reaction time, and altered learning capacity can increase the difficulty of mastering the knowledge and skills required for stoma care[12].Relevant studies indicate that the overall level of self-management among elderly stoma patients remains suboptimal, with only approximately 50.4% possessing adequate disease-related knowledge[13]. Furthermore, stoma care procedures are inherently complex and technically demanding; consequently, elderly patients may find it challenging to transition from dependent care to independent self-management within a short period[14].

**Changed**:After ostomy surgery, the demands of self-management extend to every dimension of daily life, including dietary adjustments, stoma care, and social participation, and rely not only on relevant knowledge and skills but, more importantly, on an individual’s ongoing behavioral adaptation and decision-making across varying contexts[10,11]. However, with the widespread adoption of Enhanced Recovery After Surgery (ERAS) protocols, hospital stays have become progressively shorter; consequently, some patients have not yet fully developed self-management capabilities by the time of discharge. Furthermore, during the early postoperative period, most patients struggle to adapt quickly to the altered body image associated with the stoma, and their awareness of the need to assume self-management responsibilities proactively remains relatively limited[12]. *These challenges may be particularly relevant for older adults, who may experience greater difficulty acquiring new stoma-care knowledge and skills, while also drawing on accumulated life experience and established coping strategies as potential personal resources for managing long-term health changes[13,14].* Furthermore, stoma care procedures are inherently complex and technically demanding; consequently, older patients may find it challenging to transition from dependent care to independent self-management within a short period[15].

**★ 9. Synthesis of Evidence from Multiple Studies**

**Reviewer’s Comment:**

**Evidence from multiple studies should be synthesized rather than relying heavily on isolated findings.**

**➦Response**

Thank you for this important suggestion. We agree that the previous version relied too heavily on individual study findings and did not sufficiently synthesize the existing evidence. We have therefore revised the final paragraph of the Introduction to provide a more integrated summary of previous research. Specifically, rather than discussing individual studies or distinguishing findings by study design, we now synthesize the existing literature around several major dimensions of enterostomy self-management, including self-care, quality of life, psychological adaptation, support needs, and associated factors[10,16]. We then narrow this broader evidence base to the specific gap addressed by the present study, namely, how older adults with permanent stomas perceive and make sense of changes in self-management in relation to decision-making, problem-solving, repeated adjustment, and accumulated life experience[17]. This revision reduces reliance on isolated findings and provides a more coherent transition from the existing evidence to the rationale and focus of the present study.

➦**Comparison before and after modification**

**Before modification**:Although previous studies have explored the self-management experiences and support needs of patients with stomas, existing research has focused primarily on challenges, adaptation difficulties, and supportive needs[15].Less attention has been paid to how elderly patients with permanent enterostomies experience the development of self-management behaviors through continuous adjustment and problem-solving during adaptation. Therefore, this study adopted a qualitative phenomenological approach to explore these experiences, aiming to inform individualized supportive interventions and improve long-term care strategies for this population.

**Changed**:*Previous studies have examined enterostomy self-management in relation to self-care, quality of life, psychological adaptation, support needs, and associated factors[10,16]. However, less is known about how older adults with permanent stomas themselves perceive and make sense of changes in self-management during adaptation, particularly in relation to decision-making, problem-solving, repeated adjustment, and accumulated life experience[17]. While previous research has largely focused on self-management levels, difficulties, and support needs, the present study further explores the lived meanings and perceived changes underlying these experiences from the participants’ own perspectives.* Therefore, this study adopted a descriptive phenomenological approach to explore older adults’ lived experiences and perceived changes in self-management during adaptation to a permanent stoma.

**★ 10. Narrowing and Justifying the Research Gap**

**Reviewer’s Comment:**

**The research gap should be narrowed from general statements to a precise and well-justified problem.**

**➦Response**

Thank you for this important comment. We agree that the research gap in the previous version was too broad and needed to be more precisely defined. We have therefore revised the final paragraph of the Introduction to narrow the gap from general concerns about stoma self-management to a specific experiential problem.

Specifically, we first synthesized *previous evidence on enterostomy self-management across several dimensions, including self-care, quality of life, psychological adaptation, support needs, and associated factors[10,16]*. We then identified a more specific gap: *limited understanding of how older adults with permanent stomas themselves perceive and make sense of changes in self-management during adaptation, particularly in relation to decision-making, problem-solving, repeated adjustment, and accumulated life experience[17]*. This gap is directly relevant to the present study because these experiences may not be fully captured by measures of self-management level, difficulties, or support needs alone. We consequently clarified the rationale for adopting a descriptive phenomenological approach to explore participants’ lived experiences and perceived changes in self-management during adaptation to a permanent stoma.

These revisions have narrowed the research question from a general concern with self-management to a specific, experience-based problem that can be appropriately addressed through qualitative phenomenological inquiry.

**★ 11. Clarification of the Novel Contribution**

**Reviewer’s Comment:**

**The potentially novel contribution appears to concern how self-management develops through accumulated experience, repeated adjustment, decision-making, and problem-solving. This contribution should be stated more explicitly.**

**➦Response**

Thank you for highlighting this important aspect of our study. We agree that the potential novel contribution of the present study should be stated more explicitly. We have therefore revised the final part of the Introduction to clarify that, beyond describing self-management difficulties or support needs, this study focuses on how older adults with permanent stomas perceive and make sense of changes in self-management through accumulated experience, repeated adjustment, decision-making, and problem-solving during adaptation.

To avoid implying that self-management development was directly observed longitudinally, we have framed the contribution in terms of participants’ retrospective accounts and perceived changes in self-management. This wording is consistent with our descriptive phenomenological design and single retrospective interview, while making the distinctive contribution of the study more explicit.

**★ 12. Alignment of the Study Objective with the Qualitative Approach**

**Reviewer’s Comment:**

**The objective should be clarified to justify why descriptive phenomenology and Colaizzi’s method were selected instead of grounded theory or a longitudinal qualitative design, particularly if the objective is to understand a process of behavioural development.**

**➦Response**

Thank you for this important methodological comment. We agree that the wording of the original objective could imply that the study aimed to develop or directly observe a process of behavioural development, which would be more consistent with a grounded theory or longitudinal qualitative approach. We have therefore revised the study objective to focus on the lived experiences and meanings of self-management and participants’ perceived changes in self-management during adaptation, rather than on constructing a formal developmental process.

Accordingly, we clarified the rationale for the methodological approach. A descriptive phenomenological approach was selected to explore participants’ lived experiences and the meanings they attributed to self-management, while Colaizzi’s method was used to systematically analyze these lived experiences and identify their essential meanings. Grounded theory was not selected because the aim was not to generate a formal explanatory theory or process model, and a longitudinal qualitative design was not used because data were collected through a single retrospective interview rather than repeated interviews across different stages of adaptation.

These revisions align the study objective, research design, and analytic approach more closely and clarify that the findings represent participants’ retrospective perceptions of changes in self-management rather than a directly observed developmental process.

➦**Comparison before and after modification**

**Before modification:**2.1 Study design

This study employs a phenomenological research approach to explore the experiences and needs associated with the self-management process from the patient's perspective. This approach was selected because it focuses on understanding individuals’ subjective experiences and the meanings they attribute to a particular phenomenon, which was consistent with the aim of this study to explore patients’ experiences of self-management during long-term adaptation after enterostomy. Through semi-structured in-depth interviews, the study investigates the self-management behaviors of elderly patients with permanent intestinal stomas during their long-term adaptation process. The research was conducted from March 25 to May 5, 2026, within the Department of Gastrointestinal Surgery and the Stoma Clinic of a tertiary hospital in the Inner Mongolia Autonomous Region, China. The hospital provides specialized care for patients with colorectal diseases, and the Stoma Clinic offers postoperative follow-up, stoma care education, and complication management services for patients with enterostomies. The sample size was determined based on the principle of data saturation; specifically, when interviews ceased to yield new themes or information, a small number of additional participants were recruited to verify data saturation, and data collection was terminated once the sufficiency of information was confirmed[16].

### Changed:**2.1 Study design**

This study employed a *descriptive phenomenological* approach to *explore* *the lived experiences and meanings of self-management among older adults with permanent stomas during adaptation.* *This approach was selected to understand participants’ subjective experiences and the meanings they attributed to self-management in everyday life. Because each participant was interviewed once, the study focused on participants’ retrospective accounts and perceptions of changes in self-management from stoma formation to the time of interview, rather than directly observing changes across different stages of adaptation.* Through semi-structured in-depth interviews, the study investigates the self-management behaviors of *older* patients with permanent intestinal stomas during their long-term adaptation process. The research was conducted from March 25 to May 5, 2026, within the Department of Gastrointestinal Surgery and the Stoma Clinic of a tertiary hospital in the Inner Mongolia Autonomous Region, China. The hospital provides specialized care for patients with colorectal diseases, and the Stoma Clinic offers postoperative follow-up, stoma care education, and complication management services for patients with enterostomies.

**★ 13. Alignment of the Objective with Phenomenological Research**

**Reviewer’s Comment:**

**If the study is phenomenological, the objective should focus on the lived experience and meaning of developing self-management rather than attempting to construct a formal process model.**

**➦Response**

Thank you for this important methodological comment. We agree that the original objective could be interpreted as attempting to describe or construct a formal process model of self-management development. We have therefore revised the objective to focus on the lived experiences and meanings of self-management among older adults with permanent stomas, with particular attention to participants’ perceived changes in self-management during adaptation.

This revision places the emphasis on how participants experienced and made sense of self-management rather than on constructing a chronological or causal model of behavioural development. We also clarified in the Methods that the study employed a descriptive phenomenological approach to explore participants’ subjective experiences and meanings, and that the analysis focused on these lived experiences rather than on developing a formal process model. Given the single retrospective interview, the findings are presented as participants’ perceived changes in self-management rather than as a directly observed developmental trajectory.

**★ 14. Critical Synthesis of Previous Studies**

**Reviewer’s Comment:**

**Provide a more critical synthesis of previous studies.**

**➦Response**

Thank you for this valuable suggestion. We agree that the previous version primarily summarized existing research without sufficiently highlighting its limitations and implications for the present study. We have therefore revised the literature synthesis to move beyond a descriptive summary and provide a more critical interpretation of the existing evidence.

Specifically, we now synthesize previous research across *self-care, quality of life, psychological adaptation, support needs, and factors associated with enterostomy self-management[10,16]*, *while also highlighting that existing studies have largely focused on self-management levels, reported difficulties, and support needs. This evidence has established the multidimensional nature of enterostomy self-management but provides comparatively limited insight into how older adults with permanent stomas themselves perceive and make sense of changes in self-management during adaptation, particularly in relation to decision-making, problem-solving, repeated adjustment, and accumulated life experience[17]*. We use this limitation of the existing evidence to more clearly justify the specific research gap and the need for the present descriptive phenomenological study.

These revisions provide a more analytical synthesis of the literature by identifying both what is known and what remains insufficiently understood, thereby strengthening the rationale for the present study.

**★ 15. Clarification of the Phenomenological Approach**

**Reviewer’s Comment:**

**Descriptive phenomenology, interpretive phenomenology, or another phenomenological approach?**

**➦Response**

Thank you for this important methodological clarification. We have explicitly clarified that the present study employed a **descriptive phenomenological approach**. This approach was selected because the aim of the study was to describe older adults’ lived experiences and the meanings they attributed to self-management during adaptation, rather than to develop an interpretive theory or a formal explanatory process model.

Accordingly, we have revised the **Study Design** section to explicitly identify the study as descriptive phenomenology and to explain the rationale for this choice. We have also clarified that **Colaizzi’s seven-step** **phenomenological method** was used for data analysis, consistent with the descriptive phenomenological orientation of the study. These revisions ensure greater consistency among the study objective, phenomenological approach, and analytic method.

**★ 16. Limitation of the Single Retrospective Interview**

**Reviewer’s Comment:**

**A single retrospective interview cannot directly demonstrate how self-management behaviours changed over time.**

**➦Response**

Thank you for this important clarification. We agree that a single retrospective interview cannot directly demonstrate changes in self-management behaviors across different stages of adaptation. We have therefore revised the manuscript to clearly distinguish between participants’ retrospective accounts and perceived changes and the direct observation of longitudinal behavioral change.

Specifically, we have clarified in the **Study Design** and **Data Collection** sections that each participant was interviewed once and that the study focused on participants’ retrospective descriptions of their experiences from stoma formation to the time of interview, including their perceptions of changes in self-management. We have also revised the **Discussion, Conclusion, and Limitations** to avoid implying that a dynamic developmental process was directly observed. The Limitations section now explicitly acknowledges that the single retrospective interview captures participants’ perceived changes rather than directly observed changes over time and notes that longitudinal qualitative studies using repeated interviews at multiple time points could provide richer insights into how self-management experiences develop and change.

➦**Comparison before and after modification**

**Before modification:**2.4 Data collection

Participants were recruited through recruitment posters posted at an ostomy outpatient clinic using purposive sampling. This approach was chosen to obtain rich and diverse accounts of self-management experiences among elderly patients living with permanent enterostomy, with consideration of variations in stoma duration and individual adaptation experiences. All interviews were conducted by the first author, a male nursing graduate student with clinical experience in gastrointestinal surgery and training in qualitative research methods, including qualitative interviewing and data analysis. The interviewer had no prior therapeutic relationship with the participants, and participation or refusal did not affect their medical care. Before each interview, the researcher explained the study purpose, procedures, voluntary participation, and confidentiality principles, and written informed consent was obtained before audio recording. Face-to-face semi-structured interviews were conducted in a private demonstration room at the ostomy outpatient clinic using an interview guide. Open-ended questions and probing techniques were used to encourage participants to describe their experiences in depth, and field notes were recorded to capture contextual information and non-verbal expressions. Each interview lasted approximately 20–40 minutes, depending on participants’ willingness and the richness of their experiences. Although each participant was interviewed once, the semi-structured interview approach allowed participants to retrospectively describe their experiences from stoma formation to the present, including changes in self-management behaviors, challenges encountered, and adaptation processes.and all participants were interviewed once. Audio recordings were transcribed verbatim and checked for accuracy after each interview. Data collection continued until saturation was achieved; after the 10th interview, no substantially new codes emerged, and three additional interviews were conducted for confirmation. After the 13th interview, no new codes or themes were identified, and data collection was terminated.

**Changed:2.4 Data collection**

Participants were recruited through posters displayed at the stoma outpatient clinic. *During the recruitment period, 18 individuals expressed interest in the study and were screened for eligibility. Of these, 15 met the inclusion criteria, 1 declined participation, and 1 was excluded because of a physical condition that prevented participation in the interview.* A total of 13 participants ultimately provided written informed consent and completed the interview. Purposive sampling was used to obtain information-rich accounts of self-management among older adults with permanent stomas, with attention to variation in age, sex, time since stoma formation, stoma type, stoma-related complications, and living arrangement. *This approach was intended to capture diverse perspectives on self-management and adaptation rather than to achieve statistical representativeness or compare predefined postoperative stages.* All interviews were conducted by the first author, a male nursing graduate student with clinical experience in gastrointestinal surgery and training in qualitative research methods, including qualitative interviewing and data analysis. The interviewer had no prior therapeutic relationship with the participants, and participation or refusal did not affect their medical care. Before each interview, the researcher explained the study purpose, procedures, voluntary participation, and confidentiality principles, and written informed consent was obtained before audio recording. Face-to-face semi-structured interviews were conducted in a private demonstration room at the *stoma outpatient clinic* using an interview guide. Open-ended questions and probing techniques were used to encourage participants to describe their experiences in depth, and field notes were recorded to capture contextual information and non-verbal expressions. Each interview lasted approximately 20–40 minutes, depending on participants’ willingness and the richness of their experiences. *Although each participant was interviewed once, the semi-structured interview allowed participants to retrospectively describe their experiences from stoma formation to the time of interview, including their perceptions of changes in self-management, challenges encountered, and adaptation experiences.* Audio recordings were transcribed verbatim and checked for accuracy after each interview. Data collection continued until data saturation was reached. The first author reviewed the interview transcripts and emerging codes throughout the data collection process to monitor whether new information or codes continued to emerge. After the 10th interview, no substantially new codes were identified. Three additional interviews were therefore conducted to confirm the stability and comprehensiveness of the emerging findings. After the 13th interview, no new codes or themes emerged. *The first author presented the saturation assessment to the research team, and the team discussed and confirmed that sufficient information had been obtained to address the research question.* Data collection was then terminated.

**★ 17. Variation in Time Since Stoma Formation**

**Reviewer’s Comment:**

**The wide variation in time since stoma formation should be justified.**

**➦Response**

Thank you for this important comment. We agree that the variation in time since stoma formation should be explicitly justified. We have therefore clarified in the Methods that this variation was intentionally retained to capture a range of retrospective adaptation experiences and perspectives on self-management, rather than to compare predefined postoperative stages.

We further clarified that participants were not grouped or analyzed according to the duration since stoma formation. Instead, variation in duration was treated as a source of experiential diversity within the phenomenological sample. This approach was consistent with the aim of exploring participants’ lived experiences and perceived changes in self-management rather than constructing a stage-based or longitudinal model of behavioral developmen

➦**Comparison before and after modification**

**Before modification:2.2 Inclusion and Exclusion Criteria**

Although participants differed in time since stoma formation, this variation was considered valuable because the study aimed to explore diverse adaptation experiences rather than compare specific postoperative stages.

**Changed:2.2 Inclusion and Exclusion Criteria**

*Participants varied in the duration* *since stoma formation. This variation was intentionally retained to capture a range of retrospective adaptation experiences and perspectives on self-management rather than to compare predefined postoperative stages. Participants were therefore not grouped or analyzed according to stoma duration; instead, variation in duration was treated as a source of experiential diversity within the phenomenological sample.*

**★ 18. Purposive Sampling and Participant Recruitment**

**Reviewer’s Comment:**

**Please clarify how purposive sampling was implemented, how participant diversity was achieved, and how many individuals were screened, declined, or excluded.**

**➦Response**

Thank you for this important methodological clarification. We have revised the **Data Collection** section to provide a more detailed description of the purposive sampling and participant recruitment process.

Specifically, we now report the participant flow during recruitment: *18 individuals expressed interest and were screened for eligibility; 15 met the inclusion criteria; 1 declined participation; and 1 was excluded because of a physical condition that prevented participation, resulting in 13 participants who provided written informed consent and completed the interviews.* We also clarify that *purposive sampling was used to obtain information-rich accounts and to capture variation in age, sex, time since stoma formation, stoma type, stoma-related complications, and living arrangement. This approach was intended to obtain diverse perspectives on self-management and adaptation rather than to achieve statistical representativeness or compare predefined postoperative stages.*

These revisions clarify how purposive sampling was implemented, how participant diversity was considered, and how individuals were screened, declined, or excluded before the final sample was established.

**★ 19. Implementation of Colaizzi’s Phenomenological Method**

**Reviewer’s Comment:**

**The implementation of Colaizzi’s method is insufficiently described.**

**➦Response**

Thank you for this important methodological comment. We agree that the previous version did not provide sufficient detail on how Colaizzi’s phenomenological method was implemented. We have therefore substantially expanded the *Data Analysis section* to explicitly describe the analytic procedures corresponding to Colaizzi’s seven steps.

Specifically, we now describe how the researchers: *(1) repeatedly read the transcripts to gain an overall understanding of participants’ accounts; (2) identified and extracted significant statements relevant to self-management experiences; (3) formulated meanings while remaining grounded in participants’ original accounts; (4) organized related formulated meanings into clusters and developed preliminary themes; (5) constructed an exhaustive description of the phenomenon; (6) identified the fundamental structure by synthesizing the essential meanings across themes; and (7) compared the resulting themes and fundamental structure with the original transcripts to ensure consistency with participants’ accounts.*

We have also clarified the additional analytic procedures used alongside Colaizzi’s method, including the use of NVivo 11 Plus for data management, independent coding by two researchers, discussion of discrepancies until consensus was reached, and repeated comparison of emerging themes with the original transcripts. These revisions provide a more transparent and reproducible description of how Colaizzi’s method was applied in the present study.

➦**Comparison before and after modification**

**Before modification:2.6 Data analysis**

Following data collection, the personal information of the interviewees was anonymized and assigned numerical identifiers. Within 24 hours of the conclusion of each interview, the audio recordings were transcribed into text; simultaneously, non-verbal behaviors—such as interjections and pauses—were also transcribed alongside the verbal content. Upon completion of the initial transcription, the researcher repeatedly cross-referenced the audio recordings against the written transcripts to verify the accuracy of the transcribed data. The textual content was then imported into NVivo 11 PLUS, a qualitative data analysis software, for organization, coding, synthesis, and the extraction of key themes. This study employed Colaizzi’s phenomenological method of data analysis to organize and analyze the collected data[17].

**Changed:2.6 Data analysis**

Following data collection, the personal information of the interviewees was anonymized and assigned numerical identifiers. Within 24 hours of the conclusion of each interview, the audio recordings were transcribed into text; simultaneously, non-verbal behaviors—such as interjections and pauses—were also transcribed alongside the verbal content. Upon completion of the initial transcription, the researcher repeatedly cross-referenced the audio recordings against the written transcripts to verify the accuracy of the transcribed data. The textual content was then imported into NVivo 11 PLUS, a qualitative data analysis software, for organization, coding, synthesis, and the extraction of key themes. This study employed Colaizzi’s phenomenological method of data analysis to organize and analyze the collected data[18].*First, the researchers repeatedly read the transcripts to gain an overall understanding of participants’ accounts. Second, significant statements relevant to self-management experiences during adaptation were identified and extracted from the transcripts. Third, formulated meanings were developed from these significant statements while remaining grounded in participants’ original accounts. Fourth, related formulated meanings were organized into clusters and categories, from which preliminary themes were developed. Fifth, an exhaustive description of the phenomenon was constructed by integrating the thematic findings. Sixth, the fundamental structure of the phenomenon was identified by synthesizing the essential meanings across the themes. Finally, the resulting themes and fundamental structure were compared with the original transcripts to ensure that the findings remained consistent with participants’ accounts.*

*To enhance the depth and richness of the thematic findings, the researchers repeatedly reviewed the complete transcripts and examined significant statements in their original context rather than coding isolated excerpts. Open-ended questions and probing prompts were used during interviews to encourage participants to provide detailed descriptions of specific experiences, challenges, and coping responses. During analysis, emerging themes were repeatedly compared with the original transcripts to ensure that they captured the breadth and depth of participants’ accounts. Two researchers independently coded the transcripts and compared their interpretations, with discrepancies discussed within the research team until consensus was reached. Representative participant quotations were retained to illustrate and support the interpretation of each theme.*

*All interviews were conducted in Mandarin Chinese and transcribed verbatim in Chinese. For inclusion in the English-language manuscript, selected participant quotations were translated into English by the first author and reviewed by the second author against the original Chinese transcripts for accuracy and consistency of meaning. Any discrepancies were discussed and resolved by consensus. The analysis focused on participants’ lived experiences and perceived changes in self-management rather than on constructing a formal chronological or causal model of behavioral.*

**★ 20. Interview Language and Translation of Participant Quotations**

**Reviewer’s Comment:**

**Please clarify the language(s) used for the interviews and how the English translations of participant quotations were produced and verified.**

**➦Response**

Thank you for this important clarification. We have revised the **Data Analysis** section to explicitly describe the interview language and the process used to translate and verify participant quotations presented in the English-language manuscript.

*Specifically, we now state that all interviews were conducted in Mandarin Chinese and transcribed verbatim in Chinese. Selected participant quotations were translated into English by the first author and reviewed by the second author against the original Chinese transcripts to ensure accuracy and consistency of meaning. Any discrepancies in translation were discussed and resolved by consensus.*

This revision clarifies the linguistic procedures used in data collection and reporting and provides greater transparency regarding how the English-language participant quotations were produced and verified.

**★ 21. Assessment of Data Saturation**

**Reviewer’s Comment:**

**Who assessed saturation?**

**➦Response**

Thank you for this important clarification. We have revised the **Data Collection** section to explicitly identify who assessed data saturation and how the final decision was made. Specifically, the first author monitored saturation throughout data collection by reviewing interview transcripts and emerging codes for new information or codes. *After the 10th interview, no substantially new codes were identified, and three additional interviews were conducted to assess the stability and comprehensiveness of the emerging findings. After the 13th interview, no new codes or themes emerged. The first author then presented the saturation assessment to the research team, and the team discussed and confirmed that sufficient information had been obtained to address the research question. Data collection was subsequently terminated.*

This revision clarifies both the individual responsible for monitoring saturation and the process used by the research team to confirm the final decision to terminate data collection.

**★ 22. Thematic Depth**

**Reviewer’s Comment:**

**How was thematic depth ensured?**

**➦Response**

Thank you for this important methodological comment. We have revised the **Data Analysis** section to clarify how thematic depth was achieved and evaluated. *Specifically, the researchers repeatedly reviewed the complete transcripts and examined significant statements within their original context rather than coding isolated excerpts. During data collection, open-ended questions and probing prompts were used to encourage participants to provide detailed descriptions of their experiences, challenges, and coping responses. During analysis, emerging themes were repeatedly compared with the original transcripts to ensure that they adequately captured the breadth and depth of participants’ accounts. Two researchers independently coded the transcripts, and discrepancies in coding and interpretation were discussed within the research team until consensus was reached. Representative participant quotations were also retained to illustrate and support the interpretation of each theme.*

These revisions provide greater transparency regarding how the depth and richness of the themes were developed and evaluated.

**★ 23. Researcher Reflexivity and Potential Influence**

**Reviewer’s Comment:**

**How the first author’s clinical background, male gender, and assumptions regarding older adults, dependence, and stoma self-management may have influenced interviewing and interpretation.**

**➦Response**

Thank you for this important comment. We agree that the potential influence of the first author’s background and pre-existing assumptions should be described more explicitly. We have therefore expanded the **Rigour and Reflexivity** section to address these potential influences on both interviewing and interpretation.

Specifically, we now acknowledge that the first author’s clinical experience in gastrointestinal surgery facilitated understanding of stoma-related terminology and clinical contexts, but may also have directed attention toward clinically familiar aspects of self-management. We also acknowledge that the first author’s male gender might have influenced participants’ willingness to disclose sensitive experiences. In addition, we explicitly reflect on potential assumptions regarding older adults, dependence, and stoma self-management, including the possibility that greater age or dependence could be interpreted as poorer self-management.

To address these potential influences, the first author maintained reflexive notes documenting personal assumptions, reflections, and methodological decisions. Open-ended questions and probing prompts were used to allow participants to describe their experiences in their own terms rather than being directed toward predetermined interpretations. Regular research team discussions and independent coding were also used to challenge potential researcher assumptions and ensure that the themes remained grounded in participants’ narratives.

These revisions provide a more transparent account of researcher reflexivity and clarify how potential researcher influences were considered and addressed throughout data collection and analysis.

➦**Comparison before and after modification**

**Before modification:**2.7 Rigour and reflexivity

To enhance the methodological rigor of this qualitative study, the four criteria proposed by Lincoln and Guba, including credibility, dependability, confirmability, and transferability, were considered throughout the research process. Credibility was enhanced through purposive sampling to recruit participants with diverse experiences of living with a permanent enterostomy. Two researchers independently coded the interview transcripts, and coding results were compared and discussed within the research team until consensus was reached. To ensure dependability, detailed records of participant recruitment, data collection, and data analysis procedures were maintained to establish an audit trail. All transcripts were repeatedly reviewed and compared with the original audio recordings to ensure accuracy. To enhance confirmability, reflexive practices were incorporated throughout data analysis. The interviewer maintained reflexive notes documenting personal assumptions, reflections, and methodological decisions during data collection and interpretation. To minimize researcher bias during coding and interpretation, two researchers independently coded the interview transcripts, and coding results were compared and discussed within the research team until consensus was reached. Regular team discussions were conducted to identify potential biases and ensure that themes were grounded in participants’ narratives rather than researchers’ preconceptions. To enhance transferability, detailed descriptions of the research setting, participant characteristics, and sampling criteria were provided to allow readers to assess the applicability of the findings to other contexts. Data collection continued until data saturation was achieved. Data saturation was assessed throughout the data collection and analysis process. After the 10th interview, no substantially new codes were identified from the interview transcripts. However, to confirm the stability and comprehensiveness of the emerging findings, three additional interviews were conducted. After the 13th interview, no new codes or themes emerged, and the research team determined that sufficient information had been obtained to answer the research question. Therefore, participant recruitment and data collection were terminated.

Changed:**2.7 Rigour and reflexivity**

To enhance the methodological rigor of this qualitative study, the four criteria proposed by Lincoln and Guba, including credibility, dependability, confirmability, and transferability, were considered throughout the research process. Credibility was enhanced through purposive sampling to recruit participants with diverse experiences of living with a permanent stoma, together with independent coding and discussion within the research team. To ensure dependability, detailed records of participant recruitment, data collection, and data analysis procedures were maintained to establish an audit trail. Transcription accuracy was ensured by repeatedly reviewing transcripts against the original audio recordings.

*To enhance confirmability and reflexivity, the first author maintained reflexive notes documenting personal assumptions, reflections, and methodological decisions during data collection and interpretation. Particular attention was given to the potential influence of the first author’s clinical experience in gastrointestinal surgery, male gender, and pre-existing assumptions regarding older adults, dependence, and stoma self-management. His clinical experience facilitated understanding of stoma-related terminology and clinical contexts but could also have directed attention toward clinically familiar aspects of self-management.* The researcher also considered the possibility that gender might influence participants’ willingness to disclose sensitive experiences and that assumptions linking older age or greater dependence with poorer self-management could shape the interpretation of participants’ accounts. Open-ended questions and probing prompts were used to encourage participants to describe their experiences in their own terms rather than directing them toward predetermined interpretations. Regular team discussions were conducted to identify potential researcher bias, challenge alternative interpretations, and ensure that themes remained grounded in participants’ narratives rather than researchers’ preconceptions. To enhance transferability, detailed descriptions of the research setting, participant characteristics, and sampling criteria were provided to allow readers to assess the applicability of the findings to other contexts.

**★ 24. Reduction of Repetition Across Methods Subsections**

**Reviewer’s Comment:**

**Descriptions of data saturation, independent coding, and rigour are repeated across several subsections.**

**➦Response**

Thank you for this helpful comment. We agree that descriptions of data saturation, independent coding, and methodological rigor were repeated across several subsections in the previous version. We have therefore reorganized and streamlined the Methods section to avoid redundancy and to clarify the purpose of each subsection.

Specifically, detailed information on data saturation is now presented only in the Data Collection section, including who monitored saturation, how it was assessed, and how the final decision to terminate data collection was made. The procedures for independent coding and thematic analysis are described in the Data Analysis section in conjunction with the implementation of Colaizzi’s seven-step method. The Rigour and Reflexivity section now focuses on credibility, dependability, confirmability, transferability, and researcher reflexivity rather than repeating the detailed coding and saturation procedures.

We also removed duplicated descriptions of independent coding and saturation from the relevant subsections. These revisions improve the organization and conciseness of the Methods section while maintaining all essential methodological information.

**★ 25. Discussion: Too Long and Repetitive**

**Reviewer’s Comment:**

**The Discussion is too long and repetitive.**

**➦Response**

Thank you for this valuable comment. We agree that the Discussion in the previous version contained repetitive descriptions and could be presented more concisely. We have therefore substantially revised and streamlined the Discussion to improve its focus, clarity, and logical flow.

Specifically, we removed repeated descriptions of self-management as a dynamic or developmental process and consolidated overlapping interpretations across the three themes. In the first theme, we condensed the discussion of information conflicts, decision-making difficulties, and health literacy, and shortened the discussion of decision-aid tools and related clinical implications. In the second theme, we reduced repetitive descriptions of psychological adaptation and removed overly specific recommendations concerning phase-specific psychological interventions. In the third theme, we streamlined the discussion of resilience, removed the causal implication that resilience facilitates self-management, and clarified that resilience, psychological adaptation, self-efficacy, and self-management are related but conceptually distinct constructs.

We also reduced and softened clinical recommendations to ensure that they remained closely grounded in the findings of this qualitative study, while avoiding implications of intervention effectiveness that was not evaluated in the present study. In addition, the revised Discussion consistently presents participants’ retrospective perceptions rather than implying that changes in self-management were directly observed over time.

These revisions substantially reduce redundancy and improve the coherence and methodological consistency of the Discussion while preserving the key findings, interpretations, and clinically relevant implications.

**★ 26. Distinction Among Psychological Adaptation, Resilience, Self-Efficacy, and Self-Management**

**Reviewer’s Comment:**

**Psychological adaptation, resilience, self-efficacy, and self-management are sometimes presented as overlapping concepts or causal mechanisms.**

**➦Response**

Thank you for this important conceptual comment. We agree that the previous version did not sufficiently distinguish these related concepts and could imply overlapping or causal relationships among them. We have therefore revised the manuscript to clarify their conceptual boundaries and avoid unsupported causal interpretations.

Specifically, we now clarify that psychological adaptation, resilience, self-efficacy, and self-management are related but conceptually distinct constructs. Self-management remains the core phenomenon of the present study, while psychological adaptation concerns adjustment to illness-related changes, resilience concerns coping with and adapting to adversity, and self-efficacy concerns confidence in performing self-management behaviors. We have also removed or revised statements suggesting that psychological adaptation is a prerequisite for self-management or that resilience facilitates self-management. Instead, these concepts are described as related experiences that may coexist or become intertwined in participants’ retrospective accounts.

Given the descriptive phenomenological design and single retrospective interview, we further clarify that the present study does not establish causal relationships among these constructs. These revisions improve the conceptual clarity of the Discussion and ensure that the interpretation remains consistent with the study design and findings.

➦**Comparison before and after modification**

**Before modification:4 Discussion**

Our study employed in-depth interviews to explore the experiences of elderly patients living with a permanent stoma during long-term adaptation, with a particular focus on the development of self-management behaviors. The findings suggest that participants perceived self-management as an evolving experience shaped by ongoing challenges, adjustments, and accumulated experiences[8]. Three overarching themes were identified: “decision-making dilemmas and uncertainty,” “reconstructing daily life and identity after stoma formation,” and “developing resilience through accumulated experience and self-management practice.” These findings indicate that elderly patients’ self-management experiences are shaped by continuous interactions among stoma-related challenges, personal adaptation, and available support. Reflecting on their experiences after stoma formation, participants described a transition from initial uncertainty and disruption toward greater confidence in managing daily life and reconstructing personal meaning. Our findings provide a process-oriented understanding of how elderly patients gradually transform uncertainty and disruption into adaptive self-management through continuous learning, adjustment, and the use of available resources.

Because self-management of a permanent stoma permeates daily life and entails continuous behavioral adjustments, elderly patients are frequently required to make decisions across multiple domains, including dietary choices, stoma care, and physical activity[18].Previous research has indicated that[19]， Chinese patients with colorectal cancer commonly experience significant decision-making dilemmas when facing colostomy surgery; the findings of the present study align with these observations. This study revealed that when seeking health information online, patients often encounter cognitive conflicts arising from diverse and inconsistent information sources. This challenge is exacerbated when online content contradicts professional medical advice, thereby intensifying patients’ confusion and uncertainty in decision-making. Importantly, these decision-making difficulties were not merely emotional experiences; they directly affected patients’ capacity to initiate and maintain self-management behaviors[20], such as selecting appropriate foods, managing stoma-related complications, and deciding when to seek professional support.However, existing decision-aid tools for colorectal cancer tend to focus primarily on disease screening and the selection of treatment regimens, with insufficient attention to supporting long-term self-management decisions[21,22]. In addition to the complexities of the external information environment, difficulties in processing and applying health information may further complicate decision-making among some elderly patients, particularly when information is complex or inconsistent[23]. Constrained by their health literacy, some elderly patients may struggle to understand and retain care-related information, which can undermine their capacity for independent decision-making and their confidence in doing so[24]. Given these multifaceted factors—and against the backdrop of rapidly advancing digital health technologies—it is imperative to develop scientifically sound and standardized digital decision-aid tools[22]. By providing structured, simplified, and personalized information, such tools can effectively enhance patients’ decision-making capabilities in complex situations. Furthermore, this study observed that some patients exhibited a degree of reliance on family members during the decision-making process, highlighting the supportive role of family in self-management and healthcare decisions. This finding should be interpreted within the participants’ family context. In the present study, most participants were married and lived with family members, which may partly explain the prominent family involvement in daily stoma management and decision-making. In the context of Chinese family-oriented culture, family members often provide practical assistance and emotional support to elderly adults living with chronic conditions[25]. Therefore, with patients’ informed consent, appropriately involving family members in shared decision-making and health education processes may help improve decision quality and support long-term self-management[26]. In addition, healthcare professionals should provide individualized decision support by assessing patients’ information needs and health literacy levels, delivering structured and accessible education, and offering continuous guidance to facilitate daily self-management decisions[27].

Previous studies have confirmed that patients with stomas frequently face alterations in body image and psychological responses akin to a grief reaction[28]. Building on this, our findings further reveal that psychological adaptation is characterized by significant long-term, non-linear features[4,29]; even long after surgery, patients may continue to experience recurrent emotional fluctuations and identity conflicts. From a theoretical perspective, this process resonates with frameworks such as "meaning reconstruction," suggesting that patients do not simply return to a pre-illness state but, through continuous adjustment and adaptation, gradually construct a “new normal” that incorporates the presence of the stoma[30]. This psychological reconstruction may represent an important prerequisite for sustained self-management[31]. As patients gradually accepted the stoma and reconstructed their identity, they became more willing to engage in daily stoma care, seek solutions to complications, and integrate stoma management into their routines. Based on these findings, clinical practice should prioritize both the continuity of psychological support and the recognition of phase-specific patient needs[32]. Routine assessment of body image concerns, emotional distress, and adaptation difficulties during follow-up may help identify patients requiring additional psychological support[33]. During the early postoperative period, the focus should be on addressing patients’ body image distress and sense of loss of control, and on providing targeted psychological counseling. In the long-term follow-up phase, it is essential to identify patients who remain in a state of psychological conflict and, through interventions such as narrative therapy and peer support groups, facilitate meaning-making to enhance overall adaptation[34,35].

Our findings indicate that elderly patients living with a permanent stoma gradually developed psychological resilience through continuous self-adjustment and lived experience. The prominence of resilience in this study may be related to the cumulative life experiences of elderly adults[36]. Throughout their lives, elderly individuals may have encountered previous transitions, losses, and health-related challenges, which can serve as psychological resources for coping with the irreversible changes associated with permanent enterostomy[37]. Previous studies have often conceptualized resilience as an individual trait or a relatively stable personal resource that enables one to withstand adversity and maintain functioning[38]. However, our findings extend this perspective by suggesting that resilience among elderly patients with permanent stomas is not merely a pre-existing attribute but is gradually reconstructed through lived experience, continuous self-adjustment, and engagement in self-management practices. This interpretation aligns with contemporary resilience theories that emphasize resilience as a dynamic, developmental process shaped by person–environment interactions[39,40]. In this study, patients did not achieve effective self-management immediately after stoma formation. Instead, they gradually accumulated practical skills and confidence through repeated attempts, problem-solving, and adjustment in daily care routines. As patients became more familiar with stoma management, they shifted their attention from physical limitations toward maintaining daily activities, family relationships, and emotional well-being[41]. This process suggests that resilience functioned not only as a psychological coping resource but also as a facilitating factor supporting sustained self-management behaviors[42].While previous studies have largely focused on the psychological burden and negative experiences of stoma care[43], our findings highlight the adaptive capabilities of elderly patients and suggest that their accumulated life experiences may serve as valuable resources for reconstructing meaning, accepting bodily changes, and developing long-term self-management strategies[44]. These findings imply that interventions for elderly patients with permanent enterostomy should not focus solely on correcting deficits but should also incorporate a strength-based approach that recognizes patients’ existing abilities and life experiences. In clinical practice, healthcare professionals may encourage patients to reflect on successful self-management experiences, identify personal coping strategies, and participate in peer-support activities to enhance self-efficacy and promote sustained self-management behaviors[45,46].

Changed:**4 Discussion**

Our study employed in-depth interviews to explore the experiences of *older* patients living with a permanent stoma during adaptation, with a particular focus on participants’ perceived changes in self-management. The findings suggest that participants perceived self-management as an evolving experience shaped by ongoing challenges, adjustments, and accumulated experiences[9]. Three overarching themes were identified: “decision-making dilemmas and uncertainty,” “reconstructing daily life and identity after stoma formation,” and “developing resilience through accumulated experience and self-management practice.” Overall, participants described self-management as an evolving lived experience involving practical care, decision-making, adjustment to changes in daily life, and use of available support. Psychological adaptation, resilience, and self-efficacy appeared in participants’ accounts as related experiences or resources, but they represent conceptually distinct aspects of living with and managing a permanent stoma. *Given the single retrospective interview, these concepts should therefore be understood as related but distinct aspects of participants’ experiences rather than as sequential stages or causal mechanisms.*

Because self-management of a permanent stoma permeates daily life and entails continuous behavioral adjustments, older patients are frequently required to make decisions across multiple domains, including dietary choices, stoma care, and physical activity[19].Previous research has indicated that[20]， Chinese patients with colorectal cancer commonly experience significant decision-making dilemmas when facing colostomy surgery; the findings of the present study align with these observations. Participants described encountering conflicting health information online, particularly when online advice differed from professional recommendations, which increased uncertainty about appropriate self-management strategies[21], such as selecting appropriate foods, managing stoma-related complications, and deciding when to seek professional support.However, existing decision-aid tools for colorectal cancer tend to focus primarily on disease screening and the selection of treatment regimens, with insufficient attention to supporting long-term self-management decisions[22,23]. *In addition to the complexities of the external information environment, difficulties in processing and applying health information may further complicate decision-making among some older patients, particularly when information is complex or inconsistent[24]. Constrained by their health literacy, some older patients may struggle to understand and retain care-related information, which can undermine their capacity for independent decision-making and their confidence in doing so[25]. Participants also described relying on family members for some decisions and aspects of daily stoma management. This finding should be interpreted in the context of participants’ family circumstances, as most were married and lived with family members. In Chinese families, family members may provide practical and emotional support to older adults living with chronic conditions[26]. Rather than indicating inadequate self-management, such reliance may represent one way in which self-management is supported within the family context. These findings highlight the potential value of individualized and accessible decision support for older adults with permanent stomas. Structured information, clear guidance on evaluating conflicting health information, and opportunities for ongoing clarification may help address patients’ reported uncertainty[27]. When consistent with patients’ preferences, involving family members in health education and shared decision-making may also provide practical and emotional support[28]. Digital decision-aid tools may be a potential approach to providing such support, but their effectiveness for long-term stoma self-management requires further evaluation[23].*

Previous studies have confirmed that living with a stoma can affect body image, daily routines, and emotional well-being[29]. *Consistent with these findings, participants described psychological adaptation as an ongoing process involving changes in body image, identity, daily life, and emotional responses. Some participants described periods of rejection, distress, or uncertainty, whereas others described gradually incorporating the stoma into their daily lives and developing a sense of a “new normal”[30,31]; These accounts suggest that psychological adaptation and self-management are closely related but distinct dimensions of the adaptation experience[32]. Participants’ descriptions indicated that changes in emotional and identity-related experiences often occurred alongside changes in their approach to stoma care and daily activities; however, the present study cannot determine whether psychological adaptation led to changes in self-management or vice versa. From a theoretical perspective, this pattern is consistent with meaning-reconstruction perspectives, which emphasize how individuals make sense of illness-related changes and incorporate them into their ongoing lives[33,34]. These findings highlight the potential value of follow-up care that addresses both practical self-management needs and psychological concerns. Assessment of body image concerns, emotional distress, and difficulties incorporating the stoma into daily life may help clinicians identify patients who perceive greater challenges during adaptation[35,36].Psychological and supportive care can be tailored to patients’ expressed concerns and preferences rather than assuming a uniform adaptation trajectory.*

Previous studies have often conceptualized resilience as an individual trait or a relatively stable personal resource that enables one to withstand adversity and maintain functioning[37]. However, our findings extend this perspective by suggesting that resilience among older patients with permanent stomas is not merely a pre-existing attribute but is gradually reconstructed through lived experience, continuous self-adjustment, and engagement in self-management practices. This interpretation aligns with contemporary resilience theories that emphasize resilience as a dynamic, developmental process shaped by person-environment interactions[38,39]. Participants frequently described accumulating practical experience, learning from unsuccessful attempts, and developing greater familiarity with stoma care. These accounts were often accompanied by descriptions of increased confidence and broader attention to maintaining daily activities, family relationships, and emotional well-being[40]. *Rather than indicating a causal pathway from resilience to self-management, these findings suggest that resilience-related coping experiences and self-management practices may become intertwined in participants’ accounts over the course of adaptation. This interpretation is consistent with a strength-based perspective that recognizes older adults’ accumulated life experiences as potential resources for coping with bodily changes, reconstructing meaning, and managing everyday challenges[41,42]. In clinical practice, a strength-based approach may help patients recognize existing abilities and successful experiences in stoma management. Encouraging patients to reflect on situations they have successfully managed, identify personally meaningful coping strategies, and exchange experiences with peers may help reinforce confidence in their ability to manage everyday challenges[43,44].* ***Such approaches may be relevant to self-efficacy, but self-efficacy should be considered a related construct rather than a direct component or established causal mechanism of self-management in the present study.***

**★ 27. Clinical Recommendations and Evidence Support**

**Reviewer’s Comment:**

**Some clinical recommendations are not sufficiently supported by the findings.**

**➦Response**

Thank you for this important comment. We agree that some of the clinical recommendations in the previous version extended beyond what could be directly supported by the findings of this qualitative study. We have therefore revised and streamlined the clinical implications throughout the **Discussion** to ensure that they remain closely grounded in participants’ accounts.

Specifically, we removed overly specific recommendations, including the previous suggestions regarding phase-specific psychological interventions, narrative therapy, and peer-support interventions. We also revised statements concerning digital decision-aid tools and strength-based approaches to avoid implying established effectiveness. The remaining clinical implications are now presented more cautiously using language such as “may,” “potential value,” and “could be considered,” and are directly linked to the issues identified by participants, including decision-making uncertainty, psychological concerns, family support, and accumulated personal resources.

We further clarified that the effectiveness of these proposed approaches was not evaluated in the present study and therefore cannot be inferred from our qualitative findings. These revisions ensure that the clinical implications are appropriately proportionate to the evidence generated by the study.

**★ 28. Conclusion: Avoiding Implications of Directly Observed Dynamic Change**

**Reviewer’s Comment:**

**The Conclusion should not imply that a dynamic process was directly observed.**

**➦Response**

Thank you for this important clarification. We agree that the previous Conclusion could imply that changes in self-management were directly observed over time, which is not supported by the single retrospective interview design. We have therefore revised the Conclusion to distinguish participants’ retrospective accounts and perceived changes in self-management from direct observation of longitudinal behavioral development.

Specifically, we replaced statements describing self-management as a directly observed “dynamic process” with wording emphasizing participants’ perceived changes in relation to decisional uncertainty, psychological adjustment, accumulated experience, and resilience-related coping. We also added an explicit statement that the findings reflect participants’ perceptions of change rather than direct observation of self-management development over time.

These revisions ensure that the Conclusion is consistent with the descriptive phenomenological design and the single retrospective interview and does not overstate the longitudinal or causal implications of the findings.

➦**Comparison before and after modification**

**Before modification:5 Conclusion**

This qualitative study explored elderly patients’ experiences of self-management behaviors during adaptation to permanent enterostomy. The findings suggest that patients experienced self-management as a dynamic process shaped by decisional uncertainty, non-linear psychological adjustment, and the development of resilience. Accordingly, healthcare professionals should pay greater attention to long-term self-management support in this population by strengthening individualized decision support, providing continuous psychological care, appropriately involving family members according to patients’ preferences, and adopting a strength-based care approach.

Changed:**5 Conclusion**

This qualitative study explored the lived experiences and meanings of self-management among older adults with permanent stomas during adaptation. *Participants’ retrospective accounts suggested perceived changes in self-management in relation to decisional uncertainty, psychological adjustment, accumulated experience, and resilience-related coping. These findings highlight the importance of individualized decision support, attention to psychological concerns, family involvement consistent with patients’ preferences, and strength-based care. Given the single retrospective interview, the findings reflect participants’ perceptions of change rather than direct observation of self-management development over time.*

**★ 29. Sample Size as a Limitation**

**Reviewer’s Comment:**

**A small sample size is not inherently a limitation in phenomenological research.**

**➦Response**

Thank you for this important methodological clarification. We agree that a relatively small sample is not, in itself, a limitation of phenomenological research, where sample adequacy is guided by the richness and sufficiency of the information obtained rather than statistical representativeness.

Accordingly, we have removed the reference to the small sample size as a limitation from the Limitations section. We have instead focused the limitations on issues more directly related to the study design and recruitment process, including single-centre recruitment, potential self-selection through the specialist stoma clinic, variation in time since stoma formation, recall bias, and the use of a single retrospective interview. These changes provide a more methodologically appropriate account of the limitations of the present study.

➦**Comparison before and after modification**

**Before modification:6 Limitations**

This study has several limitations. First, the relatively small sample size and recruitment from a single tertiary hospital may limit the transferability of the findings to other settings with different cultural and healthcare contexts. Second, variations in participant characteristics, such as gender distribution and postoperative duration, may have influenced the results, and some experiences may be underrepresented. Third, as interviews were conducted in Chinese and later translated into English, subtle nuances in meaning may have been affected despite careful translation procedures; additionally, recall bias may be present as participants reflected on past experiences. Finally, although participants were encouraged to retrospectively describe changes in their self-management behaviors from stoma formation to the present, data were collected through a single interview at one time point. Therefore, the findings represent participants’ retrospective accounts of their adaptation experiences rather than direct observation of changes across different stages of adaptation. Future longitudinal qualitative studies with repeated interviews at multiple time points are needed to further explore the dynamic changes in self-management behaviors and adaptation experiences among elderly patients with permanent enterostomy.

Changed:**6 Limitations**

This study has several limitations. First, *participants were recruited from a single tertiary hospital, which may limit the transferability of the findings to other healthcare settings and cultural contexts. In addition, recruitment through a specialist stoma clinic may have introduced self-selection, as individuals who maintained contact with specialized stoma services or were more willing to engage in stoma-related care may have been more likely to participate. Second, participants varied in the duration since stoma formation. Although this variation was intentionally retained to capture diverse retrospective experiences, it may have contributed to differences in participants’ perceptions of adaptation and self-management. Third, because participants were asked to retrospectively describe experiences from stoma formation to the time of interview, recall bias cannot be excluded. Interviews were conducted in Mandarin Chinese and selected quotations were translated into English; although the translations were checked against the original transcripts, some linguistic or contextual nuances may not have been fully preserved. Finally, data were collected through a single retrospective interview at one time point. Therefore, the study captured participants’ perceived changes in self-management rather than directly observing changes across different stages of adaptation. Longitudinal qualitative studies using repeated interviews at multiple time points could provide richer insights into how self-management experiences develop and change over time.*

**★ 30. Important Study Limitations**

**Reviewer’s Comment:**

**Important limitations include single-centre recruitment, self-selection through a specialist stoma clinic, wide variation in time since stoma formation, recall bias, and the use of a single retrospective interview.**

**➦Response**

Thank you for this important and helpful suggestion. We have revised the Limitations section to more explicitly acknowledge the study limitations related to the recruitment setting, participant characteristics, and data collection design.

Specifically, we now acknowledge that participants were recruited from a single tertiary hospital, which may limit the transferability of the findings to other healthcare settings and cultural contexts. We also clarify that recruitment through the specialist stoma clinic may have introduced self-selection, as individuals who maintained contact with specialized stoma services or were more willing to engage in stoma-related care may have been more likely to participate. In addition, we explicitly acknowledge the variation in time since stoma formation and explain that this variation was intentionally retained to capture diverse retrospective experiences rather than to compare predefined postoperative stages. We further acknowledge the potential for recall bias, as participants were asked to retrospectively describe experiences from stoma formation to the time of interview.

Finally, we now explicitly identify the single retrospective interview at one time point as a limitation. We clarify that the study captures participants’ perceived changes in self-management rather than directly observing changes across different stages of adaptation, and we note that longitudinal qualitative studies using repeated interviews at multiple time points could provide richer insights into how self-management experiences develop and change over time.

These revisions provide a more comprehensive and methodologically appropriate description of the limitations of the present study.

- **Section4. Response to Reviewer #4:**

We sincerely appreciate Reviewer for the careful evaluation of our revised manuscript and for the thoughtful and constructive methodological comment. We are grateful for the reviewer’s important clarification regarding the distinction between the limitations of the cross-sectional, single-time-point study design and those of qualitative research more broadly. This comment helped us further refine the description of the study limitations and ensure that our interpretation of the findings is appropriately aligned with the study design.

**★ 1. Clarification of the Limitation Related to the Single-Interview Design**

**Reviewer’s Comment:**

The authors appropriately acknowledge that the single-interview design limits their ability to capture the evolution of self-management behaviors over time. The limitation therefore relates to the cross-sectional, single-time-point data collection, rather than to qualitative methodology itself. A qualitative longitudinal design with repeated interviews at different stages of adaptation could have provided richer insights into how self-management behaviors develop and change. The authors are encouraged to revise this statement to accurately distinguish the limitations of the chosen study design from those of qualitative research more broadly.

**➦Response**

Thank you very much for this important clarification. We agree that the limitation is attributable to the cross-sectional, single-time-point data collection and the use of a single retrospective interview, rather than to qualitative methodology itself.

Accordingly, we have revised the Limitations section to explicitly state that the study captured participants’ retrospective accounts and perceived changes in self-management, rather than directly observing changes across different stages of adaptation. We have also removed wording that could imply that the qualitative design itself was a limitation.

In addition, we now acknowledge that longitudinal qualitative studies involving repeated interviews at multiple time points could provide richer insights into how self-management experiences develop and change over time. This revision more accurately reflects the methodological boundary of the present study and avoids overinterpreting participants’ retrospective accounts as directly observed longitudinal changes.
