# Supplementary material for: Experiences of self-management behaviors during adaptation to permanent enterostomy among older patients: A qualitative study
Source: PLoS One. 2026 Oct 29;21(10):e0360643. doi: 10.1371/journal.pone.0360643 (PMC13718964; doi:10.1371/journal.pone.0360643)
Supplement: Peer review history

# Peer review: Decision Letter 0

**DOI:** 10.1371/journal.pone.0360643.r001

**Authors:** Jamshed Akhtar

22 Jul 2026

Dear Dr. Wu,

Thank you for submitting your manuscript to PLOS ONE. After careful consideration, we feel that it has merit but does not fully meet PLOS ONE’s publication criteria as it currently stands. Therefore, we invite you to submit a revised version of the manuscript that addresses the points raised during the review process.

Please submit your revised manuscript by Sep 20 2026 11:59PM. If you will need more time than this to complete your revisions, please reply to this message or contact the journal office at plosone@plos.org. When you're ready to submit your revision, log on to https://www.editorialmanager.com/pone/ and select the 'Submissions Needing Revision' folder to locate your manuscript file.

- A letter that responds to each point raised by the academic editor and reviewer(s). You should upload this letter as a separate file labeled 'Response to Reviewers'.
- A marked-up copy of your manuscript that highlights changes made to the original version. You should upload this as a separate file labeled 'Revised Manuscript with Track Changes'.
- An unmarked version of your revised paper without tracked changes. You should upload this as a separate file labeled 'Manuscript'.

If you would like to make changes to your financial disclosure, please include your updated statement in your cover letter. Guidelines for resubmitting your figure files are available below the reviewer comments at the end of this letter.

If applicable, we recommend that you deposit your laboratory protocols in protocols.io to enhance the reproducibility of your results. Protocols.io assigns your protocol its own identifier (DOI) so that it can be cited independently in the future. For instructions see: https://journals.plos.org/plosone/s/submission-guidelines#loc-laboratory-protocols. Additionally, PLOS ONE offers an option for publishing peer-reviewed Lab Protocol articles, which describe protocols hosted on protocols.io. Read more information on sharing protocols at https://plos.org/protocols?utm_medium=editorial-email&utm_source=authorletters&utm_campaign=protocols.

As the corresponding author, your ORCID iD is verified in the submission system and will appear in the published article. PLOS supports the use of ORCID, and we encourage all coauthors to register for an ORCID iD and use it as well. Please encourage your coauthors to verify their ORCID iD within the submission system before final acceptance, as unverified ORCID iDs will not appear in the published article. *Only*  the individual author can complete the verification step; PLOS staff *cannot*  verify ORCID iDs on behalf of authors.

We look forward to receiving your revised manuscript.

Kind regards,

Jamshed Akhtar, MBBS, FCSP, FRCS, MHPE, FACS, M Bioethics

Academic Editor

PLOS One

Journal Requirements:

When submitting your revision, we need you to address these additional requirements.

1. Please ensure that your manuscript meets PLOS ONE's style requirements, including those for file naming. The PLOS ONE style templates can be found at

https://journals.plos.org/plosone/s/file?id=wjVg/PLOSOne_formatting_sample_main_body.pdf and

https://journals.plos.org/plosone/s/file?id=ba62/PLOSOne_formatting_sample_title_authors_affiliations.pdf.

2. When completing the data availability statement of the submission form, you indicated that you will make your data available on acceptance. We strongly recommend all authors decide on a data sharing plan before acceptance, as the process can be lengthy and hold up publication timelines. Please note that, though access restrictions are acceptable now, your entire data will need to be made freely accessible if your manuscript is accepted for publication. This policy applies to all data except where public deposition would breach compliance with the protocol approved by your research ethics board. If you are unable to adhere to our open data policy, please kindly revise your statement to explain your reasoning and we will seek the editor's input on an exemption. Please be assured that, once you have provided your new statement, the assessment of your exemption will not hold up the peer review process.

3. Please include a copy of Table xxxx which you refer to in your text on page 9.

4. We notice that your supplementary table are included in the manuscript file. Please remove them and upload them with the file type 'Supporting Information'. Please ensure that each Supporting Information file has a legend listed in the manuscript after the references list.

5. We note that there is identifying data in the Supporting Information file (S1 Table 1). Prior to sharing human research participant data, authors should consult with an ethics committee to ensure data are shared in accordance with participant consent and all applicable local laws.

Data sharing should never compromise participant privacy. It is therefore not appropriate to publicly share personally identifiable data on human research participants. The following are examples of data that should not be shared:

-Name, initials, physical address

-Ages more specific than whole numbers

-Internet protocol (IP) address

-Specific dates (birth dates, death dates, examination dates, etc.)

-Contact information such as phone number or email address

-Location data

-ID numbers that seem specific (long numbers, include initials, titled “Hospital ID”) rather than random (small numbers in numerical order)

Data that are not directly identifying may also be inappropriate to share, as in combination they can become identifying. For example, data collected from a small group of participants, vulnerable populations, or private groups should not be shared if they involve indirect identifiers (such as sex, ethnicity, location, etc.) that may risk the identification of study participants.

Additional guidance on preparing raw data for publication can be found in our Data Policy (https://journals.plos.org/plosone/s/data-availability#loc-human-research-participant-data-and-other-sensitive-data) and in the following article: http://www.bmj.com/content/340/bmj.c181.long.

Please remove or anonymize all personal information (<specific identifying information in file to be removed>), ensure that the data shared are in accordance with participant consent, and re-upload a fully anonymized data set. Please note that spreadsheet columns with personal information must be removed and not hidden as all hidden columns will appear in the published file.

6. If the reviewer comments include a recommendation to cite specific previously published works, please review and evaluate these publications to determine whether they are relevant and should be cited. There is no requirement to cite these works unless the editor has indicated otherwise.

Additional Editor Comments (if provided):

The subject is important and representing a particular geographic region is also appreciated. However, the depth of the subject as mentioned in the title, like adaptation and long term are not adequately addressed in this qualitative study with sample size based upon different sub set of patients, not appropriately reflect that. How data saturation is arrived with diverse experiences is not clear.

[Note: HTML markup is below. Please do not edit.]

Reviewers' comments:

Reviewer's Responses to Questions

**Comments to the Author**

1. Is the manuscript technically sound, and do the data support the conclusions?

Reviewer #1: Partly

Reviewer #2: Partly

Reviewer #3: Yes

Reviewer #4: Yes

**********

2. Has the statistical analysis been performed appropriately and rigorously?

Reviewer #1: No

Reviewer #2: N/A

Reviewer #3: N/A

Reviewer #4: N/A

**********

3. Have the authors made all data underlying the findings in their manuscript fully available?

The PLOS Data policy

Reviewer #1: No

Reviewer #2: Yes

Reviewer #3: Yes

Reviewer #4: Yes

**********

4. Is the manuscript presented in an intelligible fashion and written in standard English?

Reviewer #1: No

Reviewer #2: Yes

Reviewer #3: Yes

Reviewer #4: Yes

**********

Reviewer #1: Manuscript Title: Experiences of Self-Management Behaviors in the Long-Term Adaptation Process of Elderly Patients with Permanent Enterostomy: A Qualitative Study

Overall, this manuscript addresses an important and clinically relevant topic. The focus on elderly patients with permanent enterostomies is valuable because this population has unique psychological, cognitive, and self-management challenges that are often underrepresented in the literature. However, several important methodological and reporting issues should be addressed.

1-The manuscript should explicitly state that it follows the Consolidated Criteria for Reporting Qualitative Research (COREQ) checklist. A completed COREQ checklist should be submitted as supplementary material

2-The authors state that a phenomenological approach was used,please clarify Why was this methodology chosen over grounded theory or thematic analysis?

3-Please report: Interviewer's previous experience in qualitative research.,Relationship between interviewer and participants.,Whether interviewer assumptions were documented through reflexive journaling., How researcher bias was minimized throughout coding and interpretation.

4-The demographic table is limited.

5-Additional variables would improve transferability: Primary diagnosis,Time since cancer diagnosis, Type of surgery

Presence of complications,Educational level categorized more clearly and Living alone versus with family

6-The interview guide contains only broad questions.Please indicate: Which theoretical framework informed the interview questions. And Whether pilot interviews resulted in modifications.

7-Participant numbering inconsistency

8-The Discussion largely summarizes previous literature. The authors should better emphasize: What is genuinely novel about elderly patients. How their findings differ from previous qualitative studies. And Why resilience emerged as a dominant theme

9-The manuscript is generally readable but requires professional English editing.requires Standardize terminology

10-Remove unnecessary repetition in the Introduction.

11-Verify that all references follow PLOS ONE formatting.

12-The paper lacks a clear, operationalized definition of "self-management" despite this being the central concept.

13-There is conceptual overlap between themes

14-The paper identifies important challenges but does not address practical implementation

15-Wide range of 20-40 minutes with single interviews. Is 20 minutes sufficient for in-depth exploration? Single interview may not capture the evolution of experiences

Reviewer #2: Thank you for the opportunity to review this manuscript. The study addresses an important and clinically relevant topic concerning self-management among older adults living with permanent stomas. However, the manuscript would benefit from substantial revision to improve conceptual clarity, methodological transparency, and alignment between the stated aim, study design, findings, and discussion. In particular, the authors should clearly define self-management and distinguish it from related concepts such as self-care, psychological adaptation, independence, and self-efficacy. Greater detail is also needed regarding the study setting, participant characteristics, time since stoma formation, sampling rationale, interview guide development, reflexivity, and trustworthiness procedures. The findings currently appear to emphasise psychological adaptation more than the formation and evolution of practical self-management behaviours, and the discussion and conclusion should be moderated accordingly. The manuscript should also standardise terminology, avoid potentially ageist or overgeneralised statements, and present claims about cognitive decline, behavioural change over time, and proposed interventions more cautiously and with stronger support from the data or literature.

Reviewer #3: Dear Authors,

I would like to express my gratitude for the opportunity to review your manuscript. After completing the review, I have compiled some feedback and suggestions that I hope will help enhance the quality of the manuscript. Kindly review and revise the manuscript according to the feedback provided. Please highlight with a different color the part you revised. Thank you for your attention and cooperation.

Best regards,

Reviewer

Reviewer #4: Strengths:

• This qualitative study tackles an important and under-researched topic, filling a notable gap in the literature. Its uniqueness lies in two key aspects:

1. It employs a qualitative phenomenological approach that deeply examines behaviors.

2. It focuses on the developmental process of self-management rather than merely describing problems, offering valuable practical implications for patients’ long-term care.

• The rationale for the study is clear and well-articulated.

• The chosen methodology is appropriate for a qualitative inquiry.

Weaknesses:

• There is a noticeable disconnect between the interview questions and the study’s stated aim. While the research seeks to explore the developmental process of self-management, the questions do not sufficiently probe the evolution of behaviors across different phases of adaptation (e.g., “How did you gradually learn to manage this?” or “What difficulties did you encounter along the way?”). Because the interviews were conducted as a single, one-time event, they captured a relatively fluid narrative rather than distinct phases. A longitudinal design with multiple time points interviews would have better addressed this aim. (Authors mentioned this in limitations of being unable to conduct the longitudinal design due to qualitative nature of the study?)

• The study includes patients at markedly different stages of long-term adaptation (e.g., 4 months versus several years post-surgery), yet it does not adequately justify the comparability of these experiences.

• With only 13 participants, the claim of data saturation is difficult to justify. The researchers should provide a more detailed account of how saturation was determined and why additional participants were (or were not) added.

• The socioeconomic and cultural backgrounds of the participants are not described. Given the variability in family structures and their strong influence on attitudes toward illness and aging—particularly within the Inner Mongolian healthcare and cultural context—this information is important for contextualizing the findings.

• Labeling the identified themes as “novel” appears overstated, as similar themes are extensively discussed in existing literature, including few of the studies cited by the authors.

• The researcher's/interviewer’s positionality (including professional background) is not addressed. For example, the dynamics of a professor or nurse interviewing elderly participants merit critical reflection, as these factors can influence the data collected.

Despite these limitations, the work provided meaningful insights for clinicians involved in stoma care of elderly patients.

**********

what does this mean?). If published, this will include your full peer review and any attached files.

If you choose “no”, your identity will remain anonymous but your review may still be made public.

**Do you want your identity to be public for this peer review?**  For information about this choice, including consent withdrawal, please see our Privacy Policy

Reviewer #1: **Yes:**  Tamer.A.A.M.Habeeb

Reviewer #2: No

Reviewer #3: **Yes:**  Ratna Aryani

Reviewer #4: No

**********

[NOTE: If reviewer comments were submitted as an attachment file, they will be attached to this email and accessible via the submission site. Please log into your account, locate the manuscript record, and check for the action link "View Attachments". If this link does not appear, there are no attachment files.]

To ensure your figures meet our technical requirements, please review our figure guidelines: https://journals.plos.org/plosone/s/figures

You may also use PLOS’s free figure tool, NAAS, to help you prepare publication quality figures: https://journals.plos.org/plosone/s/figures#loc-tools-for-figure-preparation.

NAAS will assess whether your figures meet our technical requirements by comparing each figure against our figure specifications.

# Author comment: Author response to Decision Letter 1

**DOI:** 10.1371/journal.pone.0360643.r002

31 Jul 2026

We deeply appreciate the thoughtful comments and encouragement regarding our manuscript. Receiving feedback from such experienced scholars is a tremendous help in strengthening our study on elderly patients with permanent enterostomy. We have undertaken extensive revisions to address the concerns raised, particularly focusing on enhancing the transparency of our research methods, refining the discussion of our findings, and ensuring full compliance with the journal’s data-sharing policies. It has been a rewarding learning process to align our work with the high standards of PLOS ONE. If any aspects of our revision remain imperfect or fail to meet the journal's expectations, we apologize for any oversight and welcome further guidance. We are committed to making any additional corrections promptly.

Thank you again for your patience and dedication.

Attachment Submitted filename: Response to Reviewers.docx

# Peer review: Decision Letter 1

**DOI:** 10.1371/journal.pone.0360643.r003

**Authors:** Jamshed Akhtar

10 Sep 2026

Dear Dr. Wu,

Thank you for submitting your manuscript to PLOS One. After careful consideration, we feel that it has merit but does not fully meet PLOS One’s publication criteria as it currently stands. Therefore, we invite you to submit a revised version of the manuscript that addresses the points raised during the review process.

Please submit your revised manuscript by . If you will need more time than this to complete your revisions, please reply to this message or contact the journal office at plosone@plos.org. When you're ready to submit your revision, log on to https://www.editorialmanager.com/pone/ and select the 'Submissions Needing Revision' folder to locate your manuscript file.

- A letter that responds to each point raised by the academic editor and reviewer(s). You should upload this letter as a separate file labeled 'Response to Reviewers'.
- A marked-up copy of your manuscript that highlights changes made to the original version. You should upload this as a separate file labeled 'Revised Manuscript with Track Changes'.
- An unmarked version of your revised paper without tracked changes. You should upload this as a separate file labeled 'Manuscript'.

If you would like to make changes to your financial disclosure, please include your updated statement in your cover letter. Guidelines for resubmitting your figure files are available below the reviewer comments at the end of this letter.

If applicable, we recommend that you deposit your laboratory protocols in protocols.io to enhance the reproducibility of your results. Protocols.io assigns your protocol its own identifier (DOI) so that it can be cited independently in the future. For instructions see: https://journals.plos.org/plosone/s/submission-guidelines#loc-laboratory-protocols. Additionally, PLOS One offers an option for publishing peer-reviewed Lab Protocol articles, which describe protocols hosted on protocols.io. Read more information on sharing protocols at https://plos.org/protocols?utm_medium=editorial-email&utm_source=authorletters&utm_campaign=protocols.

As the corresponding author, your ORCID iD is verified in the submission system and will appear in the published article. PLOS supports the use of ORCID, and we encourage all coauthors to register for an ORCID iD and use it as well. Please encourage your coauthors to verify their ORCID iD within the submission system before final acceptance, as unverified ORCID iDs will not appear in the published article. *Only*  the individual author can complete the verification step; PLOS staff *cannot*  verify ORCID iDs on behalf of authors.

We look forward to receiving your revised manuscript.

Kind regards,

Jen Edwards

Staff Editor

PLOS One

Jamshed Akhtar, MBBS, FCSP, FRCS, MHPE, FACS, M Bioethics

Academic Editor

PLOS One

Journal Requirements:

If the reviewer comments include a recommendation to cite specific previously published works, please review and evaluate these publications to determine whether they are relevant and should be cited. There is no requirement to cite these works unless the editor has indicated otherwise.

Additional Editor Comments :

There are observations of the reviewer that authors are required to respond to.

[Note: HTML markup is below. Please do not edit.]

Reviewers' comments:

Reviewer's Responses to Questions

**Comments to the Author**

Reviewer #1: All comments have been addressed

Reviewer #3: (No Response)

Reviewer #4: All comments have been addressed

**********

2. Is the manuscript technically sound, and do the data support the conclusions?

Reviewer #1: Yes

Reviewer #3: Yes

Reviewer #4: Yes

**********

3. Has the statistical analysis been performed appropriately and rigorously?

Reviewer #1: Yes

Reviewer #3: N/A

Reviewer #4: N/A

**********

4. Have the authors made all data underlying the findings in their manuscript fully available?

The PLOS Data policy

Reviewer #1: Yes

Reviewer #3: No

Reviewer #4: Yes

**********

5. Is the manuscript presented in an intelligible fashion and written in standard English?

Reviewer #1: Yes

Reviewer #3: No

Reviewer #4: Yes

**********

Reviewer #1: Thanks to the authors for the revised manuscript .The revised manuscript is now more better and suitable for consideration for publication

Reviewer #3: (No Response)

Reviewer #4: The authors appropriately acknowledge that the single-interview design limits their ability to capture the evolution of self-management behaviors over time.

The limitation therefore relates to the cross-sectional, single-time-point data collection, rather than to qualitative methodology itself. A qualitative longitudinal design with repeated interviews at different stages of adaptation could have provided richer insights into how self-management behaviors develop and change. The authors are encouraged to revise this statement to accurately distinguish the limitations of the chosen study design from those of qualitative research more broadly.

**********

what does this mean?). If published, this will include your full peer review and any attached files.

If you choose “no”, your identity will remain anonymous but your review may still be made public.

**Do you want your identity to be public for this peer review?**  For information about this choice, including consent withdrawal, please see our Privacy Policy

Reviewer #1: **Yes:**  Tamer.A.A.M.Habeeb

Reviewer #3: No

Reviewer #4: No

**********

[NOTE: If reviewer comments were submitted as an attachment file, they will be attached to this email and accessible via the submission site. Please log into your account, locate the manuscript record, and check for the action link "View Attachments". If this link does not appear, there are no attachment files.]

To ensure your figures meet our technical requirements, please review our figure guidelines: https://journals.plos.org/plosone/s/figures

You may also use PLOS’s free figure tool, NAAS, to help you prepare publication quality figures: https://journals.plos.org/plosone/s/figures#loc-tools-for-figure-preparation.

NAAS will assess whether your figures meet our technical requirements by comparing each figure against our figure specifications.

Attachment Submitted filename: 2. Review PLOSONE.pdf

# Author comment: Author response to Decision Letter 2

**DOI:** 10.1371/journal.pone.0360643.r004

15 Sep 2026

We sincerely thank the Academic Editor and all reviewers for their careful evaluation and constructive comments on our manuscript (PONE-D-26-23135R1). We have carefully considered all comments and suggestions and have revised the manuscript accordingly. A detailed point-by-point response is provided in the accompanying “Response to Reviewers” document, and all corresponding changes are highlighted in the revised manuscript with Track Changes. A clean version of the revised manuscript has also been submitted.

In this revision, we substantially streamlined and refocused the Introduction and Discussion, clarified the conceptual distinctions among self-management, self-care, psychological adaptation, independence, resilience, and self-efficacy, and more clearly defined the specific research gap and novel contribution of the study. We also clarified the descriptive phenomenological design, the implementation of Colaizzi’s seven-step method, data saturation assessment, thematic depth, purposive sampling, participant recruitment, translation procedures, and researcher reflexivity. In addition, we revised the interpretation and clinical implications to avoid unsupported causal claims and strengthened the Limitations and Conclusion to accurately reflect the single retrospective interview and cross-sectional, single-time-point design.

We have also addressed the data availability and language-related concerns raised in the review process. We sincerely appreciate the time and expertise of the Academic Editor and reviewers, whose comments have substantially strengthened the clarity, methodological rigor, and overall quality of our manuscript. We hope that the revised manuscript adequately addresses all concerns and is now suitable for further consideration in PLOS ONE.

Attachment Submitted filename: Response_to_Reviewers_auresp_2.docx

# Peer review: Decision Letter 2

**DOI:** 10.1371/journal.pone.0360643.r005

**Authors:** Jamshed Akhtar

29 Sep 2026

Experiences of Self-Management Behaviors During Adaptation to Permanent Enterostomy Among Older Patients: A Qualitative Study

PONE-D-26-23135R2

Dear Dr. Wu,

We’re pleased to inform you that your manuscript has been judged scientifically suitable for publication and will be formally accepted for publication once it meets all outstanding technical requirements.

Within one week, you’ll receive an e-mail detailing the required amendments. When these have been addressed, you’ll receive a formal acceptance letter and your manuscript will be scheduled for publication.

An invoice will be generated when your article is formally accepted. Please note, if your institution has a publishing partnership with PLOS and your article meets the relevant criteria, all or part of your publication costs will be covered. Please make sure your user information is up-to-date by logging into Editorial Manager at Editorial Manager® and clicking the ‘Update My Information' link at the top of the page. For questions related to billing, please contact billing support.

If your institution or institutions have a press office, please notify them about your upcoming paper to help maximize its impact. If they’ll be preparing press materials, please inform our press team as soon as possible -- no later than 48 hours after receiving the formal acceptance. Your manuscript will remain under strict press embargo until 2 pm Eastern Time on the date of publication. For more information, please contact onepress@plos.org.

Kind regards,

Jamshed Akhtar, MBBS, FCSP, FRCS, MHPE, FACS, M Bioethics

Academic Editor

PLOS One

Additional Editor Comments (optional):

Thank you for submitting second revision where point wise answers are provided. Revised manuscript shows track changes in the previous version that was easy to understand.

Reviewers' comments:

Reviewer's Responses to Questions

**Comments to the Author**

Reviewer #3: All comments have been addressed

**********

2. Is the manuscript technically sound, and do the data support the conclusions?

Reviewer #3: Yes

**********

3. Has the statistical analysis been performed appropriately and rigorously?

Reviewer #3: Yes

**********

4. Have the authors made all data underlying the findings in their manuscript fully available?

The PLOS Data policy

Reviewer #3: Yes

**********

5. Is the manuscript presented in an intelligible fashion and written in standard English?

Reviewer #3: Yes

**********

Reviewer #3: I have reviewed the revised manuscript submitted by the author and carefully examined the corrections made. I am pleased to inform you that the author has addressed the comments and suggestions satisfactorily.

**********

what does this mean?). If published, this will include your full peer review and any attached files.

If you choose “no”, your identity will remain anonymous but your review may still be made public.

**Do you want your identity to be public for this peer review?**  For information about this choice, including consent withdrawal, please see our Privacy Policy

Reviewer #3: No

**********

# Editor report: Acceptance letter

**DOI:** 10.1371/journal.pone.0360643.r006

**Authors:** Jamshed Akhtar

PONE-D-26-23135R2

PLOS One

Dear Dr. Wu,

I'm pleased to inform you that your manuscript has been deemed suitable for publication in PLOS One. Congratulations! Your manuscript is now being handed over to our production team.

At this stage, our production department will prepare your paper for publication. This includes ensuring the following:

* All references, tables, and figures are properly cited

* All relevant supporting information is included in the manuscript submission,

* There are no issues that prevent the paper from being properly typeset

You will receive further instructions from the production team, including instructions on how to review your proof when it is ready. Please keep in mind that we are working through a large volume of accepted articles, so please give us a few days to review your paper and let you know the next and final steps.

Lastly, if your institution or institutions have a press office, please let them know about your upcoming paper now to help maximize its impact. If they'll be preparing press materials, please inform our press team within the next 48 hours. Your manuscript will remain under strict press embargo until 2 pm Eastern Time on the date of publication. For more information, please contact onepress@plos.org.

You will receive an invoice from PLOS for your publication fee after your manuscript has reached the completed accept phase. If you receive an email requesting payment before acceptance or for any other service, this may be a phishing scheme. Learn how to identify phishing emails and protect your accounts at https://explore.plos.org/phishing.

If we can help with anything else, please email us at customercare@plos.org.

Thank you for submitting your work to PLOS One and supporting open access.

Kind regards,

PLOS One Editorial Office Staff

on behalf of

Dr. Jamshed Akhtar

Academic Editor

PLOS One
